# Maternal Serum and Placental Metabolomes in Association with Prenatal Phthalate Exposure and Neurodevelopmental Outcomes in the MARBLES Cohort

**DOI:** 10.3390/metabo12090829

**Published:** 2022-09-02

**Authors:** Mariana Parenti, Rebecca J. Schmidt, Sally Ozonoff, Hyeong-Moo Shin, Daniel J. Tancredi, Paula Krakowiak, Irva Hertz-Picciotto, Cheryl K. Walker, Carolyn M. Slupsky

**Affiliations:** 1 Department of Nutrition, University of California, Davis, CA 95616, USA; 2 Department of Public Health Sciences, University of California, Davis, CA 95616, USA; 3 Medical Investigation of Neurodevelopmental Disorders (MIND) Institute, University of California, Davis, CA 95616, USA; 4 Department of Psychiatry and Behavioral Sciences, University of California, Davis, CA 95616, USA; 5 Department of Environmental Science, Baylor University, Waco, TX 76798, USA; 6 Department of Pediatrics, School of Medicine, University of California, Davis, CA 95616, USA; 7 Department of Obstetrics & Gynecology, School of Medicine, University of California, Davis, CA 95616, USA; 8 Department of Food Science and Technology, University of California, Davis, CA 95616, USA

**Keywords:** autism, phthalates, prenatal exposure, NMR, placenta, serum

## Abstract

Prenatal exposure to phthalates, a family of endocrine-disrupting plasticizers, is associated with disruption of maternal metabolism and impaired neurodevelopment. We investigated associations between prenatal phthalate exposure and alterations of both the maternal third trimester serum metabolome and the placental metabolome at birth, and associations of these with child neurodevelopmental outcomes using data and samples from the Markers of Autism Risk in Babies Learning Early Signs (MARBLES) cohort. The third trimester serum (*n* = 106) and placental (*n* = 132) metabolomes were investigated using ^1^H nuclear magnetic resonance spectroscopy. Children were assessed clinically for autism spectrum disorder (ASD) and cognitive development. Although none of the urinary phthalate metabolite concentrations were associated with maternal serum metabolites after adjustment for covariates, mixture analysis using quantile g-computation revealed alterations in placental metabolites with increasing concentrations of phthalate metabolites that included reduced concentrations of 2-hydoxybutyrate, carnitine, *O*-acetylcarnitine, glucitol, and *N*-acetylneuraminate. Child neurodevelopmental outcome was not associated with the third trimester serum metabolome, but it was correlated with the placental metabolome in male children only. Maternal phthalate exposure during pregnancy is associated with differences in the placental metabolome at delivery, and the placental metabolome is associated with neurodevelopmental outcomes in males in a cohort with high familial ASD risk.

## 1. Introduction

Phthalates are a family of plasticizers that can easily leach into the environment [1]. They are manufactured in products that humans interact with every day, such as vinyl building materials, food packaging, and personal care products, but are also found in house dust and food. Phthalates are endocrine-disrupting compounds that have been linked with differences in hormone levels in humans, including insulin, sex hormones, and thyroid hormones [2,3,4,5,6]. Moreover, phthalate exposure during gestation has been associated with impaired neurodevelopmental outcomes in humans [7]. Phthalate exposure—whether through transdermal absorption, inhalation, or diet—is usually measured based on urinary concentrations of phthalate monoesters and/or secondary oxidation products [8]. The fact that individual phthalate metabolites have been detected in greater than 89% of samples from 11,071 National Health and Nutrition Examination Survey (NHANES) participants suggests that phthalate exposure is ubiquitous in the U.S. [9]. In an analysis of chemical exposure among 268 pregnant women in NHANES, at least four phthalate metabolites were measured in each pregnant woman, with a median of nine phthalate metabolites detected in each subject [10]. More recent studies have detected some phthalate metabolites in 100% of samples from 205 pregnancies in California [11] and in 171 pregnancies in the United States and Puerto Rico [12]. Furthermore, the level of exposure tends to be higher in historically marginalized communities, including non-Hispanic Black and Hispanic women [13,14]. In addition to direct maternal exposure, phthalates and their metabolites have also been measured in amniotic fluid, cord blood, and breastmilk, indicating that exposure begins in utero and continues through early life [15,16,17]. In addition to endocrine disruption, phthalate exposure is known to induce oxidative stress in humans [18,19,20,21]. A delicate balance of oxidation is necessary for normal development [22], where low levels of reactive oxygen species act as signaling molecules. However, upsetting this balance has been associated with neurodevelopmental disorders including autism spectrum disorder (ASD) [23].

Urinary phthalate metabolites measured during gestation, including metabolites of di-2-ethylhexyl phthalate (DEHP), di-n-butyl phthalate (DnBP), and di-n-octyl phthalate (DnOP), have been associated with increased autistic traits [24,25,26]; however, some studies have shown no correlation between phthalate exposure and ASD [27,28]. There could be several reasons for this. For instance, ASD requires clinical training to assess, and many studies do not use gold-standard tools, such as the ASD Diagnostic Observation Schedule. Symptoms of autism are variable and can be difficult to diagnose, particularly in young children [29,30,31,32]. It could also be that phthalate exposure itself does not contribute to ASD and that it may serve as a proxy for other exposures and/or maternal behaviors related to ASD risk [33], such as nutrition [34], and other environmental exposures, such as air pollution, pesticides, and other plastic additives [35]. On the other hand, mixed evidence for the association between prenatal phthalate exposure and ASD may be related to how the exposure is measured. Investigating exposures to phthalate mixtures could be important to understanding the relationship between prenatal phthalate exposure and ASD risk [25]; so too could investigating the timing of the exposure, as sensitive windows of phthalate exposure for ASD risk may exist during gestation and postnatal life [25,26]. It has also been hypothesized that phthalate exposure could impact ASD risk through genetic susceptibility to oxidative stress since phthalates have been shown to elicit oxidative stress [36]. While it is difficult to investigate ASD as a binary variable given its multifactorial nature [30], a metabolomics approach allows us to investigate intermediate phenotypes associated with phthalate exposure that could be relevant to ASD development.

We have previously investigated the association between prenatal phthalate exposure and neurodevelopmental outcomes and determined that individual phthalate metabolites alone were not associated with ASD in this same cohort of children at increased risk for ASD as part of the Markers of Autism Risk in Babies, Learning Early Signs (MARBLES) study [27]. However, this study did not investigate the subtle changes in metabolism associated with increased susceptibility to oxidative stress. Metabolomics, the study of small molecules, provides an unbiased view of metabolic disturbances that represent the downstream effects of an exposure’s influence on gene expression and protein activity. Phthalate exposure has been shown to induce changes in serum metabolism, as well as placental methylation and transcription in humans [37,38,39].

Despite the heterogeneity in ASD presentation, previous research has identified metabolic disturbances in both children with ASD and their mothers during pregnancy [40,41,42,43]. Among children with ASD, evidence of altered mitochondrial dysfunction, oxidative stress, one-carbon metabolism, and amino acid metabolism have been reported [40]. Multiple metabolic subgroups among children with ASD have also been described [41]. Disruptions in metabolites involved in one-carbon metabolism and the transsulfuration pathway reflected in maternal blood have also been observed during gestation [42], and ASD risk has also been associated with disturbances in biosynthetic and metabolic pathways related to glycosphingolipids, phospholipids, bile acids, pyrimidines, *N*-glycans, and steroid hormones in maternal serum collected in mid-pregnancy [43]. This is the first study to the best of our knowledge to investigate the third trimester maternal serum metabolome using proton nuclear magnetic resonance (^1^H NMR)-based metabolomics in relation to ASD. Additionally, the cohort includes children who did not develop ASD, but who were otherwise non-typically developing. A recent study of maternal third trimester plasma collected as part of the MARBLES study has identified alterations in the prostaglandin pathway related to non-typical development [44].

In the present analysis, we aimed to investigate the associations between (a) maternal phthalate exposure during pregnancy and maternal serum metabolites in the third trimester and placental metabolites at birth, and (b) the serum and placental metabolomes and neurodevelopmental outcomes using ^1^H NMR-based metabolomic analyses of samples collected in the MARBLES cohort. Indeed, maternal serum and placenta metabolite concentrations could indicate the availability of substrates necessary for fetal growth and development. For instance, reduced concentrations of amino acids might reflect reduced availability for building proteins necessary for growth and development, for both the fetus and the placenta. Although phthalate exposure is associated with changes in placental growth and development [45,46], this is also the first study to the best of our knowledge to investigate the associations between phthalate exposure and the placental metabolome. Furthermore, with 14 phthalate metabolites from 8 phthalate diesters, we had the opportunity to investigate the effects of exposure to different mixtures of phthalates.

## 2. Materials and Methods

### 2.1. Study Population

This study included participants from the MARBLES cohort [47]. In this ongoing prospective observational study, families with one or more previous children with ASD are enrolled prior to or early on during a subsequent pregnancy. Children from these pregnancies are followed and their behavior and cognitive development are assessed clinically. At approximately 3 years of age, the children are classified into three groups of neurodevelopmental outcomes: typically developing, ASD, and non-typically developing. MARBLES enrollment began in 2006, with placenta samples used in this analysis collected as early as 2007. Maternal serum sample collection as part of MARBLES began in 2009. Maternal serum and placenta samples from pregnancies where maternal urinary phthalate metabolites were measured during the second and third trimesters were selected [27]. Of 200 analyzed serum samples from 119 pregnancies (1 twin pregnancy), 108 samples (1 from a twin pregnancy) were collected in the third trimester. Only 22 samples were collected in the first trimester and 69 were collected in the second trimester. Given that serum samples from the second and third trimesters were only available for 59 subjects, we opted to investigate the association between phthalate exposure and the serum metabolome using only third trimester samples. Furthermore, this allowed us to maintain a temporal relationship between earlier phthalate exposure and later serum metabolism since phthalate exposure was measured during the second and/or third trimester. While there were 108 third trimester serum samples (from 106 maternal subjects, including 1 twin pregnancy) from unique pregnancies, we selected the sample from the first available pregnancy in these analyses or the first twin. Of the 144 placenta samples connected to 133 mothers, we selected the placenta sample from the first available pregnancy to ensure independence between samples. One of these samples was excluded because it was connected to data from twins of different sexes and the placenta is fetal tissue. Of these 106 serum samples and 132 placenta samples, 78 mother-child pairs provided both serum and placenta samples, 54 pairs provided only a placenta sample, and 28 provided only a serum sample.

### 2.2. Child Neurodevelopmental Assessment

At 3 years of age, the children of interest were clinically assessed for ASD using the gold-standard ASD Diagnostic Observation Schedule (ADOS) [48]. Cognitive, language, and motor development were also assessed using the Mullen Scales of Early Learning (MSEL) [49]. Scores on both the ADOS and MSEL at approximately 36 months were used to classify neurodevelopmental outcomes as previously described [50]. Children with ADOS scores equal to or greater than the ASD cutoff were classified as ASD (serum: *n* = 27; placenta: *n* = 33). Children without ASD but who had ADOS scores within 3 points of the ASD cutoff or MSEL subdomain scores 1.5 to 2 standard deviations below the mean were classified as non-typically developing (Non-TD; serum: *n* = 14; placenta: *n* = 17). The remaining participants who did not meet the criteria for ASD or Non-TD were classified as typically developing (TD; serum: *n* = 66; placenta: *n* = 83).

### 2.3. Urinary Phthalate Metabolite Analysis

Phthalate metabolite concentrations were measured in pooled urine samples collected during the second and/or third trimesters as previously described [27] using online solid phase extraction coupled with high-performance liquid chromatography with isotope dilution-tandem mass spectrometry. The 14 phthalate metabolites measured included: monoethyl phthalate (MEP), mono-isobutyl phthalate (MiBP), monohydroxy-isobutyl phthalate (MHiBP), mono-n-butyl phthalate (MBP), monohydroxy-n-butyl phthalate (MHBP), monobenzyl phthalate (MBzP), mono(2-ethylhexyl) phthalate (MEHP), mono(2-ethyl-5-hydroxyhexyl) phthalate (MEHHP), mono(2-ethyl-5-oxohexyl) phthalate (MEOHP), mono(2-ethyl-5-carboxypentyl) phthalate (MECPP), mono(3-carboxypropyl) phthalate (MCPP), mono-isononyl phthalate (MNP), mono-carboxyisooctyl phthalate (MCOP), and mono-carboxyisononyl phthalate (MCNP). Concentrations were adjusted for specific gravity to correct for dilution and reported as µg/L. Machine-observed concentrations were used for values below the limit of detection. Urinary concentrations of DEHP metabolites were divided by their molar weights and summed to calculate the molar sum of DEHP metabolites as a marker of total DEHP exposure (ΣDEHP = MEHP + MEHHP + MEOHP + MECPP; µmol/L). The distribution of phthalate metabolite concentrations is presented in Table S1.

### 2.4. Serum Sample Preparation and ^1^H NMR Metabolomics Analysis

Serum samples collected during the third trimester were used in this study. Whole blood was centrifuged, and serum was stored at −80 °C prior to extraction. Samples were thawed on ice and filtered using Amicon Ultra-0.5 mL 3000 MW centrifugal filters (Millipore, Burlington, MA, USA) which were prepared by washing three times with ultra-pure water to remove glycerol. The resulting filtrate containing the water-soluble (polar) metabolites was collected and its volume was adjusted with ultra-pure water if an insufficient sample was collected. An internal standard (Chenomx, Edmonton, AB, USA) containing 5.0 mM 3-(trimethylsilyl)-1-propanesulfonic acid-d6 (DSS-d6), 0.2% NaN_3_, and 99.8% D_2_O was added. Each sample’s pH was adjusted to 6.8 ± 0.1 and 180 µL was loaded into a 3 mm NMR tube (Bruker, Billerica, MA, USA). Samples were stored at 4 °C until spectral acquisition within the same day.

^1^H-NMR spectra were collected using a Bruker Avance 600 MHz spectrometer (Bruker, Billerica, MA, USA) using the noesypr1d pulse sequence. Spectra were acquired at 25 °C, with water saturation of 2.5 s during the prescan delay, a mixing time of 100 ms, a sweep width of 12 ppm, an acquisition time of 2.5 s, 8 dummy scans, and 32 transients. All spectra were zero-filled to 128 K data points and Fourier transformed with a 0.5 Hz line broadening applied. Spectra were manually phase- and baseline-corrected using Chenomx NMR Processor (v. 8.1, Chenomx, Edmonton, AB, USA), and metabolite concentrations were quantified using Chenomx Profiler (v. 8.1). The quantification relies on the internal standard (DSS-d6) to determine each metabolite’s concentration using a library of compound spectral signatures which allows for the absolute quantification of many compounds within a spectrum.

### 2.5. Placenta Sample Preparation and ^1^H NMR Metabolomics Analysis

Placentas were collected at delivery and full-thickness tissue sections were stored at −80 °C. All samples were partially-thawed and a 6 mm biopsy punch was used to aliquot samples for metabolic analyses which were again stored at −80 °C. Placenta samples were then cryoground to a uniform fine powder using mortars, pestles, and liquid nitrogen. The polar metabolites from each sample were extracted in a two-step CHCl_3_:MeOH:H_2_O extraction [51]. Approximately 80 mg of each cryoground sample was used and the upper layer containing polar metabolites was collected, measured, and frozen before being dried using a miVac concentrator system (Genevac, Warminster, PA, USA). The dried samples were stored at −80 °C until preparation for ^1^H NMR spectroscopy. Samples were reconstituted in 10 mM potassium phosphate buffer. The internal standard DSS-d6 was added and the samples were prepared for spectral acquisition and metabolite identification and quantification as described above for serum samples.

### 2.6. Statistical Analysis

All statistical analyses were conducted using the R statistical language (R Foundation for Statistical Computing, Vienna, Austria) with RStudio. Demographic differences between neurodevelopmental outcome groups were assessed using chi-squared tests for categorical variables, Fisher’s Exact test for categorical variables with empty cells, and Kruskal–Wallis tests for continuous variables. Serum metabolites were corrected for dilution and reported in µmol/L. Placenta metabolites were corrected and are reported in nmol/g tissue. Phthalate, placenta, and serum metabolites were log_10_-transformed to improve skewed distributions.

We identified and quantified 59 serum metabolites and 62 placental metabolites. However, metabolites that were identified in samples but may have been introduced during sample preparation (serum: ethanol, glycerol, and isopropanol; placenta: ethanol, isopropanol, and methanol) and metabolites that were not quantified in at least 20% of samples (serum: acetaminophen, ethyl-β-D-glucoronide, fructose, inosine, *N*-phenylacetylglycine, propylene glycol, trimethylamine *N*-oxide, and valproate; placenta: acetone, ascorbate, propylene glycol, and urea) were excluded from these analyses. Thus, we included 48 serum metabolites and 54 placental metabolites in this analysis, in conjunction with 14 urinary phthalate metabolites previously measured and described elsewhere [27]. Correlations between placental metabolite concentrations and serum metabolite concentrations are presented in Figure S1.

Univariate normality was assessed using the Shapiro–Wilks test and multivariate normality with Mardia’s test (package MNV). We used two approaches in evaluating the effects of phthalate exposure on the serum and placental metabolomes. In a substance-by-substance approach, correlations between urinary phthalate metabolites and either placenta metabolites or serum metabolites were evaluated using Spearman’s rank correlation coefficient and corrected for false discovery rate (FDR) using the Benjamini–Hochberg procedure [52]. Estimates of the total and direct effects of each phthalate metabolite on each placenta and serum metabolite were evaluated independently and adjusted for covariates using multiple linear regression (MLR) and FDR-corrected. In a mixture approach, the total and direct effects of phthalate mixture exposure on each placenta and serum metabolite were evaluated independently and adjusted for covariates using quantile g-computation (implemented using the package *qgcomp*) and FDR-corrected [53]. Quantile g-computation is useful for investigating the effect of an exposure mixture by estimating the effect of increasing all exposures by one quantile, adjusted for covariates [53]. In this analysis, phthalate exposure was split into quartiles, so each model estimates the change in the log_10_-transformed metabolite concentration after increasing all phthalate metabolites by 1 quartile (*ψ*).

The associations between neurodevelopmental outcome and either the serum metabolome or placental metabolome adjusted for covariates were assessed using permutation multivariate analysis of variance (PERMANOVA). The association between neurodevelopmental outcome and each serum or placenta metabolite was assessed using single-response PERMANOVA adjusted for covariates and FDR-corrected [54]. PERMANOVA was implemented using the package *vegan* [55]. We considered *p*-values (for multivariate analyses) less than 0.05 and FDR-corrected *p*-values less than 0.1 to be statistically significant.

Because the associations between birth year and phthalate exposure were closely tied in the placenta, we tested the relationship between birth year and placental metabolites in those subjects with low DEHP exposure by conducting MLR for associations between birth year and log_10_-transformed metabolite concentration after adjustment for gestational age at delivery, maternal metabolic condition, homeownership status, maternal race/ethnicity, and maternal education in subjects with a ΣDEHP less than the median (*n* = 66). As normality assumptions were not met, significance was tested by permuting the residuals under the reduced model (containing only the covariates) and regressing them on birth year [56].

Individual directed acyclic graphs (DAGs) were constructed in R (package *dagitty*) [57] to identify potential confounders. These potential covariates included maternal prenatal vitamin use during the first month of pregnancy, birth year, birth weight, maternal age, maternal educational attainment, maternal race/ethnicity, gestational age at sampling and/or at gestation, method of delivery, maternal metabolic condition, and child’s sex. Information about self-reported maternal prenatal vitamin use during the 6 months prior and throughout pregnancy was collected in the MARBLES cohort. Maternal prenatal vitamin use in the first month of pregnancy was used here because was inversely associated with ASD risk in the MARBLES cohort [58]. The maternal metabolic condition was coded to integrate maternal pre-pregnancy body mass index (BMI), hypertensive disorders (including preeclampsia), and diabetes during pregnancy with five levels: BMI ≤ 25 and no metabolic conditions [reference], 25 < BMI < 30 and no metabolic conditions, BMI ≥ 30 and no other metabolic conditions, any hypertensive disorder (without any diabetes) at any BMI, and diabetes at any BMI [59]. From the DAGs (Figure S2), we identified adjustment sets to reduce bias in the estimates of associations between (a) urinary phthalate metabolites and serum metabolites and (b) serum metabolites and neurodevelopmental outcomes. The minimal adjustment set to reduce bias in the estimates of the total effect of phthalate exposure on each serum metabolite included birth year, maternal education, maternal race/ethnicity, and homeownership status. We also identified the maternal metabolic condition as a potential mediator on the path between prenatal phthalate exposure and serum metabolite concentrations. The minimal adjustment set to reduce bias in the estimates of the direct effect of phthalate exposure on each serum metabolite included birth year, maternal race/ethnicity, homeownership status, and maternal metabolic condition. The minimal adjustment set to reduce bias in the estimates of the total effect of the serum metabolome on neurodevelopmental outcome included birth year, maternal race/ethnicity, homeownership status, and maternal metabolic condition. Additionally, fasted time and gestational age at sample collection were included in all adjusted models.

From the DAG (Figure S3), we identified adjustment sets to reduce bias in the estimates of associations between (a) urinary phthalate metabolites and placental metabolites and (b) placental metabolites and neurodevelopmental outcomes. The minimal adjustment set to reduce bias in the estimates of the total effect of phthalate exposure on each placental metabolite included birth year, maternal education, maternal race/ethnicity, and homeownership status. We also identified maternal metabolic condition, delivery mode, and gestational age at delivery as potential mediators on the path between prenatal phthalate exposure and placental metabolite concentrations. The minimal adjustment set to reduce bias in the estimates of the direct effect of phthalate exposure on each placental metabolite included birth year, maternal education, maternal race/ethnicity, homeownership status, maternal metabolic condition, delivery mode, and gestational age at delivery. The minimal adjustment set to reduce bias in the estimates of the total effect of the placental metabolome on neurodevelopmental outcome included birth year, birth weight, maternal race/ethnicity, homeownership status, maternal metabolic condition, maternal age, prenatal vitamin use in the first month of pregnancy, delivery mode, and gestational age at delivery.

## 3. Results

Table 1 summarizes the demographic characteristics of the mothers and children studied grouped by the child’s neurodevelopmental outcome. No significant differences were observed in the subpopulation in which the serum metabolome was examined. However, among subjects providing placenta samples, the child’s neurodevelopmental outcome was significantly associated with prenatal vitamin use in the first month of pregnancy and gestational age at delivery (*p* < 0.05).

### 3.1. Associations between Phthalate Metabolites and Serum Metabolites

**Figure 1:**
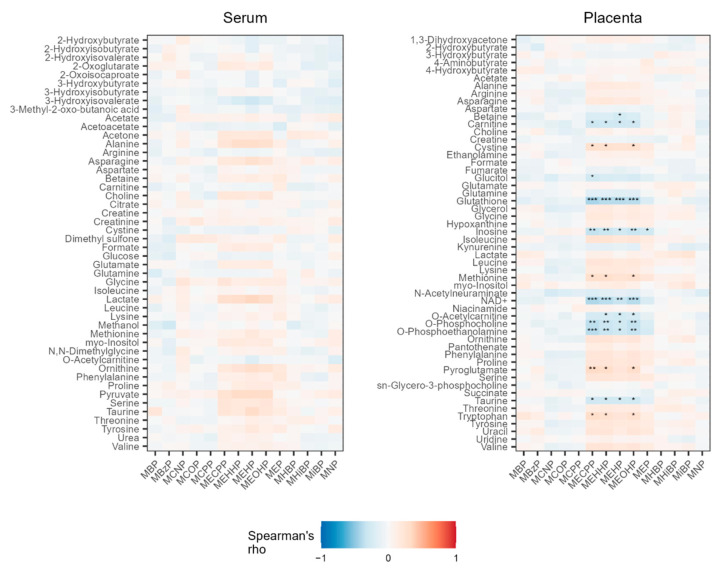
Spearman correlations between phthalate metabolites and serum metabolites (**left**) and placenta metabolites (**right**). Positive associations (red) identify metabolites where higher urinary phthalate metabolite concentrations correlated with higher metabolite concentrations, while negative associations (blue) identify metabolites where higher urinary phthalate metabolite concentrations correlated with lower metabolite concentrations. Metabolites that remained significant after FDR correction are marked with stars. For FDR-corrected *p* < 0.001, ***; *p* < 0.01, **; and *p* < 0.05, *.

Spearman correlations were used to explore the associations between urinary phthalate metabolites and serum metabolites. After FDR correction, no associations remained significant (Figure 1). MLR was also conducted to estimate the total and direct effects of individual urinary phthalate metabolites on individual serum metabolites after adjustment for covariates. The minimal adjustment set to reduce bias in the estimates of the total effect of phthalate exposure on each serum metabolite included birth year, maternal education, maternal race/ethnicity, and homeownership status. The minimal adjustment set to reduce bias in the estimates of the direct effect of phthalate exposure on each serum metabolite included birth year, maternal race/ethnicity, homeownership status, and maternal metabolic condition. Additionally, fasted time and gestational age at sample collection were included in all adjusted models. We observed no significant total nor direct estimated effects of individual phthalate metabolites on individual serum metabolites following FDR correction (Table S2). Similarly, mixture analysis using quantile g-computation did not reveal significant total or direct estimated effects of phthalates on serum metabolites after FDR correction (Table S3).

### 3.2. Associations between Serum Metabolites and Neurodevelopmental Outcomes

PERMANOVA was used to explore the association between neurodevelopmental outcome and the serum metabolome, and no association was observed (*R*^2^ = 0.0165, *p* = 0.6129 under 9999 permutations), nor was an association observed after adjustment for covariates, which included birth year, maternal race/ethnicity, homeownership status, maternal metabolic condition, fasted time, and gestational age at sample collection (Table S4, *R*^2^ = 0.0144, *p* = 0.7125 under 9999 permutations). Furthermore, in single-response PERMANOVAs exploring the association between neurodevelopmental outcome and individual serum metabolites, only cystine was elevated in mothers carrying a child later diagnosed with ASD. Post hoc pairwise comparisons revealed that cystine concentrations were elevated in the ASD group compared to the TD group (*p* = 0.0357). However, this association did not survive FDR correction (Table S5). Stratifying analysis on the child of interest’s sex similarly showed null results across both sexes (Table S4).

### 3.3. Associations between Phthalate Metabolites and Placenta Metabolites

Spearman correlations were used to explore the associations between urinary phthalate metabolites and placenta metabolites. After FDR correction, only the DEHP metabolites MEHP, MEHHP, MEOHP, and MECPP remained significantly associated with multiple placenta metabolites (Figure 1). They were weakly to moderately positively correlated with cystine, methionine, pyroglutamate, and tryptophan and weakly to moderately negatively correlated with betaine, glutathione, inosine, NAD^+^, *O*-acetylcarnitine, *O*-phosphocholine, and *O*-phosphoethanolamine. Additionally, MEP was also negatively associated with inosine. We also used MLR to estimate the total and direct effects of individual urinary phthalate metabolites on individual placental metabolites after adjustment for covariates. The minimal adjustment set to reduce bias in the estimates of the total effect of phthalate exposure on each placental metabolite included birth year, maternal education, maternal race/ethnicity, and homeownership status. The minimal adjustment set to reduce bias in the estimates of the direct effect of phthalate exposure on each placental metabolite included birth year, maternal education, maternal race/ethnicity, homeownership status, maternal metabolic condition, delivery mode, and gestational age at delivery. However, we did not observe significant estimates of the total or direct effects of individual phthalate metabolites on individual placental metabolites after FDR correction (Table S6).

We employed mixture analysis using quantile g-computation to investigate the total and direct estimated effects of phthalates on placental metabolites using the same covariates (Table 2). After FDR correction, we observed a significant, negative total estimated effect of phthalates on placental glucitol (also known as sorbitol), carnitine, *O*-acetylcarnitine, *N*-acetylneuraminate (also known as sialic acid), and 2-hydroxybutyrate (Table 2). We observed significant, negative direct effect estimates of the phthalate mixture on placental glucitol, carnitine, *O*-acetylcarnitine, *N*-acetylneuraminate, and 2-hydroxybutyrate (Table 2).

### 3.4. Associations between Placenta Metabolites and Birth Year

Birth year and phthalate exposure, especially to DEHP, are inversely related in this cohort. Since adjustment for birth year attenuated the associations between phthalate exposure and placental metabolites, we tested the association between birth year and placental metabolites in those subjects with low DEHP exposure. In subjects with a molar sum of DEHP metabolites less than or equal to the median (*n* = 66), we conducted MLR to test the association between birth year and log_10_-transformed metabolite concentration after adjustment for gestational age at delivery, homeownership status, maternal metabolic condition, maternal race/ethnicity, and maternal education (Table 3 and Table S7). After FDR correction, creatine, glutathione, inosine, NAD^+^, *O*-phosphocholine, and *O*-phosphoethanolamine were positively associated with birth year, while arginine, cystine, isoleucine, leucine, lysine, methionine, ornithine, phenylalanine, pyroglutamate, serine, tryptophan, tyrosine, and uracil were significantly negatively associated with the birth year (Table 3, FDR *p* < 0.1). Additional associations with the birth year for metabolites with FDR *p* > 0.1 are reported in the Supplementary Materials (Table S7).

### 3.5. Associations between Placenta Metabolites and Neurodevelopmental Outcomes

PERMANOVA was used to explore the association between neurodevelopmental outcomes and the placental metabolome. In an unadjusted analysis, a small correlation was observed (*R*^2^ = 0.0289, *p* = 0.0303 under 9999 permutations). However, no association was observed after adjustment for covariates, including birth year, birth weight, maternal race/ethnicity, homeownership status, maternal metabolic condition, maternal age, prenatal vitamin use in the first month of pregnancy, delivery mode, and gestational age at delivery (Table 4, *R*^2^ = 0.0201, *p* = 0.1270 under 9999 permutations). When stratifying the analysis by the child’s sex, we observed that the placental metabolome was associated with neurodevelopmental outcome after adjustment for covariates in males (Table 4, *R*^2^ = 0.0440, *p* = 0.0451 under 9999 permutations). Post hoc pairwise PERMANOVAs of the placental metabolomes from male children corrected for multiple testing showed that the ASD and TD groups tended to differ (*R*^2^ = 0.0361, *p* = 0.0705), but that the Non-TD group did not differ significantly from the others (*p* < 0.1).

We also conducted single-response PERMANOVAs to investigate the association between placenta metabolites and neurodevelopmental outcomes in all children and stratified by sex. Overall, concentrations of niacinamide, taurine, and uridine differed by the neurodevelopmental outcome after adjustment for covariates (Table S8). While the post hoc pairwise testing revealed no difference in niacinamide concentrations between groups, taurine and uridine concentrations were lower in the ASD group than in the TD group (taurine, *p* = 0.0567; uridine, *p* = 0.0255). However, these associations did not survive FDR correction (Table S8). In female children, concentrations of choline, glycerol, and taurine were associated with different neurodevelopmental outcomes after adjustment for covariates (Table S8). In the post hoc pairwise comparisons, we found that choline and glycerol concentrations were lower in ASD compared to TD (choline: *p* = 0.0447; glycerol, *p* = 0.0492). Taurine concentrations were lower in ASD compared to both the TD (*p* = 0.0228) and Non-TD groups (*p* = 0.0419). However, these associations did not survive FDR correction (Table S8). In male children, concentrations of alanine, cystine, ethanolamine, fumarate, hypoxanthine, methionine, NAD^+^, pyroglutamate, and uridine differed by the neurodevelopmental outcome after adjustment for covariates (Table S8). No pairwise group differences in ethanolamine, hypoxanthine, or pyroglutamate concentrations were observed in the post hoc pairwise comparisons. Alanine, cystine, fumarate, methionine and uridine concentrations were lower in the ASD group than in TD group (alanine, *p* = 0.0702; cystine, *p* = 0.0645; fumarate, *p* = 0.0222; methionine, *p* = 0.0978; and uridine, *p* = 0.0096), while NAD^+^ concentrations were higher in the ASD group than the TD group (*p* = 0.0045). However, these associations also did not survive FDR correction (Table S8).

## 4. Discussion

In this study, we investigated the associations between prenatal phthalate exposure and the maternal serum metabolome during the third trimester and the placental metabolome at birth. In the placenta, we observed that phthalates were negatively associated with 2-hydroxybutyrate, carnitine, glucitol, *O*-acetylcarnitine, and *N*-acetylneuraminate concentrations. We also observed that neurodevelopmental outcome was associated with the placental metabolome in male children, though associations with individual metabolites did not survive FDR correction. However, we did not observe that the maternal serum metabolome was related to either phthalate exposure or neurodevelopmental outcome.

### 4.1. Associations with Phthalate Exposure

In the U.S. generally, and this cohort specifically, phthalate exposure patterns have changed over the course of the study (2007–2014) [9,11]. In the MARBLES cohort, exposure to the phthalate diesters butyl benzyl phthalate, dibutyl phthalate, DEHP, and diethyl phthalate have decreased, while exposures to di-isobutyl phthalate, di-isononyl phthalate, and DnOP have increased [11]. Thus, there are differing profiles of phthalate exposure over the course of the study. While many studies report only individual associations or investigate the effects of phthalate exposure substance-by-substance, phthalate exposure generally occurs as a combination of several phthalates together with other environmental toxicants. These profiles are important to consider because chemical mixtures may exert different biological effects compared to each component substance alone [60]. Furthermore, the pattern of urinary phthalate metabolites may point to differences in phthalate metabolism and excretion between individuals. In the case of DEHP, a higher ratio of its metabolically active monoester MEHP to its secondary oxidized metabolites (MEHHP, MEOPH, and MECPP) is associated with greater physiological effects [61]. Thus, analysis of the mixture provides information about both environmental phthalate exposure and individual phthalate metabolism relevant to the physiological effects.

We took two approaches to analyze the relationship between phthalate exposure and the placental metabolome. First, in a substance approach, we did not observe significant effects of individual phthalate metabolites on individual placental metabolites after adjusting for covariates. However, when we applied mixture analysis, we found that phthalates were negatively associated with 2-hydroxybutyrate, carnitine, glucitol, *O*-acetylcarnitine, and *N*-acetylneuraminate concentrations after adjustment for covariates.

Carnitine and acylcarnitines are classically involved in lipid transport into the mitochondria and subsequent metabolism [62]. Exposure to DEHP and its metabolites has been associated with elevated lipid concentrations in rat trophoblasts [63] and altered placental lipid metabolism [64]. Thus, lower carnitine and *O*-acetylcarnitine concentrations with increasing exposure to the phthalate mixture could reflect impaired placental lipid transport and might suggest, in conjunction with evidence of oxidative stress, impaired mitochondrial function. Moreover, carnitine increases cell proliferation and amino acid transporter expression and abundance through insulin-like growth factor I signaling in rat trophoblasts [65]. Carnitine is essential to fetal development [66]. During pregnancy, maternal circulating carnitine levels drop in part due to placental transfer to the fetus via carnitine/organic cation transporter 2 (OCTN2) [67]. The placenta cannot synthesize carnitine, so OCTN2-mediated transport is critical [68]. However, under hypoxic conditions such as preeclampsia, placental OCTN2 abundance and carnitine concentrations decrease [69]. Prenatal phthalate exposure has been associated with an increased risk of pregnancy-induced hypertensive disorders, including preeclampsia [70,71]. The lower placental concentrations of *O*-acetylcarnine and carnitine with increasing exposure to phthalates could point to altered placental function.

Carnitine can also act as an osmolyte [72]. Glucitol is a polyol and an osmolyte produced by the placenta [73]. In early development, the polyol pathway is an important source of energy in a low-oxygen environment [74]. However, placental glucitol concentrations increase with gestational age [75] after the placenta becomes the site of fetal gas exchange. Similar to other highly metabolic organs, the placenta has relatively high osmolarity [75]. While we did not observe changes in the concentrations of other osmolytes (including taurine and *myo*-inositol) in response to the phthalate mixture, changes in glucitol concentrations could indicate changes in metabolism.

*N*-Acetylneuraminate, also known as sialic acid, has a role in neurodevelopment, immune function, and angiogenesis. Sialic acid is an essential nutrient in neurodevelopment [76]. In rats, maternal prenatal supplementation with sialic acid improved offspring cognition and resulted in elevated concentrations of sialic acid in the brain [77]. However, we did not observe that placental sialic acid was associated with ASD or Non-TD risk. In the placenta, sialic acid is essential starting in early pregnancy for protecting the conceptus from detection and destruction by the maternal innate immune response [78]. Decreased levels of placental sialyated oligosaccharides have been reported in both spontaneous and recurrent miscarriages [79] and preeclampsia [80]. Furthermore, sialic acid has been implicated in regulating angiogenesis. The angiogenic vascular endothelial growth factor must be sialyated in order to bind to its receptor [81], which is expressed in the placenta [68]. In the placenta, angiogenesis continues throughout pregnancy and is critical for increasing blood flow (and thus nutrients) to the fetus in the third trimester [82]. Phthalates have been associated with markers of impaired placental angiogenesis in human and animal studies [83]. In our study, sialic acid concentrations decreased as exposure to phthalates increased, suggesting that phthalate exposure may be related to impaired placental function.

2-Hydroxybutyrate is produced through methionine and threonine catabolism, as well as through the production of cysteine via the transsulfuration pathway [84]. Under conditions of oxidative stress, glutathione biosynthesis increases, and cysteine may become limiting. Thus, as a by-product of cysteine production through glutamate-cysteine ligase, 2-hydroxybutyrate is a sign of glutathione synthesis and oxidative stress [85]. DEHP has been shown to reduce glutathione concentrations in mouse kidneys through the depletion of transcription factor nuclear factor erythroid 2-related factor 2 (Nrf2) and glutamate-cysteine ligase abundance [86]. Nrf2 has been identified as a potential target to improve antioxidant defense in exposure to DEHP and other phthalates [87]. In our analysis, placental glutathione concentrations were negatively associated with increased phthalate concentrations prior to FDR correction. Thus, the negative association between the phthalates and 2-hydroxybutyrate may reflect reduced glutathione biosynthesis.

While concentrations of these metabolites were not significantly or strongly associated with birth year, we identified birth year as a potential confounder in our analysis. Our samples were stored at −80 °C, though some samples had been stored for over a decade before extraction. Cold storage, even at −80 °C, is associated with plasma sample degradation over time [88]. While the human EDTA plasma metabolome is relatively stable in samples stored for up to 7 years, amino acids, lipids, and energy metabolites have been shown to be sensitive to additional storage time at −80 °C [88], and thus we adjusted for the birth year in our serum analyses. While similar analyses have not been carried out in placenta samples, we observed similar effects when we reframed the birth year as a marker of storage time. With an earlier birth year, and thus increased storage time, we observed decreasing levels of the antioxidants inosine and glutathione, energy metabolite NAD^+^, and *O*-phosphocholine and *O*-phosphoethanolamine, as well as increasing levels of the glutathione breakdown-product pyroglutamate and cystine, the oxidized form of cysteine. We also observed increasing concentrations of many amino acids and uracil, potentially indicating the breakdown of macromolecules. It is also possible that over the course of the study, sample processing time improved. Previously, time delay in sample processing was shown to alter metabolite concentrations in placenta samples [89,90]. It is possible that these differences in metabolite concentrations are a sign of oxidation or degradation of the samples during processing and/or storage. So, while the birth year is associated with changes in phthalate exposure, it is also associated with changes in the placental metabolome. Thus, especially for metabolites related to oxidative stress, the potential effects of phthalate exposure could not be disentangled from the potential effects of birth year and long-term storage.

In this analysis, we did not observe an effect of phthalate exposure on the maternal third trimester serum metabolome. In our analysis of the serum metabolome, samples were collected throughout the third trimester. Over the course of pregnancy, serum concentrations of some amino acids increase, such as glutamine and methionine, while others decrease, such as leucine and valine [91]. Indeed, in this cohort, we observed a significant effect of gestational age at sample collection on the serum metabolome. Furthermore, participants did not fast before the serum was collected which contributed to variation in the serum metabolome. While we were able to adjust for time since the last meal or snack, we were not able to account for the potential effects of the size or nutrient composition of the most recent intake.

### 4.2. Associations with Neurodevelopmental Outcome

While phthalate exposures have been changing, ASD incidence has increased, due in part to increased awareness and evolving diagnostic criteria. However, these changes do not completely explain the increase [92]. ASD is highly heritable, but approximately 20% of the risk is environmental [93]. In a previous analysis of the effect of phthalate exposure on the neurodevelopmental outcome, phthalate exposure was not associated with ASD but some phthalate metabolites were associated with Non-TD [27]. We aimed to determine if differences in maternal serum metabolism were associated with neurodevelopmental outcomes and did not observe any associations.

Since the MARBLES cohort consists of younger siblings of children with ASD, the risk of ASD in this cohort is enriched [94]. This might partly explain why we did not observe significant associations between the maternal serum metabolome (or the individual metabolites that constitute it) and neurodevelopmental outcomes. Previous analyses of the maternal serum or plasma polar metabolome have identified metabolites and metabolic pathways associated with their child’s neurodevelopment, but these studies were conducted within the general population or explicitly included mothers with lower risk. In a study comparing plasma from pregnant women in the MARBLES cohort (at high risk of having a subsequent child with ASD) and women at low risk of having a subsequent child with ASD, metabolites related to 1-carbon metabolism and the transsulfuration pathways were useful in predicting the maternal risk group [42]. However, within the high-risk group from the MARBLES cohort, the maternal plasma metabolome was not predictive of ASD development in the child [42]. Similarly, metabolites related to one-carbon metabolism and the transsulfuration pathway differed in blood collected from non-pregnant mothers of children with and without ASD [95]. In an analysis of maternal serum samples collected around the 16th week of gestation as part of the California Prenatal Screening Program, metabolites related to glycosphingolipid, pyrimidine, bile acid, and steroid hormone metabolism differed between mothers of children with ASD and controls [43]. A more recent analysis of maternal third trimester plasma within the MARBLES cohort identified several lipid metabolites—related to fatty acid biosynthesis and essential fatty acid metabolism—negatively associated with Non-TD risk after FDR correction [44]. However, associations between polar metabolites positively associated with ASD risk and negatively associated with Non-TD risk did not survive FDR correction [44]. We speculate that in this high-familial risk cohort, maternal metabolic phenotype might be more similar than among mothers with low risk or that genetic contributions to ASD risk may outweigh metabolic contributions.

We also investigated associations between the placental metabolome and neurodevelopmental outcomes using PERMANOVA. The placenta, through its roles from nutrient transport to neurotransmitter biosynthesis, is known to influence fetal brain development [96]. We observed that the placental metabolome and neurodevelopmental outcome were associated in male children when we stratified by sex, though individual metabolite concentrations did not differ significantly between groups after FDR correction. However, males are at increased risk of neurodevelopmental disorders, including ASD [97]. Several theories to explain this sex bias have been put forward. One of these is the female protective effect theory, which holds that females have a higher threshold for developing a neurodevelopmental disorder and thus require more risks or exposures than males [98]. The biological underpinnings of a protective effect are yet unclear, but roles for the sex chromosomes and sex hormones have been proposed [98]. Fetal sex also plays a role in the development and function of the placenta, which shares its sex with the fetus as fetal tissue [99]. Males are at higher risk of a variety of adverse pregnancy and birth outcomes, including intrauterine growth restriction, preterm birth, and gestational diabetes [100,101]. Furthermore, males tend to experience more adverse effects during pregnancy than do similarly exposed females [101]. Sex differences in placental development have been implicated in sex differences in neurodevelopment through the placenta-brain axis [96]. Thus, the association between neurodevelopmental outcome and the placental metabolome only in males could be tied to sex differences in how the placenta processes exposures.

### 4.3. Limitations

These findings are from an enriched risk cohort for ASD, so they may not be generalizable to the broader population without a family history of ASD. Given that ASD is highly heritable, and this cohort is enriched for this heritable risk by using younger siblings, differences in maternal serum metabolism related to neurodevelopment might not be observed in this dataset. Additionally, while we considered a variety of potential confounding factors in this study, we could not rule out potential confounding by unmeasured factors, including other environmental toxicants. Indeed, pregnant women in the U.S. are exposed to a cocktail of chemical analytes from multiple chemical classes, including phthalates, pesticides, polybrominated diphenyl ethers, and toxic metals [10,12]. It is also possible that the phthalate measurement did not fully capture phthalate exposure over the course of pregnancy. In the MARBLES cohort, within-subject variability for urinary phthalate metabolites collected across the second and third trimester varied by subject and metabolite [102]. Furthermore, phthalate exposures are known to change over the course of pregnancy [103,104]. Finally, the limitations related to sample processing, storage, and other potential sources of variability might obscure potential associations between the serum and placental metabolomes and neurodevelopmental outcomes. In combination with the relatively small sample size, these sources of variability may have reduced our ability to detect subtle differences in metabolite concentrations that may be detected in larger studies.

## 5. Conclusions

Prenatal exposure to phthalate mixtures is related to changes in placental metabolism. Larger studies will be necessary to understand the metabolic and developmental effects of phthalate mixtures as sources and patterns of exposure are poised to change. We observed an association between placental metabolites and phthalate metabolite concentrations, despite the important effect of the birth year on the placental metabolome. Further, we observed that the placental metabolome was associated with neurodevelopmental outcomes in males. Additional studies investigating the associations between placental metabolism, prenatal phthalate exposures, and neurodevelopmental outcomes are warranted.

## Figures and Tables

**Table 1:** Demographic characteristics of study subjects by the child of interest’s neurodevelopmental outcome: typically developing (TD), ASD, and non-typically developing (Non-TD). For each neurodevelopmental outcome, the mean ± standard deviation for continuous variables and proportion (count) for categorical variables are reported. Differences in maternal and child factors between neurodevelopmental groups were assessed using chi-squared tests for categorical variables (or Fisher’s exact test, indicated by *) and Kruskal–Wallis tests for continuous variables.

	Serum				Placenta			
	TD (*n* = 66)	ASD (*n* = 26)	Non-TD (*n* = 14)	*p*	TD (*n* = 83)	ASD (*n* = 32)	Non-TD (*n* = 17)	*p*
Maternal age at birth, years	35 ± 5	36 ± 5	34 ± 4	0.38	35 ± 5	35 ± 5	33 ± 4	0.40
Birth weight, g	3412 ± 448	3465 ± 428	3287 ± 268	0.20	3435 ± 486	3445 ± 443	3344 ± 378	0.81
Birth year	2011 ± 2	2011 ± 2	2012 ± 2	0.53	2010 ± 2	2010 ± 2	2010 ± 2	0.15
Pre-pregnancy BMI	25.7 ± 5.0	27.1 ± 7.4	27.6 ± 10.5	0.85	26.3 ± 6.0	28.1 ± 8.2	29.2 ± 9.8	0.45
Time since last meal or snack, minutes	102 ± 75	101 ± 66	94 ± 68	0.95	-	-	-	-
Gestational age at birth, weeks	38.8 ± 1.5	39.4 ± 0.8	39.1 ± 0.9	0.35	38.9 ± 1.3	39.6 ± 1.0	38.9 ± 1.3	0.04
Gestational age at serum collection, days	233 ± 19	230 ± 18	238 ± 19	0.50	-	-	-	-
Prenatal vitamin use in the first month of pregnancy	36 (54.5%)	11 (42.3%)	8 (57.1%)	0.52	50 (60.2%)	10 (31.2%)	8 (47.1%)	0.02
Male child	34 (51.5%)	18 (69.2%)	8 (57.1%)	0.30	43 (51.8%)	23 (71.9%)	10 (58.8%)	0.15
Cesarean delivery	27 (40.9%)	9 (34.6%)	4 (28.6%)	0.64	48 (57.8%)	24 (75%)	11 (64.7%)	0.23
Delivery payer: Government	16 (24.2%)	6 (23.1%)	4 (28.6%)	0.87	17 (20.5%)	7 (21.9%)	6 (35.3%)	0.34
Homeowner	42 (63.6%)	15 (57.7%)	8 (57.1%)	0.82	47 (56.6%)	17 (53.1%)	11 (64.7%)	0.74
At least a bachelor’s degree	37 (56.1%)	13 (50%)	5 (35.7%)	0.37	49 (59%)	14 (43.8%)	5 (29.4%)	0.05
Maternal race/ethnicity				0.43				0.44
Non-Hispanic, White	36 (54.5%)	14 (53.8%)	5 (35.7%)		50 (60.2%)	16 (50%)	8 (47.1%)	
Historically marginalized groups								
Black/African-American	1 (1.5%)	1 (3.8%)	2 (14.3%)		3 (3.6%)	3 (9.4%)	2 (11.8%)	
Asian	12 (18.2%)	5 (19.2%)	3 (21.4%)		11 (13.3%)	4 (12.5%)	3 (17.6%)	
Hispanic, white	14 (21.2%)	4 (15.4%)	3 (21.4%)		17 (20.5%)	7 (21.9%)	3 (17.6%)	
Hispanic, non-white	1 (1.5%)	1 (3.8%)	1 (7.1%)		0 (0%)	1 (3.1%)	1 (5.9%)	
Multi-racial	2 (3.0%)	1 (3.8%)	0 (0%)		2 (2.4%)	1 (3.1%)	0 (0%)	
Type 2 diabetes *	2 (3%)	0 (0%)	0 (0%)	>0.9	2 (2.4%)	0 (0%)	0 (0%)	>0.9
Gestational diabetes *	15 (22.7%)	5 (19.2%)	1 (7.1%)	0.43	15 (18.1%)	8 (25%)	1 (5.9%)	0.27
Hypertension *	6 (9.1%)	3 (11.5%)	2 (14.3%)	0.72	5 (6%)	3 (9.4%)	2 (11.8%)	0.57
Preeclampsia *	3 (4.5%)	1 (3.8%)	1 (7.1%)	0.81	4 (4.8%)	2 (6.2%)	2 (11.8%)	0.35
Maternal metabolic condition *				0.37				0.23
BMI ≤ 25 and no metabolic conditions	25 (37.9%)	8 (30.8%)	7 (50.0%)		34 (41.0%)	8 (25.0%)	7 (41.2%)	
25 < BMI < 30 and no metabolic conditions	14 (21.2%)	6 (23.1%)	5 (35.7%)		16 (19.3%)	7 (21.9%)	5 (29.4%)	
BMI ≥ 30 and no other metabolic conditions	8 (12.1%)	4 (15.4%)	0 (0%)		14 (16.9%)	5 (15.6%)	2 (11.8%)	
Any hypertensive disorder (without any diabetes) at any BMI	2 (3.0%)	3 (11.5%)	1 (7.1%)		2 (2.4%)	4 (12.5%)	2 (11.8%)	
Diabetes at any BMI	17 (25.8%)	5 (19.2%)	1 (7.1%)		17 (20.5%)	8 (25.0%)	1 (5.9%)	

**Table 2:** Estimates of the total and direct effects of a 1 quartile increase of each phthalate metabolite in the mixture of each placental metabolite. The estimate and 95% confidence interval (CI) for each placental metabolite were bootstrapped independently using quantile g-computation models. The total effect was adjusted for birth year, homeownership, maternal education, and maternal race/ethnicity. The direct effect models were adjusted for birth year, delivery mode, gestational age at birth, homeownership, maternal education, maternal metabolic condition, and maternal race/ethnicity. All *p*-values were adjusted for false discovery rate (FDR) and both the original and FDR *p*-values are reported.

	Total Effect			Direct Effect		
Metabolite	Estimate (95% CI)	*p*	FDR *p*	Estimate (95% CI)	*p*	FDR *p*
1,3-Dihydroxyacetone	0.0034 (−0.1237, 0.1306)	0.9579	0.9579	−0.0019 (−0.1452, 0.1415)	0.9795	0.9983
2-Hydroxybutyrate	−0.0804 (−0.1378, −0.0230)	0.0071	0.0896	−0.0925 (−0.1562, −0.0287)	0.0053	0.0907
3-Hydroxybutyrate	−0.0600 (−0.1955, 0.0754)	0.3868	0.8106	−0.0655 (−0.2129, 0.0820)	0.3860	0.9046
4-Aminobutyrate	0.0081 (−0.0747, 0.0910)	0.8476	0.9579	0.0223 (−0.0632, 0.1079)	0.6103	0.9679
4-Hydroxybutyrate	−0.0103 (−0.1715, 0.1508)	0.9002	0.9579	−0.0166 (−0.1975, 0.1643)	0.8574	0.9781
Acetate	−0.0231 (−0.1363, 0.0901)	0.6899	0.9292	−0.0240 (−0.1405, 0.0925)	0.6871	0.9764
Alanine	−0.0146 (−0.0659, 0.0367)	0.5780	0.9292	−0.0122 (−0.0669, 0.0425)	0.6632	0.9679
Arginine	−0.0250 (−0.0814, 0.0313)	0.3853	0.8106	−0.0285 (−0.0954, 0.0384)	0.4058	0.9046
Asparagine	−0.0175 (−0.0706, 0.0356)	0.5192	0.8869	−0.0156 (−0.0729, 0.0416)	0.5935	0.9679
Aspartate	−0.0323 (−0.0800, 0.0153)	0.1863	0.6288	−0.0372 (−0.0891, 0.0147)	0.1634	0.5882
Betaine	−0.0473 (−0.0983, 0.0036)	0.0714	0.3613	−0.0445 (−0.1051, 0.0162)	0.1537	0.5882
Carnitine	−0.0738 (−0.1207, −0.0270)	0.0025	0.0783	−0.0785 (−0.1306, −0.0265)	0.0038	0.0907
Choline	−0.0231 (−0.0756, 0.0294)	0.3903	0.8106	−0.0237 (−0.0791, 0.0317)	0.4040	0.9046
Creatine	−0.0132 (−0.0538, 0.0274)	0.5256	0.8869	−0.0047 (−0.0546, 0.0452)	0.8537	0.9781
Cystine	0.0588 (−0.0470, 0.1646)	0.2786	0.8106	0.0647 (−0.0471, 0.1765)	0.2593	0.7779
Ethanolamine	−0.0173 (−0.0679, 0.0333)	0.5039	0.8869	−0.0214 (−0.0773, 0.0344)	0.4542	0.9084
Formate	−0.0382 (−0.1018, 0.0255)	0.2425	0.7703	−0.0336 (−0.0955, 0.0283)	0.2894	0.8225
Fumarate	−0.0628 (−0.1519, 0.0263)	0.1697	0.6185	−0.0622 (−0.1668, 0.0424)	0.2463	0.7779
Glucitol	−0.0912 (−0.1498, −0.0326)	0.0029	0.0783	−0.0956 (−0.1596, −0.0315)	0.0042	0.0907
Glutamate	−0.0160 (−0.0608, 0.0288)	0.4855	0.8869	−0.0191 (−0.0684, 0.0302)	0.4486	0.9084
Glutamine	−0.0185 (−0.0561, 0.0191)	0.3364	0.8106	−0.0257 (−0.0637, 0.0124)	0.1893	0.6389
Glutathione	−0.1151 (−0.2481, 0.0180)	0.0928	0.4176	−0.1437 (−0.2729, −0.0144)	0.0315	0.2430
Glycerol	−0.0149 (−0.0791, 0.0493)	0.6503	0.9292	−0.0160 (−0.0837, 0.0516)	0.6432	0.9679
Glycine	−0.0105 (−0.0579, 0.0369)	0.6655	0.9292	−0.0045 (−0.0585, 0.0494)	0.8694	0.9781
Hypoxanthine	−0.0167 (−0.0657, 0.0324)	0.5070	0.8869	−0.0173 (−0.0712, 0.0366)	0.5310	0.9679
Inosine	−0.0733 (−0.1778, 0.0312)	0.1718	0.6185	−0.0995 (−0.2103, 0.0112)	0.0810	0.3365
Isoleucine	−0.0099 (−0.0652, 0.0454)	0.7261	0.9294	−0.0032 (−0.0637, 0.0572)	0.9167	0.9900
Kynurenine	−0.0718 (−0.1461, 0.0025)	0.0608	0.3613	−0.0776 (−0.1594, 0.0042)	0.0656	0.2952
Lactate	0.0089 (−0.0317, 0.0495)	0.6687	0.9292	0.0082 (−0.0360, 0.0525)	0.7164	0.9781
Leucine	−0.0118 (−0.0693, 0.0458)	0.6894	0.9292	−0.0037 (−0.0666, 0.0591)	0.9076	0.9900
Lysine	−0.0307 (−0.0935, 0.0320)	0.3395	0.8106	−0.0192 (−0.0866, 0.0482)	0.5779	0.9679
Methionine	0.0147 (−0.0464, 0.0758)	0.6379	0.9292	0.0202 (−0.0459, 0.0864)	0.5497	0.9679
*myo*-Inositol	0.0223 (−0.0222, 0.0668)	0.3286	0.8106	0.0176 (−0.0357, 0.0709)	0.5187	0.9679
*N*-Acetylneuraminate	−0.0844 (−0.1460, −0.0228)	0.0083	0.0896	−0.0901 (−0.1559, −0.0244)	0.0084	0.0907
NAD^+^	−0.1044 (−0.2397, 0.0309)	0.1333	0.5537	−0.1315 (−0.2700, 0.0070)	0.0654	0.2952
Niacinamide	0.0255 (−0.2252, 0.2762)	0.8422	0.9579	−0.0034 (−0.2684, 0.2616)	0.9798	0.9983
*O*-Acetylcarnitine	−0.0665 (−0.1150, −0.0181)	0.0082	0.0896	−0.0733 (−0.1256, −0.0209)	0.0071	0.0907
*O*-Phosphocholine	−0.1516 (−0.2954, −0.0077)	0.0412	0.3178	−0.1647 (−0.3153, −0.0140)	0.0344	0.2430
*O*-Phosphoethanolamine	−0.1191 (−0.2315, −0.0067)	0.0401	0.3178	−0.1212 (−0.2405, −0.0019)	0.0490	0.2940
Ornithine	−0.0055 (−0.1014, 0.0903)	0.9098	0.9579	0.0129 (−0.0879, 0.1137)	0.8024	0.9781
Pantothenate	−0.0384 (−0.1141, 0.0373)	0.3226	0.8106	−0.0406 (−0.1219, 0.0407)	0.3297	0.8902
Phenylalanine	−0.0141 (−0.0703, 0.0421)	0.6232	0.9292	−0.0085 (−0.0702, 0.0532)	0.7878	0.9781
Proline	0.0019 (−0.0495, 0.0533)	0.9429	0.9579	0.0078 (−0.0470, 0.0627)	0.7803	0.9781
Pyroglutamate	−0.0272 (−0.0888, 0.0344)	0.3890	0.8106	−0.0281 (−0.0961, 0.0398)	0.4188	0.9046
Serine	−0.0044 (−0.0643, 0.0555)	0.8862	0.9579	0.0001 (−0.0665, 0.0667)	0.9983	0.9983
*sn*-Glycero−3-phosphocholine	−0.0390 (−0.1353, 0.0574)	0.4295	0.8590	−0.0388 (−0.1299, 0.0523)	0.4054	0.9046
Succinate	−0.0096 (−0.1030, 0.0838)	0.8403	0.9579	−0.0243 (−0.1149, 0.0662)	0.5995	0.9679
Taurine	−0.0366 (−0.0761, 0.0028)	0.0714	0.3613	−0.0406 (−0.0819, 0.0008)	0.0570	0.2952
Threonine	0.0048 (−0.0500, 0.0596)	0.8635	0.9579	0.0103 (−0.0495, 0.0701)	0.7363	0.9781
Tryptophan	0.0877 (−0.0075, 0.1829)	0.0736	0.3613	0.1052 (0.0081, 0.2022)	0.0360	0.2430
Tyrosine	0.0027 (−0.0511, 0.0565)	0.9225	0.9579	0.0080 (−0.0508, 0.0668)	0.7901	0.9781
Uracil	0.0090 (−0.0656, 0.0835)	0.8141	0.9579	0.0070 (−0.0744, 0.0884)	0.8671	0.9781
Uridine	−0.0154 (−0.0953, 0.0644)	0.7055	0.9292	−0.0199 (−0.1088, 0.0690)	0.6619	0.9679
Valine	−0.0087 (−0.0597, 0.0424)	0.7401	0.9294	−0.0013 (−0.0575, 0.0549)	0.9650	0.9983

**Table 3:** Associations between placenta metabolites and birth year. In samples with a molar sum of urinary DEHP metabolites below the median (*n* = 66), we conducted multiple linear regressions to measure the association between each log_10_-transformed placenta metabolite concentration and birth year after adjustment for gestational age at delivery, maternal metabolic condition, homeownership status, maternal race/ethnicity, and maternal education. We conducted permutation testing under the reduced model using 4999 permutations for each metabolite and corrected for false discovery rate (FDR). Only metabolites with FDR *p*-values < 0.1 are reported.

Metabolite	Estimate (95% CI)	*r^2^*	*p*	FDR *p*
Arginine	−0.0502 (−0.0965, −0.0038)	0.0681	0.0344	0.0978
Creatine	0.0405 (0.0040, 0.0771)	0.0712	0.0338	0.0978
Cystine	−0.1050 (−0.1792, −0.0308)	0.1110	0.0064	0.0432
Glutathione	0.1238 (0.0390, 0.2087)	0.1172	0.0052	0.0401
Inosine	0.1328 (0.0678, 0.1978)	0.2063	0.0000	0.0000
Isoleucine	−0.0407 (−0.0765, −0.0049)	0.0745	0.0278	0.0945
Leucine	−0.0478 (−0.0842, −0.0114)	0.0972	0.0122	0.0549
Lysine	−0.0483 (−0.0912, −0.0054)	0.0734	0.0280	0.0945
Methionine	−0.0539 (−0.0941, −0.0137)	0.1007	0.0074	0.0444
NAD^+^	0.2018 (0.1186, 0.2849)	0.2686	0.0000	0.0000
*O*-Phosphocholine	0.1244 (0.0402, 0.2086)	0.1198	0.0040	0.0360
*O*-Phosphoethanolamine	0.0935 (0.0324, 0.1545)	0.1275	0.0038	0.0360
Ornithine	−0.0536 (−0.1026, −0.0045)	0.0693	0.0320	0.0978
Phenylalanine	−0.0461 (−0.0815, −0.0108)	0.0960	0.0110	0.0549
Pyroglutamate	−0.0974 (−0.1457, −0.0490)	0.2018	0.0000	0.0000
Serine	−0.0527 (−0.0933, −0.0121)	0.0951	0.0116	0.0549
Tryptophan	−0.0934 (−0.1659, −0.0208)	0.0937	0.0138	0.0573
Tyrosine	−0.0420 (−0.0773, −0.0067)	0.0812	0.0218	0.0841
Uracil	−0.0802 (−0.1318, −0.0286)	0.1309	0.0028	0.0360

**Table 4:** The permutational multivariate analysis of variance (PERMANOVA) to assess the association between the placenta metabolome and neurodevelopmental outcome stratified by the child of interest’s sex. Placental metabolites were log_10_-transformed. The marginal effects of terms were tested under 9999 permutations.

	All Children				Females				Males			
Term	df	*F*	*R* ^2^	*p*	df	*F*	*R* ^2^	*p*	df	*F*	*R* ^2^	*p*
Birth year	1	11.22	0.0777	0.0001	1	4.19	0.0713	0.0013	1	7.12	0.0852	0.0001
Delivery mode	1	1.33	0.0092	0.2064	1	0.68	0.0116	0.7105	1	1.23	0.0147	0.2595
Gestational age at delivery, weeks	1	1.15	0.0079	0.2895	1	0.95	0.0162	0.4333	1	1.11	0.0132	0.3224
Homeownership	1	0.56	0.0039	0.8152	1	1.13	0.0192	0.3087	1	1.59	0.0190	0.1361
Maternal age, years	1	0.74	0.0051	0.6227	1	0.91	0.0156	0.4727	1	0.47	0.0057	0.8711
Maternal race/ethnicity	1	0.86	0.0059	0.5091	1	0.57	0.0097	0.8241	1	0.9	0.0108	0.4689
Maternal metabolic condition	4	0.6	0.0167	0.9596	4	0.85	0.0578	0.6787	4	0.53	0.0251	0.9843
Birth weight, grams	1	0.38	0.0026	0.9579	1	0.8	0.0136	0.5581	1	0.63	0.0076	0.7155
Prenatal vitamin use in the first month of pregnancy	1	1.81	0.0126	0.0793	1	0.64	0.0110	0.7473	1	2.34	0.0280	0.0337
Neurodevelopmental outcome	2	1.45	0.0201	0.127	2	1	0.0342	0.4164	2	1.84	0.0440	0.0451
Residual	117		0.8099		41		0.6980		61		0.7296	
Total	131		1.0000		55		1.0000		75		1.0000	

## Data Availability

The data presented in this study are available on request from the corresponding author. The data are not publicly available due to the need to protect participant privacy.

## Supplementary Materials

The following supporting information can be downloaded at: https://www.mdpi.com/article/10.3390/metabo12090829/s1, Table S1: Distribution of phthalate metabolite concentrations, Table S2: Estimates of the total and direct effects of each phthalate metabolite on each serum metabolite, Table S3: Estimates of the total and direct effects the phthalate mixture on each serum metabolite, Table S4: Associations between the serum metabolome and neurodevelopmental outcome stratified by sex, Table S5: Associations between serum metabolites and neurodevelopmental outcome, Table S6: Estimates of the total and direct effects of each phthalate metabolite on each placenta metabolite, Table S7: Associations between placenta metabolites and birth year, Table S8: Associations between placenta metabolites and neurodevelopmental outcome overall and stratified by sex, Figure S1: Correlation heatmap between placental and serum metabolites, Figure S2: Directed acyclic graphs (DAGs) for the association between phthalate exposure, serum metabolome, and neurodevelopmental outcome, Figure S3: Directed acyclic graph (DAG) for the association between phthalate exposure, placenta metabolome, and neurodevelopmental outcome.
