# Supplementary material for: Maternal Serum and Placental Metabolomes in Association with Prenatal Phthalate Exposure and Neurodevelopmental Outcomes in the MARBLES Cohort
Source: Metabolites. 2022 Sep 2;12(9):829. doi: 10.3390/metabo12090829 (PMC9500898; doi:10.3390/metabo12090829)
Supplement: Supplementary file 1 [file metabolites-12-00829-s001.zip › metabolites-1887522-supplementary.pdf]

Supplementary Materials: The maternal serum and placental metabolomes in association with prenatal phthalate exposure and neurodevelopmental outcomes in the MARBLES cohort

AUTHORS

Mariana Parenti[1], Rebecca J. Schmidt[2,3], Sally Ozonoff[3,4], Hyeong-Moo Shin[5], Daniel J. Tancredi[6], Paula Krakowiak[2], Irva Hertz-Picciotto[2,3], Cheryl K. Walker[3,7], and Carolyn M. Slupsky[1,8]

[1] Department of Nutrition, University of California, Davis, 95616, CA, USA

[2] Department of Public Health Sciences, University of California, Davis, 95616, CA, USA

[3] Medical Investigation of Neurodevelopmental Disorders (MIND) Institute, University of California, Davis, 95616, CA, USA

[4] Department of Psychiatry and Behavioral Sciences, University of California, Davis, 95616, CA, USA

[5] Department of Environmental Science, Baylor University, Waco, 76798, TX, USA

[6] Department of Pediatrics, School of Medicine, University of California, Davis, 95616, CA, USA

[7] Department of Obstetrics & Gynecology, School of Medicine, University of California, Davis, 95616, CA, USA

[8] Department of Food Science and Technology, University of California, Davis, 95616, CA, USA

FIGURES AND TABLES

Table S1. Distribution of phthalate metabolite concentrations

Table S2. Estimates of the total and direct effects of each phthalate metabolite on each serum metabolite

Table S3. Estimates of the total and direct effects the phthalate mixture on each serum metabolite

Table S4. Associations between the serum metabolome and neurodevelopmental outcome stratified by sex

Table S5. Associations between serum metabolites and neurodevelopmental outcome

Table S6. Estimates of the total and direct effects of each phthalate metabolite on each placenta metabolite

Table S7. Associations between placenta metabolites and birth year

Table S8. Associations between placenta metabolites and neurodevelopmental outcome overall and stratified by sex

Figure S1. Correlation heatmap between placental and serum metabolites

Figure S2. Directed acyclic graphs (DAGs) for the association between phthalate exposure, serum metabolome, and neurodevelopmental outcome

Figure S3. Directed acyclic graph (DAG) for the association between phthalate exposure, placenta metabolome, and neurodevelopmental outcome

Table S1: Distribution of urinary phthalate metabolite concentrations (µg/L) and the molar sum of DEHP metabolites (∑DEHP = MEHP + MEHHP + MEOHP + MECPP, µmol/L). LOD = Limit of detection (µg/L).

		Serum				Placenta			
			Percentiles				Percentiles		
	**LOD**	**% Detected**	**5**	**50**	**95**	**% Detected**	**5**	**50**	**95**
MEP	1.2	100	6.5	24.4	122.5	100	6.6	28.4	277.3
MiBP	0.8	100	2.7	7.6	26.8	100	3.2	7.8	27.5
MHiBP	0.4	100	1.0	2.7	8.7	100	1.0	2.7	8.4
MBP	0.4	100	5.3	12.1	32.4	100	5.6	12.6	34.4
MHBP	0.4	94	0.4	1.1	2.7	96	0.5	1.1	2.7
MBzP	0.3	100	1.6	6.8	34.1	100	1.9	6.7	41.0
MEHP	0.8	89	<LOD	2.9	19.1	93	<LOD	3.3	25.5
MEHHP	0.4	100	4.0	11.6	73.0	100	4.4	13.2	110.4
MEOHP	0.2	100	3.2	8.6	54.4	100	3.2	10.7	70.1
MECPP	0.4	100	7.2	19.5	98.8	100	8.1	25.0	137.4
MCPP	0.4	98	0.7	2.3	13.5	98	0.8	2.2	13.6
MNP	0.9	76	<LOD	1.5	11.1	79	<LOD	1.5	7.5
MCOP	0.3	100	5.2	21.0	147.2	100	4.9	21.1	118.8
MCNP	0.2	100	1.2	3.4	19.6	100	1.1	3.4	18.7
∑DEHP			0.1	0.1	0.8		0.1	0.2	1.2

Table S2: Estimates of the total and direct effects of each phthalate metabolite on each serum metabolite. The estimated effect of each phthalate metabolite on each serum metabolite was modeled using multiple linear regression and the estimated coefficients and 95% confidence intervals (CI) are reported. The total effect was adjusted for birth year, fasted time, gestational age at sample collection, homeownership, maternal education, and maternal race/ethnicity. The direct effect models were adjusted for birth year, birth year, fasted time, gestational age at serum collection, homeownership, maternal metabolic condition, and maternal race/ethnicity. All *p*-values were adjusted for false discovery rate (FDR) and both the original and FDR *p*-values are reported.

		Total Effect			Direct Effect		
**Phthalate**	**Metabolite**	**Estimate (95% CI)**	***p***	**FDR *p***	**Estimate (95% CI)**	***p***	**FDR *p***
MBP	2-Hydroxybutyrate	-0.0270 (-0.1821, 0.1282)	0.7309	0.9701	-0.0026 (-0.1600, 0.1548)	0.9738	0.9999
MBP	2-Hydroxyisobutyrate	-0.0395 (-0.1178, 0.0388)	0.3196	0.9330	-0.0291 (-0.1095, 0.0513)	0.4743	0.9999
MBP	2-Hydroxyisovalerate	-0.0260 (-0.1145, 0.0624)	0.5603	0.9556	-0.0201 (-0.1086, 0.0685)	0.6539	0.9999
MBP	2-Oxoglutarate	-0.0134 (-0.1089, 0.0822)	0.7818	0.9701	-0.0039 (-0.0985, 0.0907)	0.9348	0.9999
MBP	2-Oxoisocaproate	-0.0865 (-0.1865, 0.0135)	0.0892	0.9330	-0.0735 (-0.1758, 0.0288)	0.1573	0.9999
MBP	3-Hydroxybutyrate	-0.0813 (-0.3479, 0.1853)	0.5463	0.9556	-0.0859 (-0.3449, 0.1730)	0.5117	0.9999
MBP	3-Hydroxyisobutyrate	-0.0617 (-0.1946, 0.0713)	0.3598	0.9330	-0.0655 (-0.1888, 0.0578)	0.2945	0.9999
MBP	3-Hydroxyisovalerate	-0.0399 (-0.1184, 0.0385)	0.3150	0.9330	-0.0333 (-0.1104, 0.0437)	0.3922	0.9999
MBP	3-Methyl-2-oxo-butanoic acid	-0.0791 (-0.1554, -0.0028)	0.0423	0.9330	-0.0674 (-0.1462, 0.0115)	0.0932	0.9999
MBP	Acetate	-0.0971 (-0.2292, 0.0350)	0.1480	0.9330	-0.0895 (-0.2211, 0.0421)	0.1803	0.9999
MBP	Acetoacetate	-0.0657 (-0.2626, 0.1311)	0.5092	0.9556	-0.0657 (-0.2620, 0.1307)	0.5085	0.9999
MBP	Acetone	-0.0227 (-0.1680, 0.1225)	0.7567	0.9701	-0.0245 (-0.1660, 0.1171)	0.7323	0.9999
MBP	Alanine	-0.0330 (-0.1021, 0.0361)	0.3458	0.9330	-0.0378 (-0.1067, 0.0310)	0.2776	0.9999
MBP	Arginine	-0.0678 (-0.1681, 0.0326)	0.1832	0.9330	-0.0655 (-0.1668, 0.0358)	0.2026	0.9999
MBP	Asparagine	-0.0396 (-0.1361, 0.0570)	0.4182	0.9462	-0.0353 (-0.1283, 0.0576)	0.4522	0.9999
MBP	Aspartate	-0.0132 (-0.1579, 0.1314)	0.8562	0.9701	-0.0330 (-0.1718, 0.1058)	0.6380	0.9999
MBP	Betaine	-0.0160 (-0.0949, 0.0628)	0.6871	0.9701	-0.0012 (-0.0829, 0.0805)	0.9767	0.9999
MBP	Carnitine	-0.0363 (-0.1169, 0.0444)	0.3747	0.9330	-0.0328 (-0.1142, 0.0486)	0.4255	0.9999
MBP	Choline	-0.0383 (-0.1282, 0.0517)	0.4009	0.9389	-0.0294 (-0.1192, 0.0603)	0.5165	0.9999
MBP	Citrate	-0.0004 (-0.0882, 0.0874)	0.9929	0.9964	0.0144 (-0.0702, 0.0991)	0.7357	0.9999
MBP	Creatine	-0.0085 (-0.1507, 0.1336)	0.9055	0.9782	-0.0073 (-0.1482, 0.1336)	0.9181	0.9999
MBP	Creatinine	-0.0205 (-0.0887, 0.0478)	0.5533	0.9556	-0.0151 (-0.0838, 0.0536)	0.6634	0.9999
MBP	Cystine	-0.0039 (-0.1260, 0.1181)	0.9490	0.9901	0.0137 (-0.1111, 0.1384)	0.8283	0.9999
MBP	Dimethyl sulfone	-0.0582 (-0.2092, 0.0928)	0.4460	0.9556	-0.0605 (-0.2034, 0.0825)	0.4033	0.9999
MBP	Formate	-0.0761 (-0.1567, 0.0044)	0.0636	0.9330	-0.0763 (-0.1562, 0.0037)	0.0612	0.9999
MBP	Glucose	-0.0366 (-0.1221, 0.0489)	0.3974	0.9389	-0.0346 (-0.1200, 0.0508)	0.4233	0.9999
MBP	Glutamate	-0.0112 (-0.1538, 0.1314)	0.8763	0.9702	-0.0186 (-0.1598, 0.1227)	0.7946	0.9999
MBP	Glutamine	-0.0668 (-0.1231, -0.0105)	0.0206	0.9330	-0.0662 (-0.1230, -0.0093)	0.0230	0.9999
MBP	Glycine	-0.0504 (-0.1376, 0.0368)	0.2541	0.9330	-0.0508 (-0.1379, 0.0364)	0.2506	0.9999
MBP	Isoleucine	-0.0613 (-0.1732, 0.0506)	0.2799	0.9330	-0.0662 (-0.1775, 0.0450)	0.2402	0.9999
MBP	Lactate	0.0368 (-0.0624, 0.1359)	0.4636	0.9556	0.0324 (-0.0658, 0.1305)	0.5141	0.9999
MBP	Leucine	-0.0640 (-0.1595, 0.0315)	0.1867	0.9330	-0.0691 (-0.1630, 0.0247)	0.1469	0.9999
MBP	Lysine	-0.0257 (-0.1229, 0.0716)	0.6015	0.9623	-0.0323 (-0.1287, 0.0642)	0.5082	0.9999
MBP	Methanol	-0.2814 (-0.4931, -0.0697)	0.0097	0.9330	-0.2764 (-0.4869, -0.0660)	0.0106	0.9999
MBP	Methionine	-0.0547 (-0.1478, 0.0383)	0.2460	0.9330	-0.0592 (-0.1510, 0.0325)	0.2029	0.9999
MBP	myo-Inositol	-0.0232 (-0.1074, 0.0611)	0.5862	0.9556	-0.0178 (-0.1033, 0.0677)	0.6803	0.9999
MBP	N,N-Dimethylglycine	-0.0585 (-0.1344, 0.0174)	0.1296	0.9330	-0.0486 (-0.1258, 0.0287)	0.2152	0.9999
MBP	O-Acetylcarnitine	-0.0067 (-0.0904, 0.0769)	0.8734	0.9701	-0.0010 (-0.0833, 0.0814)	0.9812	0.9999
MBP	Ornithine	-0.0439 (-0.1668, 0.0789)	0.4797	0.9556	-0.0584 (-0.1776, 0.0609)	0.3336	0.9999
MBP	Phenylalanine	-0.0459 (-0.1269, 0.0352)	0.2641	0.9330	-0.0494 (-0.1297, 0.0310)	0.2259	0.9999
MBP	Proline	0.0141 (-0.0834, 0.1115)	0.7754	0.9701	0.0107 (-0.0867, 0.1080)	0.8282	0.9999
MBP	Pyruvate	0.0151 (-0.1615, 0.1918)	0.8653	0.9701	0.0003 (-0.1722, 0.1728)	0.9971	0.9999
MBP	Serine	-0.0425 (-0.1294, 0.0444)	0.3342	0.9330	-0.0502 (-0.1340, 0.0335)	0.2368	0.9999
MBP	Taurine	0.0888 (-0.0672, 0.2448)	0.2614	0.9330	0.0708 (-0.0791, 0.2207)	0.3509	0.9999
MBP	Threonine	-0.0401 (-0.1773, 0.0972)	0.5639	0.9556	-0.0373 (-0.1754, 0.1008)	0.5934	0.9999
MBP	Tyrosine	-0.0439 (-0.1409, 0.0530)	0.3708	0.9330	-0.0471 (-0.1419, 0.0477)	0.3262	0.9999
MBP	Urea	-0.0231 (-0.1396, 0.0934)	0.6948	0.9701	-0.0131 (-0.1251, 0.0989)	0.8163	0.9999
MBP	Valine	-0.0416 (-0.1304, 0.0473)	0.3558	0.9330	-0.0446 (-0.1322, 0.0430)	0.3146	0.9999
MBzP	2-Hydroxybutyrate	0.0178 (-0.0846, 0.1201)	0.7311	0.9701	-0.0153 (-0.1206, 0.0900)	0.7736	0.9999
MBzP	2-Hydroxyisobutyrate	0.0331 (-0.0183, 0.0846)	0.2045	0.9330	0.0198 (-0.0340, 0.0736)	0.4667	0.9999
MBzP	2-Hydroxyisovalerate	0.0268 (-0.0315, 0.0850)	0.3639	0.9330	0.0166 (-0.0427, 0.0758)	0.5795	0.9999

		Total Effect			Direct Effect		
**Phthalate**	**Metabolite**	**Estimate (95% CI)**	***p***	**FDR *p***	**Estimate (95% CI)**	***p***	**FDR *p***
MBzP	2-Oxoglutarate	0.0143 (-0.0487, 0.0773)	0.6533	0.9701	-0.0033 (-0.0666, 0.0600)	0.9181	0.9999
MBzP	2-Oxoisocaproate	0.0112 (-0.0557, 0.0781)	0.7408	0.9701	-0.0068 (-0.0760, 0.0624)	0.8453	0.9999
MBzP	3-Hydroxybutyrate	0.0457 (-0.1302, 0.2217)	0.6072	0.9623	-0.0127 (-0.1864, 0.1610)	0.8847	0.9999
MBzP	3-Hydroxyisobutyrate	-0.0324 (-0.1202, 0.0555)	0.4664	0.9556	-0.0474 (-0.1299, 0.0351)	0.2568	0.9999
MBzP	3-Hydroxyisovalerate	-0.0031 (-0.0551, 0.0490)	0.9075	0.9785	-0.0179 (-0.0695, 0.0337)	0.4925	0.9999
MBzP	3-Methyl-2-oxo-butanoic acid	-0.0101 (-0.0615, 0.0412)	0.6966	0.9701	-0.0208 (-0.0742, 0.0326)	0.4421	0.9999
MBzP	Acetate	-0.0864 (-0.1728, -0.0001)	0.0498	0.9330	-0.0644 (-0.1523, 0.0236)	0.1496	0.9999
MBzP	Acetoacetate	0.0217 (-0.1084, 0.1518)	0.7412	0.9701	-0.0072 (-0.1389, 0.1246)	0.9144	0.9999
MBzP	Acetone	-0.0169 (-0.1127, 0.0789)	0.7277	0.9701	-0.0353 (-0.1299, 0.0592)	0.4601	0.9999
MBzP	Alanine	-0.0146 (-0.0603, 0.0312)	0.5290	0.9556	-0.0037 (-0.0500, 0.0426)	0.8741	0.9999
MBzP	Arginine	0.0136 (-0.0531, 0.0803)	0.6868	0.9701	0.0019 (-0.0665, 0.0702)	0.9573	0.9999
MBzP	Asparagine	-0.0398 (-0.1032, 0.0236)	0.2161	0.9330	-0.0265 (-0.0887, 0.0356)	0.3991	0.9999
MBzP	Aspartate	-0.0149 (-0.1103, 0.0805)	0.7573	0.9701	-0.0289 (-0.1217, 0.0640)	0.5383	0.9999
MBzP	Betaine	-0.0088 (-0.0608, 0.0432)	0.7372	0.9701	-0.0250 (-0.0795, 0.0295)	0.3646	0.9999
MBzP	Carnitine	-0.0092 (-0.0626, 0.0442)	0.7326	0.9701	-0.0159 (-0.0705, 0.0387)	0.5643	0.9999
MBzP	Choline	-0.0011 (-0.0607, 0.0585)	0.9708	0.9924	-0.0196 (-0.0797, 0.0405)	0.5185	0.9999
MBzP	Citrate	0.0047 (-0.0532, 0.0626)	0.8725	0.9701	-0.0103 (-0.0670, 0.0463)	0.7179	0.9999
MBzP	Creatine	0.0122 (-0.0816, 0.1059)	0.7970	0.9701	0.0060 (-0.0883, 0.1003)	0.9000	0.9999
MBzP	Creatinine	-0.0312 (-0.0759, 0.0134)	0.1685	0.9330	-0.0336 (-0.0792, 0.0119)	0.1457	0.9999
MBzP	Cystine	-0.0259 (-0.1062, 0.0545)	0.5246	0.9556	-0.0329 (-0.1161, 0.0504)	0.4352	0.9999
MBzP	Dimethyl sulfone	-0.0765 (-0.1753, 0.0222)	0.1270	0.9330	-0.0487 (-0.1442, 0.0468)	0.3139	0.9999
MBzP	Formate	-0.0469 (-0.1001, 0.0064)	0.0838	0.9330	-0.0392 (-0.0931, 0.0147)	0.1522	0.9999
MBzP	Glucose	-0.0472 (-0.1031, 0.0086)	0.0963	0.9330	-0.0422 (-0.0989, 0.0145)	0.1427	0.9999
MBzP	Glutamate	-0.0135 (-0.1075, 0.0806)	0.7768	0.9701	-0.0254 (-0.1199, 0.0690)	0.5941	0.9999
MBzP	Glutamine	-0.0143 (-0.0524, 0.0238)	0.4576	0.9556	-0.0158 (-0.0547, 0.0232)	0.4236	0.9999
MBzP	Glycine	-0.0409 (-0.0983, 0.0164)	0.1596	0.9330	-0.0393 (-0.0975, 0.0189)	0.1832	0.9999
MBzP	Isoleucine	0.0056 (-0.0687, 0.0798)	0.8824	0.9737	-0.0027 (-0.0777, 0.0723)	0.9425	0.9999
MBzP	Lactate	-0.0053 (-0.0709, 0.0602)	0.8722	0.9701	0.0003 (-0.0656, 0.0661)	0.9934	0.9999
MBzP	Leucine	0.0001 (-0.0635, 0.0636)	0.9985	0.9985	-0.0112 (-0.0746, 0.0523)	0.7280	0.9999
MBzP	Lysine	-0.0021 (-0.0663, 0.0622)	0.9487	0.9901	-0.0173 (-0.0819, 0.0473)	0.5972	0.9999
MBzP	Methanol	-0.1938 (-0.3330, -0.0546)	0.0069	0.9330	-0.1871 (-0.3278, -0.0464)	0.0097	0.9999
MBzP	Methionine	-0.0071 (-0.0688, 0.0547)	0.8213	0.9701	-0.0030 (-0.0649, 0.0589)	0.9234	0.9999
MBzP	myo-Inositol	-0.0491 (-0.1039, 0.0057)	0.0785	0.9330	-0.0482 (-0.1047, 0.0082)	0.0932	0.9999
MBzP	N,N-Dimethylglycine	-0.0123 (-0.0629, 0.0383)	0.6309	0.9677	-0.0219 (-0.0739, 0.0300)	0.4040	0.9999
MBzP	O-Acetylcarnitine	0.0018 (-0.0534, 0.0570)	0.9493	0.9901	-0.0218 (-0.0767, 0.0332)	0.4338	0.9999
MBzP	Ornithine	-0.0576 (-0.1381, 0.0228)	0.1584	0.9330	-0.0509 (-0.1304, 0.0287)	0.2072	0.9999
MBzP	Phenylalanine	-0.0153 (-0.0690, 0.0385)	0.5742	0.9556	-0.0186 (-0.0727, 0.0355)	0.4956	0.9999
MBzP	Proline	0.0356 (-0.0284, 0.0995)	0.2725	0.9330	0.0339 (-0.0309, 0.0987)	0.3017	0.9999
MBzP	Pyruvate	0.0078 (-0.1088, 0.1244)	0.8946	0.9770	0.0522 (-0.0628, 0.1672)	0.3696	0.9999
MBzP	Serine	-0.0204 (-0.0778, 0.0371)	0.4835	0.9556	-0.0209 (-0.0772, 0.0354)	0.4623	0.9999
MBzP	Taurine	-0.0037 (-0.1073, 0.0998)	0.9431	0.9901	-0.0156 (-0.1164, 0.0851)	0.7586	0.9999
MBzP	Threonine	0.0025 (-0.0883, 0.0932)	0.9572	0.9901	0.0033 (-0.0893, 0.0959)	0.9435	0.9999
MBzP	Tyrosine	-0.0101 (-0.0743, 0.0541)	0.7553	0.9701	0.0004 (-0.0634, 0.0641)	0.9907	0.9999
MBzP	Urea	-0.0048 (-0.0817, 0.0721)	0.9016	0.9782	-0.0020 (-0.0770, 0.0730)	0.9583	0.9999
MBzP	Valine	-0.0031 (-0.0620, 0.0558)	0.9175	0.9818	-0.0074 (-0.0663, 0.0516)	0.8042	0.9999
MCNP	2-Hydroxybutyrate	0.0880 (-0.0177, 0.1937)	0.1019	0.9330	0.1257 (0.0147, 0.2366)	0.0269	0.9999
MCNP	2-Hydroxyisobutyrate	-0.0103 (-0.0646, 0.0440)	0.7075	0.9701	-0.0026 (-0.0609, 0.0557)	0.9292	0.9999
MCNP	2-Hydroxyisovalerate	0.0105 (-0.0507, 0.0716)	0.7350	0.9701	0.0084 (-0.0557, 0.0725)	0.7956	0.9999
MCNP	2-Oxoglutarate	-0.0358 (-0.1014, 0.0298)	0.2815	0.9330	-0.0315 (-0.0996, 0.0366)	0.3614	0.9999
MCNP	2-Oxoisocaproate	0.0120 (-0.0580, 0.0821)	0.7340	0.9701	0.0230 (-0.0517, 0.0976)	0.5431	0.9999
MCNP	3-Hydroxybutyrate	-0.0227 (-0.2071, 0.1616)	0.8072	0.9701	0.0136 (-0.1741, 0.2013)	0.8859	0.9999
MCNP	3-Hydroxyisobutyrate	0.0347 (-0.0573, 0.1266)	0.4563	0.9556	0.0726 (-0.0159, 0.1611)	0.1069	0.9999
MCNP	3-Hydroxyisovalerate	0.0152 (-0.0391, 0.0696)	0.5794	0.9556	0.0098 (-0.0461, 0.0657)	0.7286	0.9999
MCNP	3-Methyl-2-oxo-butanoic acid	0.0059 (-0.0479, 0.0597)	0.8276	0.9701	0.0127 (-0.0452, 0.0705)	0.6650	0.9999
MCNP	Acetate	0.0017 (-0.0904, 0.0939)	0.9701	0.9924	0.0054 (-0.0907, 0.1015)	0.9111	0.9999
MCNP	Acetoacetate	-0.0356 (-0.1716, 0.1005)	0.6050	0.9623	-0.0132 (-0.1555, 0.1292)	0.8545	0.9999
MCNP	Acetone	0.0283 (-0.0719, 0.1284)	0.5770	0.9556	0.0439 (-0.0582, 0.1460)	0.3950	0.9999
MCNP	Alanine	0.0260 (-0.0217, 0.0737)	0.2820	0.9330	0.0360 (-0.0135, 0.0856)	0.1521	0.9999
MCNP	Arginine	0.0018 (-0.0681, 0.0717)	0.9588	0.9901	0.0292 (-0.0445, 0.1029)	0.4333	0.9999

		Total Effect			Direct Effect		
**Phthalate**	**Metabolite**	**Estimate (95% CI)**	***p***	**FDR *p***	**Estimate (95% CI)**	***p***	**FDR *p***
MCNP	Asparagine	-0.0042 (-0.0711, 0.0626)	0.8999	0.9782	0.0065 (-0.0610, 0.0739)	0.8496	0.9999
MCNP	Aspartate	-0.0894 (-0.1877, 0.0088)	0.0739	0.9330	-0.0525 (-0.1525, 0.0475)	0.2998	0.9999
MCNP	Betaine	0.0251 (-0.0292, 0.0793)	0.3615	0.9330	0.0297 (-0.0291, 0.0885)	0.3188	0.9999
MCNP	Carnitine	0.0164 (-0.0394, 0.0722)	0.5610	0.9556	0.0288 (-0.0300, 0.0876)	0.3340	0.9999
MCNP	Choline	0.0025 (-0.0599, 0.0648)	0.9370	0.9901	0.0189 (-0.0461, 0.0838)	0.5657	0.9999
MCNP	Citrate	0.0163 (-0.0443, 0.0768)	0.5950	0.9588	0.0033 (-0.0580, 0.0646)	0.9157	0.9999
MCNP	Creatine	0.0439 (-0.0539, 0.1417)	0.3750	0.9330	0.0447 (-0.0568, 0.1462)	0.3839	0.9999
MCNP	Creatinine	0.0100 (-0.0372, 0.0571)	0.6756	0.9701	0.0120 (-0.0377, 0.0617)	0.6325	0.9999
MCNP	Cystine	-0.0574 (-0.1409, 0.0261)	0.1757	0.9330	-0.0579 (-0.1474, 0.0316)	0.2019	0.9999
MCNP	Dimethyl sulfone	0.0648 (-0.0389, 0.1685)	0.2182	0.9330	0.0619 (-0.0411, 0.1649)	0.2360	0.9999
MCNP	Formate	-0.0114 (-0.0680, 0.0451)	0.6890	0.9701	-0.0056 (-0.0645, 0.0532)	0.8499	0.9999
MCNP	Glucose	0.0003 (-0.0589, 0.0596)	0.9908	0.9964	-0.0004 (-0.0624, 0.0616)	0.9899	0.9999
MCNP	Glutamate	-0.0745 (-0.1718, 0.0229)	0.1321	0.9330	-0.0517 (-0.1534, 0.0500)	0.3156	0.9999
MCNP	Glutamine	0.0117 (-0.0282, 0.0516)	0.5628	0.9556	0.0054 (-0.0368, 0.0477)	0.7988	0.9999
MCNP	Glycine	0.0168 (-0.0437, 0.0773)	0.5822	0.9556	0.0158 (-0.0476, 0.0793)	0.6211	0.9999
MCNP	Isoleucine	0.0450 (-0.0321, 0.1222)	0.2497	0.9330	0.0658 (-0.0141, 0.1458)	0.1054	0.9999
MCNP	Lactate	-0.0244 (-0.0929, 0.0440)	0.4801	0.9556	-0.0070 (-0.0781, 0.0642)	0.8462	0.9999
MCNP	Leucine	0.0242 (-0.0422, 0.0905)	0.4714	0.9556	0.0508 (-0.0171, 0.1186)	0.1408	0.9999
MCNP	Lysine	0.0417 (-0.0251, 0.1084)	0.2183	0.9330	0.0661 (-0.0025, 0.1347)	0.0589	0.9999
MCNP	Methanol	0.0233 (-0.1279, 0.1745)	0.7604	0.9701	0.0185 (-0.1390, 0.1760)	0.8161	0.9999
MCNP	Methionine	0.0378 (-0.0264, 0.1020)	0.2459	0.9330	0.0539 (-0.0121, 0.1199)	0.1084	0.9999
MCNP	myo-Inositol	-0.0210 (-0.0791, 0.0371)	0.4755	0.9556	-0.0022 (-0.0641, 0.0598)	0.9448	0.9999
MCNP	N,N-Dimethylglycine	0.0116 (-0.0414, 0.0646)	0.6659	0.9701	0.0322 (-0.0237, 0.0882)	0.2557	0.9999
MCNP	O-Acetylcarnitine	0.0247 (-0.0328, 0.0823)	0.3956	0.9389	0.0512 (-0.0075, 0.1098)	0.0865	0.9999
MCNP	Ornithine	-0.0215 (-0.1065, 0.0634)	0.6160	0.9626	-0.0045 (-0.0912, 0.0822)	0.9180	0.9999
MCNP	Phenylalanine	0.0065 (-0.0498, 0.0628)	0.8185	0.9701	0.0166 (-0.0419, 0.0751)	0.5748	0.9999
MCNP	Proline	0.0171 (-0.0502, 0.0843)	0.6159	0.9626	0.0356 (-0.0344, 0.1057)	0.3155	0.9999
MCNP	Pyruvate	-0.0753 (-0.1964, 0.0457)	0.2199	0.9330	-0.0529 (-0.1772, 0.0715)	0.4007	0.9999
MCNP	Serine	-0.0040 (-0.0643, 0.0563)	0.8956	0.9770	0.0106 (-0.0504, 0.0716)	0.7308	0.9999
MCNP	Taurine	-0.0527 (-0.1606, 0.0552)	0.3345	0.9330	-0.0131 (-0.1220, 0.0958)	0.8122	0.9999
MCNP	Threonine	-0.0356 (-0.1302, 0.0591)	0.4579	0.9556	-0.0271 (-0.1270, 0.0728)	0.5920	0.9999
MCNP	Tyrosine	0.0271 (-0.0400, 0.0941)	0.4251	0.9462	0.0470 (-0.0213, 0.1152)	0.1752	0.9999
MCNP	Urea	0.0228 (-0.0576, 0.1031)	0.5755	0.9556	0.0263 (-0.0546, 0.1071)	0.5208	0.9999
MCNP	Valine	0.0315 (-0.0299, 0.0928)	0.3111	0.9330	0.0527 (-0.0102, 0.1155)	0.0993	0.9999
MCOP	2-Hydroxybutyrate	-0.0005 (-0.0862, 0.0852)	0.9905	0.9964	0.0480 (-0.0406, 0.1365)	0.2850	0.9999
MCOP	2-Hydroxyisobutyrate	-0.0237 (-0.0669, 0.0194)	0.2779	0.9330	-0.0135 (-0.0590, 0.0320)	0.5580	0.9999
MCOP	2-Hydroxyisovalerate	-0.0255 (-0.0741, 0.0231)	0.3007	0.9330	-0.0221 (-0.0720, 0.0279)	0.3826	0.9999
MCOP	2-Oxoglutarate	-0.0050 (-0.0577, 0.0477)	0.8515	0.9701	0.0137 (-0.0397, 0.0671)	0.6121	0.9999
MCOP	2-Oxoisocaproate	-0.0513 (-0.1064, 0.0038)	0.0676	0.9330	-0.0347 (-0.0928, 0.0234)	0.2382	0.9999
MCOP	3-Hydroxybutyrate	-0.0611 (-0.2080, 0.0858)	0.4115	0.9462	-0.0038 (-0.1506, 0.1431)	0.9596	0.9999
MCOP	3-Hydroxyisobutyrate	-0.0100 (-0.0837, 0.0636)	0.7874	0.9701	0.0259 (-0.0441, 0.0959)	0.4649	0.9999
MCOP	3-Hydroxyisovalerate	-0.0058 (-0.0493, 0.0377)	0.7927	0.9701	0.0003 (-0.0435, 0.0440)	0.9908	0.9999
MCOP	3-Methyl-2-oxo-butanoic acid	-0.0321 (-0.0746, 0.0104)	0.1373	0.9330	-0.0216 (-0.0666, 0.0235)	0.3445	0.9999
MCOP	Acetate	0.0103 (-0.0633, 0.0840)	0.7812	0.9701	0.0050 (-0.0701, 0.0802)	0.8946	0.9999
MCOP	Acetoacetate	-0.0784 (-0.1862, 0.0293)	0.1517	0.9330	-0.0540 (-0.1648, 0.0568)	0.3356	0.9999
MCOP	Acetone	0.0037 (-0.0765, 0.0839)	0.9270	0.9870	0.0191 (-0.0609, 0.0992)	0.6365	0.9999
MCOP	Alanine	0.0045 (-0.0338, 0.0428)	0.8166	0.9701	0.0055 (-0.0337, 0.0447)	0.7811	0.9999
MCOP	Arginine	-0.0163 (-0.0721, 0.0395)	0.5632	0.9556	-0.0088 (-0.0666, 0.0490)	0.7633	0.9999
MCOP	Asparagine	0.0121 (-0.0413, 0.0655)	0.6544	0.9701	0.0061 (-0.0467, 0.0588)	0.8192	0.9999
MCOP	Aspartate	-0.0134 (-0.0932, 0.0664)	0.7402	0.9701	0.0094 (-0.0692, 0.0881)	0.8123	0.9999
MCOP	Betaine	-0.0094 (-0.0529, 0.0341)	0.6704	0.9701	-0.0060 (-0.0522, 0.0403)	0.7989	0.9999
MCOP	Carnitine	-0.0329 (-0.0772, 0.0113)	0.1425	0.9330	-0.0171 (-0.0632, 0.0290)	0.4639	0.9999
MCOP	Choline	-0.0137 (-0.0635, 0.0361)	0.5861	0.9556	0.0019 (-0.0490, 0.0527)	0.9424	0.9999
MCOP	Citrate	0.0259 (-0.0223, 0.0741)	0.2891	0.9330	0.0358 (-0.0116, 0.0832)	0.1369	0.9999
MCOP	Creatine	0.0020 (-0.0765, 0.0804)	0.9607	0.9901	0.0015 (-0.0782, 0.0813)	0.9697	0.9999
MCOP	Creatinine	0.0358 (-0.0013, 0.0728)	0.0582	0.9330	0.0387 (0.0006, 0.0768)	0.0465	0.9999
MCOP	Cystine	-0.0445 (-0.1112, 0.0223)	0.1896	0.9330	-0.0331 (-0.1033, 0.0372)	0.3527	0.9999
MCOP	Dimethyl sulfone	0.0594 (-0.0233, 0.1421)	0.1570	0.9330	0.0311 (-0.0498, 0.1120)	0.4474	0.9999
MCOP	Formate	-0.0142 (-0.0593, 0.0310)	0.5343	0.9556	-0.0104 (-0.0564, 0.0356)	0.6549	0.9999
MCOP	Glucose	0.0092 (-0.0381, 0.0566)	0.6998	0.9701	0.0104 (-0.0380, 0.0589)	0.6697	0.9999

		Total Effect			Direct Effect		
**Phthalate**	**Metabolite**	**Estimate (95% CI)**	***p***	**FDR *p***	**Estimate (95% CI)**	***p***	**FDR *p***
MCOP	Glutamate	-0.0285 (-0.1070, 0.0500)	0.4731	0.9556	-0.0091 (-0.0891, 0.0708)	0.8214	0.9999
MCOP	Glutamine	-0.0049 (-0.0369, 0.0270)	0.7597	0.9701	-0.0121 (-0.0451, 0.0208)	0.4663	0.9999
MCOP	Glycine	0.0324 (-0.0156, 0.0804)	0.1836	0.9330	0.0277 (-0.0216, 0.0771)	0.2677	0.9999
MCOP	Isoleucine	-0.0135 (-0.0755, 0.0486)	0.6680	0.9701	-0.0012 (-0.0646, 0.0622)	0.9692	0.9999
MCOP	Lactate	0.0177 (-0.0370, 0.0725)	0.5216	0.9556	0.0275 (-0.0279, 0.0829)	0.3265	0.9999
MCOP	Leucine	-0.0176 (-0.0707, 0.0355)	0.5124	0.9556	-0.0036 (-0.0573, 0.0501)	0.8943	0.9999
MCOP	Lysine	-0.0044 (-0.0582, 0.0493)	0.8707	0.9701	0.0076 (-0.0470, 0.0623)	0.7823	0.9999
MCOP	Methanol	-0.0341 (-0.1548, 0.0867)	0.5766	0.9556	-0.0174 (-0.1405, 0.1058)	0.7802	0.9999
MCOP	Methionine	0.0332 (-0.0180, 0.0845)	0.2014	0.9330	0.0339 (-0.0179, 0.0858)	0.1973	0.9999
MCOP	myo-Inositol	0.0084 (-0.0381, 0.0550)	0.7199	0.9701	0.0242 (-0.0239, 0.0724)	0.3204	0.9999
MCOP	N,N-Dimethylglycine	-0.0061 (-0.0484, 0.0363)	0.7774	0.9701	0.0064 (-0.0377, 0.0504)	0.7751	0.9999
MCOP	O-Acetylcarnitine	-0.0483 (-0.0934, -0.0032)	0.0363	0.9330	-0.0214 (-0.0678, 0.0250)	0.3619	0.9999
MCOP	Ornithine	0.0086 (-0.0594, 0.0766)	0.8025	0.9701	0.0156 (-0.0521, 0.0833)	0.6490	0.9999
MCOP	Phenylalanine	0.0051 (-0.0399, 0.0501)	0.8218	0.9701	0.0077 (-0.0381, 0.0535)	0.7398	0.9999
MCOP	Proline	-0.0031 (-0.0569, 0.0507)	0.9086	0.9785	0.0029 (-0.0522, 0.0579)	0.9183	0.9999
MCOP	Pyruvate	0.0275 (-0.0698, 0.1249)	0.5760	0.9556	0.0263 (-0.0711, 0.1238)	0.5930	0.9999
MCOP	Serine	0.0043 (-0.0439, 0.0524)	0.8611	0.9701	0.0075 (-0.0402, 0.0552)	0.7553	0.9999
MCOP	Taurine	0.0443 (-0.0419, 0.1305)	0.3098	0.9330	0.0671 (-0.0170, 0.1512)	0.1168	0.9999
MCOP	Threonine	-0.0047 (-0.0806, 0.0712)	0.9030	0.9782	-0.0156 (-0.0938, 0.0626)	0.6926	0.9999
MCOP	Tyrosine	0.0334 (-0.0200, 0.0867)	0.2173	0.9330	0.0363 (-0.0171, 0.0897)	0.1808	0.9999
MCOP	Urea	-0.0155 (-0.0797, 0.0488)	0.6341	0.9677	-0.0093 (-0.0727, 0.0541)	0.7717	0.9999
MCOP	Valine	-0.0126 (-0.0618, 0.0366)	0.6125	0.9626	0.0014 (-0.0485, 0.0512)	0.9564	0.9999
MCPP	2-Hydroxybutyrate	-0.0033 (-0.1271, 0.1206)	0.9584	0.9901	0.0351 (-0.0937, 0.1640)	0.5898	0.9999
MCPP	2-Hydroxyisobutyrate	-0.0226 (-0.0852, 0.0400)	0.4747	0.9556	-0.0205 (-0.0865, 0.0454)	0.5378	0.9999
MCPP	2-Hydroxyisovalerate	-0.0457 (-0.1158, 0.0243)	0.1983	0.9330	-0.0484 (-0.1204, 0.0236)	0.1855	0.9999
MCPP	2-Oxoglutarate	-0.0426 (-0.1184, 0.0331)	0.2670	0.9330	-0.0264 (-0.1038, 0.0510)	0.4998	0.9999
MCPP	2-Oxoisocaproate	-0.0506 (-0.1310, 0.0297)	0.2138	0.9330	-0.0407 (-0.1250, 0.0437)	0.3412	0.9999
MCPP	3-Hydroxybutyrate	-0.1219 (-0.3336, 0.0897)	0.2557	0.9330	-0.0598 (-0.2722, 0.1527)	0.5778	0.9999
MCPP	3-Hydroxyisobutyrate	-0.0316 (-0.1379, 0.0748)	0.5570	0.9556	0.0049 (-0.0968, 0.1067)	0.9232	0.9999
MCPP	3-Hydroxyisovalerate	-0.0103 (-0.0732, 0.0525)	0.7447	0.9701	-0.0062 (-0.0696, 0.0572)	0.8462	0.9999
MCPP	3-Methyl-2-oxo-butanoic acid	-0.0463 (-0.1078, 0.0151)	0.1378	0.9330	-0.0431 (-0.1082, 0.0219)	0.1911	0.9999
MCPP	Acetate	-0.0151 (-0.1216, 0.0914)	0.7790	0.9701	-0.0262 (-0.1350, 0.0826)	0.6336	0.9999
MCPP	Acetoacetate	-0.1400 (-0.2948, 0.0148)	0.0758	0.9330	-0.1178 (-0.2774, 0.0418)	0.1461	0.9999
MCPP	Acetone	-0.0299 (-0.1456, 0.0859)	0.6099	0.9626	-0.0197 (-0.1358, 0.0964)	0.7369	0.9999
MCPP	Alanine	0.0112 (-0.0441, 0.0666)	0.6888	0.9701	0.0165 (-0.0401, 0.0732)	0.5642	0.9999
MCPP	Arginine	-0.0230 (-0.1036, 0.0577)	0.5734	0.9556	-0.0219 (-0.1055, 0.0618)	0.6053	0.9999
MCPP	Asparagine	-0.0270 (-0.1040, 0.0501)	0.4894	0.9556	-0.0446 (-0.1205, 0.0313)	0.2464	0.9999
MCPP	Aspartate	-0.0683 (-0.1828, 0.0463)	0.2399	0.9330	-0.0406 (-0.1543, 0.0731)	0.4801	0.9999
MCPP	Betaine	-0.0319 (-0.0944, 0.0307)	0.3151	0.9330	-0.0430 (-0.1095, 0.0234)	0.2018	0.9999
MCPP	Carnitine	-0.0268 (-0.0912, 0.0376)	0.4102	0.9462	-0.0090 (-0.0760, 0.0579)	0.7897	0.9999
MCPP	Choline	-0.0504 (-0.1217, 0.0209)	0.1639	0.9330	-0.0459 (-0.1191, 0.0272)	0.2155	0.9999
MCPP	Citrate	-0.0013 (-0.0713, 0.0688)	0.9717	0.9924	0.0011 (-0.0684, 0.0705)	0.9761	0.9999
MCPP	Creatine	0.0295 (-0.0837, 0.1428)	0.6059	0.9623	0.0229 (-0.0926, 0.1383)	0.6951	0.9999
MCPP	Creatinine	0.0139 (-0.0406, 0.0683)	0.6146	0.9626	0.0111 (-0.0453, 0.0674)	0.6970	0.9999
MCPP	Cystine	-0.0942 (-0.1897, 0.0013)	0.0532	0.9330	-0.0922 (-0.1927, 0.0084)	0.0721	0.9999
MCPP	Dimethyl sulfone	0.0965 (-0.0227, 0.2157)	0.1115	0.9330	0.0592 (-0.0578, 0.1762)	0.3179	0.9999
MCPP	Formate	-0.0343 (-0.0993, 0.0307)	0.2973	0.9330	-0.0318 (-0.0982, 0.0347)	0.3449	0.9999
MCPP	Glucose	0.0214 (-0.0469, 0.0897)	0.5356	0.9556	0.0277 (-0.0423, 0.0978)	0.4335	0.9999
MCPP	Glutamate	-0.0654 (-0.1784, 0.0476)	0.2535	0.9330	-0.0425 (-0.1580, 0.0731)	0.4674	0.9999
MCPP	Glutamine	-0.0184 (-0.0644, 0.0277)	0.4302	0.9473	-0.0298 (-0.0773, 0.0177)	0.2157	0.9999
MCPP	Glycine	0.0253 (-0.0445, 0.0952)	0.4736	0.9556	0.0164 (-0.0555, 0.0883)	0.6519	0.9999
MCPP	Isoleucine	-0.0221 (-0.1118, 0.0676)	0.6255	0.9677	-0.0077 (-0.0996, 0.0842)	0.8685	0.9999
MCPP	Lactate	0.0272 (-0.0519, 0.1063)	0.4962	0.9556	0.0403 (-0.0399, 0.1206)	0.3209	0.9999
MCPP	Leucine	-0.0392 (-0.1156, 0.0373)	0.3120	0.9330	-0.0255 (-0.1032, 0.0521)	0.5151	0.9999
MCPP	Lysine	-0.0116 (-0.0893, 0.0660)	0.7673	0.9701	0.0006 (-0.0786, 0.0799)	0.9874	0.9999
MCPP	Methanol	-0.0305 (-0.2052, 0.1442)	0.7297	0.9701	-0.0137 (-0.1923, 0.1648)	0.8790	0.9999
MCPP	Methionine	0.0300 (-0.0445, 0.1045)	0.4256	0.9462	0.0282 (-0.0474, 0.1039)	0.4606	0.9999
MCPP	myo-Inositol	-0.0104 (-0.0776, 0.0569)	0.7606	0.9701	0.0008 (-0.0694, 0.0709)	0.9827	0.9999
MCPP	N,N-Dimethylglycine	-0.0142 (-0.0754, 0.0470)	0.6457	0.9690	-0.0114 (-0.0752, 0.0525)	0.7247	0.9999
MCPP	O-Acetylcarnitine	-0.0433 (-0.1094, 0.0229)	0.1974	0.9330	-0.0204 (-0.0878, 0.0470)	0.5489	0.9999

		Total Effect			Direct Effect		
**Phthalate**	**Metabolite**	**Estimate (95% CI)**	***p***	**FDR *p***	**Estimate (95% CI)**	***p***	**FDR *p***
MCPP	Ornithine	-0.0025 (-0.1008, 0.0957)	0.9595	0.9901	0.0092 (-0.0890, 0.1074)	0.8529	0.9999
MCPP	Phenylalanine	-0.0206 (-0.0855, 0.0443)	0.5302	0.9556	-0.0216 (-0.0879, 0.0446)	0.5185	0.9999
MCPP	Proline	-0.0055 (-0.0832, 0.0723)	0.8894	0.9756	0.0018 (-0.0781, 0.0816)	0.9648	0.9999
MCPP	Pyruvate	0.0443 (-0.0964, 0.1849)	0.5337	0.9556	0.0578 (-0.0832, 0.1987)	0.4181	0.9999
MCPP	Serine	-0.0216 (-0.0911, 0.0480)	0.5398	0.9556	-0.0200 (-0.0891, 0.0491)	0.5666	0.9999
MCPP	Taurine	-0.0278 (-0.1529, 0.0973)	0.6607	0.9701	-0.0065 (-0.1299, 0.1170)	0.9174	0.9999
MCPP	Threonine	-0.0547 (-0.1639, 0.0544)	0.3222	0.9330	-0.0812 (-0.1934, 0.0310)	0.1543	0.9999
MCPP	Tyrosine	0.0062 (-0.0715, 0.0839)	0.8744	0.9701	0.0070 (-0.0711, 0.0851)	0.8598	0.9999
MCPP	Urea	-0.0416 (-0.1342, 0.0511)	0.3755	0.9330	-0.0480 (-0.1393, 0.0433)	0.2996	0.9999
MCPP	Valine	-0.0325 (-0.1034, 0.0384)	0.3647	0.9330	-0.0174 (-0.0896, 0.0547)	0.6326	0.9999
MECPP	2-Hydroxybutyrate	0.0425 (-0.0785, 0.1635)	0.4872	0.9556	0.0518 (-0.0733, 0.1770)	0.4131	0.9999
MECPP	2-Hydroxyisobutyrate	0.0131 (-0.0483, 0.0746)	0.6719	0.9701	0.0124 (-0.0519, 0.0767)	0.7030	0.9999
MECPP	2-Hydroxyisovalerate	0.0245 (-0.0445, 0.0936)	0.4824	0.9556	0.0201 (-0.0505, 0.0908)	0.5732	0.9999
MECPP	2-Oxoglutarate	0.0052 (-0.0694, 0.0799)	0.8896	0.9756	0.0181 (-0.0573, 0.0935)	0.6344	0.9999
MECPP	2-Oxoisocaproate	-0.0427 (-0.1215, 0.0362)	0.2856	0.9330	-0.0411 (-0.1232, 0.0410)	0.3227	0.9999
MECPP	3-Hydroxybutyrate	-0.0201 (-0.2288, 0.1885)	0.8485	0.9701	-0.0286 (-0.2356, 0.1785)	0.7847	0.9999
MECPP	3-Hydroxyisobutyrate	0.0494 (-0.0545, 0.1533)	0.3475	0.9330	0.0363 (-0.0624, 0.1350)	0.4674	0.9999
MECPP	3-Hydroxyisovalerate	-0.0167 (-0.0782, 0.0448)	0.5916	0.9556	-0.0145 (-0.0761, 0.0471)	0.6420	0.9999
MECPP	3-Methyl-2-oxo-butanoic acid	-0.0153 (-0.0761, 0.0455)	0.6184	0.9642	-0.0137 (-0.0775, 0.0501)	0.6717	0.9999
MECPP	Acetate	-0.0011 (-0.1054, 0.1032)	0.9830	0.9964	0.0028 (-0.1033, 0.1088)	0.9588	0.9999
MECPP	Acetoacetate	-0.0686 (-0.2221, 0.0850)	0.3777	0.9330	-0.0688 (-0.2253, 0.0876)	0.3846	0.9999
MECPP	Acetone	0.0853 (-0.0269, 0.1975)	0.1347	0.9330	0.0728 (-0.0393, 0.1849)	0.2002	0.9999
MECPP	Alanine	0.0455 (-0.0080, 0.0989)	0.0949	0.9330	0.0484 (-0.0059, 0.1028)	0.0801	0.9999
MECPP	Arginine	0.0003 (-0.0788, 0.0794)	0.9940	0.9964	0.0069 (-0.0747, 0.0884)	0.8677	0.9999
MECPP	Asparagine	0.0255 (-0.0500, 0.1011)	0.5037	0.9556	0.0183 (-0.0560, 0.0926)	0.6258	0.9999
MECPP	Aspartate	0.0043 (-0.1087, 0.1173)	0.9400	0.9901	-0.0043 (-0.1152, 0.1066)	0.9390	0.9999
MECPP	Betaine	0.0193 (-0.0422, 0.0808)	0.5343	0.9556	0.0193 (-0.0458, 0.0845)	0.5569	0.9999
MECPP	Carnitine	0.0047 (-0.0586, 0.0679)	0.8839	0.9738	0.0087 (-0.0565, 0.0739)	0.7915	0.9999
MECPP	Choline	0.0637 (-0.0057, 0.1331)	0.0714	0.9330	0.0606 (-0.0101, 0.1313)	0.0922	0.9999
MECPP	Citrate	0.0369 (-0.0313, 0.1052)	0.2853	0.9330	0.0375 (-0.0297, 0.1047)	0.2705	0.9999
MECPP	Creatine	-0.0004 (-0.1114, 0.1107)	0.9949	0.9964	-0.0216 (-0.1339, 0.0908)	0.7041	0.9999
MECPP	Creatinine	0.0234 (-0.0298, 0.0766)	0.3844	0.9360	0.0223 (-0.0324, 0.0770)	0.4194	0.9999
MECPP	Cystine	-0.0232 (-0.1185, 0.0720)	0.6294	0.9677	-0.0103 (-0.1098, 0.0893)	0.8384	0.9999
MECPP	Dimethyl sulfone	0.0365 (-0.0816, 0.1545)	0.5410	0.9556	0.0133 (-0.1012, 0.1278)	0.8183	0.9999
MECPP	Formate	0.0180 (-0.0460, 0.0819)	0.5781	0.9556	0.0131 (-0.0518, 0.0780)	0.6905	0.9999
MECPP	Glucose	-0.0467 (-0.1131, 0.0197)	0.1660	0.9330	-0.0332 (-0.1013, 0.0348)	0.3344	0.9999
MECPP	Glutamate	0.0533 (-0.0576, 0.1643)	0.3422	0.9330	0.0598 (-0.0524, 0.1719)	0.2928	0.9999
MECPP	Glutamine	-0.0239 (-0.0688, 0.0211)	0.2949	0.9330	-0.0323 (-0.0784, 0.0139)	0.1685	0.9999
MECPP	Glycine	0.0499 (-0.0180, 0.1177)	0.1477	0.9330	0.0419 (-0.0277, 0.1114)	0.2349	0.9999
MECPP	Isoleucine	0.0107 (-0.0773, 0.0986)	0.8104	0.9701	0.0081 (-0.0813, 0.0975)	0.8575	0.9999
MECPP	Lactate	0.0533 (-0.0237, 0.1302)	0.1726	0.9330	0.0580 (-0.0196, 0.1356)	0.1414	0.9999
MECPP	Leucine	0.0082 (-0.0671, 0.0835)	0.8292	0.9701	0.0050 (-0.0707, 0.0807)	0.8963	0.9999
MECPP	Lysine	0.0092 (-0.0668, 0.0853)	0.8106	0.9701	0.0035 (-0.0736, 0.0806)	0.9285	0.9999
MECPP	Methanol	-0.0784 (-0.2488, 0.0921)	0.3638	0.9330	-0.1114 (-0.2837, 0.0609)	0.2026	0.9999
MECPP	Methionine	0.0202 (-0.0529, 0.0932)	0.5853	0.9556	0.0111 (-0.0627, 0.0849)	0.7667	0.9999
MECPP	myo-Inositol	0.0014 (-0.0645, 0.0673)	0.9663	0.9924	-0.0004 (-0.0687, 0.0679)	0.9911	0.9999
MECPP	N,N-Dimethylglycine	0.0123 (-0.0476, 0.0723)	0.6844	0.9701	0.0142 (-0.0479, 0.0763)	0.6505	0.9999
MECPP	O-Acetylcarnitine	0.0062 (-0.0591, 0.0715)	0.8508	0.9701	0.0005 (-0.0652, 0.0663)	0.9870	0.9999
MECPP	Ornithine	0.0180 (-0.0782, 0.1141)	0.7118	0.9701	0.0012 (-0.0944, 0.0968)	0.9806	0.9999
MECPP	Phenylalanine	0.0238 (-0.0398, 0.0873)	0.4593	0.9556	0.0145 (-0.0501, 0.0791)	0.6570	0.9999
MECPP	Proline	0.0011 (-0.0751, 0.0773)	0.9766	0.9958	0.0059 (-0.0718, 0.0836)	0.8808	0.9999
MECPP	Pyruvate	0.0732 (-0.0641, 0.2104)	0.2927	0.9330	0.0954 (-0.0410, 0.2317)	0.1682	0.9999
MECPP	Serine	0.0545 (-0.0128, 0.1219)	0.1112	0.9330	0.0455 (-0.0212, 0.1122)	0.1786	0.9999
MECPP	Taurine	0.0689 (-0.0530, 0.1908)	0.2647	0.9330	0.0521 (-0.0676, 0.1718)	0.3895	0.9999
MECPP	Threonine	0.0142 (-0.0932, 0.1216)	0.7942	0.9701	0.0059 (-0.1044, 0.1163)	0.9151	0.9999
MECPP	Tyrosine	0.0062 (-0.0698, 0.0823)	0.8711	0.9701	0.0048 (-0.0712, 0.0808)	0.9001	0.9999
MECPP	Urea	0.0300 (-0.0609, 0.1209)	0.5140	0.9556	0.0104 (-0.0789, 0.0998)	0.8172	0.9999
MECPP	Valine	0.0300 (-0.0395, 0.0995)	0.3936	0.9389	0.0294 (-0.0407, 0.0994)	0.4075	0.9999
MEHHP	2-Hydroxybutyrate	0.0104 (-0.1033, 0.1242)	0.8561	0.9701	0.0225 (-0.0959, 0.1409)	0.7074	0.9999
MEHHP	2-Hydroxyisobutyrate	0.0062 (-0.0514, 0.0639)	0.8304	0.9701	0.0040 (-0.0566, 0.0647)	0.8952	0.9999

		Total Effect			Direct Effect		
**Phthalate**	**Metabolite**	**Estimate (95% CI)**	***p***	**FDR *p***	**Estimate (95% CI)**	***p***	**FDR *p***
MEHHP	2-Hydroxyisovalerate	0.0258 (-0.0389, 0.0905)	0.4314	0.9473	0.0253 (-0.0412, 0.0918)	0.4524	0.9999
MEHHP	2-Oxoglutarate	0.0031 (-0.0669, 0.0731)	0.9297	0.9870	0.0207 (-0.0503, 0.0918)	0.5636	0.9999
MEHHP	2-Oxoisocaproate	-0.0363 (-0.1103, 0.0377)	0.3327	0.9330	-0.0340 (-0.1115, 0.0435)	0.3864	0.9999
MEHHP	3-Hydroxybutyrate	-0.0252 (-0.2209, 0.1704)	0.7985	0.9701	-0.0309 (-0.2262, 0.1643)	0.7539	0.9999
MEHHP	3-Hydroxyisobutyrate	0.0431 (-0.0543, 0.1406)	0.3818	0.9350	0.0290 (-0.0642, 0.1222)	0.5381	0.9999
MEHHP	3-Hydroxyisovalerate	-0.0290 (-0.0865, 0.0285)	0.3193	0.9330	-0.0204 (-0.0785, 0.0376)	0.4868	0.9999
MEHHP	3-Methyl-2-oxo-butanoic acid	-0.0131 (-0.0701, 0.0439)	0.6494	0.9690	-0.0112 (-0.0714, 0.0490)	0.7125	0.9999
MEHHP	Acetate	0.0089 (-0.0889, 0.1067)	0.8568	0.9701	0.0073 (-0.0927, 0.1073)	0.8856	0.9999
MEHHP	Acetoacetate	-0.0315 (-0.1759, 0.1129)	0.6657	0.9701	-0.0336 (-0.1816, 0.1144)	0.6534	0.9999
MEHHP	Acetone	0.0799 (-0.0253, 0.1852)	0.1351	0.9330	0.0710 (-0.0347, 0.1767)	0.1856	0.9999
MEHHP	Alanine	0.0536 (0.0039, 0.1033)	0.0350	0.9330	0.0542 (0.0033, 0.1052)	0.0372	0.9999
MEHHP	Arginine	-0.0083 (-0.0825, 0.0658)	0.8237	0.9701	-0.0133 (-0.0902, 0.0636)	0.7320	0.9999
MEHHP	Asparagine	0.0445 (-0.0259, 0.1149)	0.2130	0.9330	0.0281 (-0.0419, 0.0980)	0.4275	0.9999
MEHHP	Aspartate	0.0048 (-0.1012, 0.1108)	0.9288	0.9870	-0.0161 (-0.1206, 0.0885)	0.7611	0.9999
MEHHP	Betaine	0.0216 (-0.0361, 0.0792)	0.4594	0.9556	0.0217 (-0.0396, 0.0831)	0.4839	0.9999
MEHHP	Carnitine	0.0032 (-0.0562, 0.0625)	0.9156	0.9818	0.0088 (-0.0527, 0.0702)	0.7775	0.9999
MEHHP	Choline	0.0628 (-0.0021, 0.1278)	0.0578	0.9330	0.0589 (-0.0078, 0.1255)	0.0827	0.9999
MEHHP	Citrate	0.0273 (-0.0369, 0.0914)	0.4010	0.9389	0.0407 (-0.0226, 0.1039)	0.2049	0.9999
MEHHP	Creatine	-0.0143 (-0.1184, 0.0898)	0.7862	0.9701	-0.0335 (-0.1393, 0.0724)	0.5317	0.9999
MEHHP	Creatinine	0.0206 (-0.0293, 0.0705)	0.4143	0.9462	0.0195 (-0.0321, 0.0712)	0.4545	0.9999
MEHHP	Cystine	-0.0179 (-0.1073, 0.0715)	0.6921	0.9701	-0.0025 (-0.0965, 0.0914)	0.9571	0.9999
MEHHP	Dimethyl sulfone	0.0393 (-0.0714, 0.1499)	0.4827	0.9556	0.0029 (-0.1051, 0.1109)	0.9570	0.9999
MEHHP	Formate	0.0255 (-0.0343, 0.0854)	0.3993	0.9389	0.0191 (-0.0421, 0.0803)	0.5367	0.9999
MEHHP	Glucose	-0.0439 (-0.1061, 0.0184)	0.1654	0.9330	-0.0298 (-0.0940, 0.0344)	0.3592	0.9999
MEHHP	Glutamate	0.0614 (-0.0424, 0.1652)	0.2432	0.9330	0.0623 (-0.0433, 0.1679)	0.2447	0.9999
MEHHP	Glutamine	-0.0192 (-0.0615, 0.0230)	0.3684	0.9330	-0.0257 (-0.0694, 0.0179)	0.2448	0.9999
MEHHP	Glycine	0.0321 (-0.0319, 0.0961)	0.3217	0.9330	0.0231 (-0.0428, 0.0890)	0.4877	0.9999
MEHHP	Isoleucine	0.0087 (-0.0738, 0.0911)	0.8351	0.9701	0.0029 (-0.0815, 0.0873)	0.9456	0.9999
MEHHP	Lactate	0.0610 (-0.0108, 0.1328)	0.0948	0.9330	0.0604 (-0.0127, 0.1334)	0.1041	0.9999
MEHHP	Leucine	0.0135 (-0.0571, 0.0841)	0.7048	0.9701	0.0040 (-0.0674, 0.0754)	0.9121	0.9999
MEHHP	Lysine	0.0086 (-0.0627, 0.0800)	0.8108	0.9701	-0.0027 (-0.0755, 0.0701)	0.9412	0.9999
MEHHP	Methanol	-0.0977 (-0.2570, 0.0617)	0.2267	0.9330	-0.1225 (-0.2846, 0.0395)	0.1366	0.9999
MEHHP	Methionine	0.0292 (-0.0392, 0.0976)	0.3993	0.9389	0.0130 (-0.0566, 0.0826)	0.7120	0.9999
MEHHP	myo-Inositol	0.0018 (-0.0600, 0.0636)	0.9543	0.9901	-0.0074 (-0.0718, 0.0570)	0.8196	0.9999
MEHHP	N,N-Dimethylglycine	0.0154 (-0.0408, 0.0716)	0.5886	0.9556	0.0119 (-0.0467, 0.0705)	0.6881	0.9999
MEHHP	O-Acetylcarnitine	-0.0024 (-0.0637, 0.0589)	0.9383	0.9901	-0.0120 (-0.0740, 0.0499)	0.7006	0.9999
MEHHP	Ornithine	0.0441 (-0.0458, 0.1339)	0.3327	0.9330	0.0192 (-0.0709, 0.1093)	0.6735	0.9999
MEHHP	Phenylalanine	0.0301 (-0.0293, 0.0896)	0.3173	0.9330	0.0176 (-0.0432, 0.0785)	0.5666	0.9999
MEHHP	Proline	0.0333 (-0.0379, 0.1044)	0.3555	0.9330	0.0318 (-0.0412, 0.1048)	0.3892	0.9999
MEHHP	Pyruvate	0.1197 (-0.0075, 0.2469)	0.0649	0.9330	0.1292 (0.0019, 0.2564)	0.0467	0.9999
MEHHP	Serine	0.0436 (-0.0197, 0.1070)	0.1750	0.9330	0.0303 (-0.0329, 0.0935)	0.3438	0.9999
MEHHP	Taurine	0.0679 (-0.0463, 0.1822)	0.2408	0.9330	0.0383 (-0.0748, 0.1514)	0.5026	0.9999
MEHHP	Threonine	0.0381 (-0.0624, 0.1386)	0.4535	0.9556	0.0210 (-0.0830, 0.1250)	0.6895	0.9999
MEHHP	Tyrosine	0.0194 (-0.0519, 0.0906)	0.5903	0.9556	0.0098 (-0.0619, 0.0815)	0.7874	0.9999
MEHHP	Urea	0.0328 (-0.0524, 0.1180)	0.4462	0.9556	0.0153 (-0.0690, 0.0996)	0.7191	0.9999
MEHHP	Valine	0.0319 (-0.0332, 0.0970)	0.3326	0.9330	0.0284 (-0.0376, 0.0945)	0.3948	0.9999
MEHP	2-Hydroxybutyrate	-0.0581 (-0.1839, 0.0677)	0.3619	0.9330	-0.0505 (-0.1826, 0.0817)	0.4502	0.9999
MEHP	2-Hydroxyisobutyrate	-0.0357 (-0.0994, 0.0279)	0.2675	0.9330	-0.0381 (-0.1055, 0.0294)	0.2653	0.9999
MEHP	2-Hydroxyisovalerate	0.0087 (-0.0633, 0.0808)	0.8104	0.9701	0.0132 (-0.0614, 0.0878)	0.7269	0.9999
MEHP	2-Oxoglutarate	-0.0200 (-0.0976, 0.0577)	0.6112	0.9626	-0.0018 (-0.0814, 0.0778)	0.9639	0.9999
MEHP	2-Oxoisocaproate	-0.0943 (-0.1746, -0.0139)	0.0221	0.9330	-0.0969 (-0.1817, -0.0121)	0.0256	0.9999
MEHP	3-Hydroxybutyrate	-0.1803 (-0.3946, 0.0340)	0.0981	0.9330	-0.1766 (-0.3922, 0.0390)	0.1072	0.9999
MEHP	3-Hydroxyisobutyrate	0.0366 (-0.0718, 0.1450)	0.5042	0.9556	0.0336 (-0.0706, 0.1379)	0.5232	0.9999
MEHP	3-Hydroxyisovalerate	-0.0576 (-0.1207, 0.0055)	0.0732	0.9330	-0.0456 (-0.1100, 0.0188)	0.1633	0.9999
MEHP	3-Methyl-2-oxo-butanoic acid	-0.0490 (-0.1116, 0.0136)	0.1238	0.9330	-0.0520 (-0.1185, 0.0146)	0.1243	0.9999
MEHP	Acetate	0.0317 (-0.0768, 0.1401)	0.5633	0.9556	0.0129 (-0.0990, 0.1247)	0.8197	0.9999
MEHP	Acetoacetate	-0.0731 (-0.2329, 0.0867)	0.3664	0.9330	-0.0679 (-0.2330, 0.0973)	0.4166	0.9999
MEHP	Acetone	0.0799 (-0.0372, 0.1971)	0.1787	0.9330	0.0943 (-0.0234, 0.2121)	0.1151	0.9999
MEHP	Alanine	0.0716 (0.0169, 0.1262)	0.0108	0.9330	0.0693 (0.0128, 0.1259)	0.0168	0.9999

		Total Effect			Direct Effect		
**Phthalate**	**Metabolite**	**Estimate (95% CI)**	***p***	**FDR *p***	**Estimate (95% CI)**	***p***	**FDR *p***
MEHP	Arginine	-0.0374 (-0.1195, 0.0446)	0.3673	0.9330	-0.0343 (-0.1201, 0.0514)	0.4288	0.9999
MEHP	Asparagine	0.0834 (0.0063, 0.1604)	0.0342	0.9330	0.0652 (-0.0122, 0.1426)	0.0976	0.9999
MEHP	Aspartate	-0.0104 (-0.1281, 0.1072)	0.8606	0.9701	-0.0125 (-0.1295, 0.1045)	0.8324	0.9999
MEHP	Betaine	0.0361 (-0.0277, 0.0998)	0.2645	0.9330	0.0452 (-0.0230, 0.1134)	0.1919	0.9999
MEHP	Carnitine	0.0107 (-0.0552, 0.0766)	0.7480	0.9701	0.0139 (-0.0548, 0.0826)	0.6892	0.9999
MEHP	Choline	0.0451 (-0.0278, 0.1180)	0.2228	0.9330	0.0518 (-0.0232, 0.1268)	0.1733	0.9999
MEHP	Citrate	-0.0112 (-0.0826, 0.0602)	0.7566	0.9701	0.0044 (-0.0670, 0.0757)	0.9037	0.9999
MEHP	Creatine	-0.0111 (-0.1267, 0.1046)	0.8497	0.9701	-0.0165 (-0.1351, 0.1021)	0.7827	0.9999
MEHP	Creatinine	0.0237 (-0.0317, 0.0792)	0.3973	0.9389	0.0239 (-0.0338, 0.0816)	0.4129	0.9999
MEHP	Cystine	-0.0277 (-0.1269, 0.0714)	0.5800	0.9556	-0.0224 (-0.1274, 0.0826)	0.6727	0.9999
MEHP	Dimethyl sulfone	0.0630 (-0.0595, 0.1856)	0.3098	0.9330	0.0170 (-0.1037, 0.1378)	0.7803	0.9999
MEHP	Formate	0.0300 (-0.0364, 0.0964)	0.3722	0.9330	0.0189 (-0.0496, 0.0873)	0.5854	0.9999
MEHP	Glucose	-0.0493 (-0.1184, 0.0198)	0.1601	0.9330	-0.0456 (-0.1172, 0.0259)	0.2086	0.9999
MEHP	Glutamate	0.0388 (-0.0770, 0.1546)	0.5078	0.9556	0.0456 (-0.0730, 0.1642)	0.4472	0.9999
MEHP	Glutamine	-0.0178 (-0.0648, 0.0292)	0.4535	0.9556	-0.0172 (-0.0663, 0.0319)	0.4883	0.9999
MEHP	Glycine	0.0301 (-0.0411, 0.1013)	0.4035	0.9416	0.0269 (-0.0468, 0.1006)	0.4707	0.9999
MEHP	Isoleucine	-0.0122 (-0.1038, 0.0793)	0.7916	0.9701	-0.0131 (-0.1075, 0.0812)	0.7829	0.9999
MEHP	Lactate	0.0827 (0.0035, 0.1618)	0.0408	0.9330	0.0817 (0.0006, 0.1629)	0.0484	0.9999
MEHP	Leucine	0.0068 (-0.0716, 0.0852)	0.8633	0.9701	0.0063 (-0.0736, 0.0861)	0.8769	0.9999
MEHP	Lysine	0.0244 (-0.0546, 0.1035)	0.5410	0.9556	0.0274 (-0.0538, 0.1086)	0.5043	0.9999
MEHP	Methanol	-0.0373 (-0.2155, 0.1408)	0.6783	0.9701	-0.0737 (-0.2564, 0.1091)	0.4254	0.9999
MEHP	Methionine	0.0604 (-0.0148, 0.1357)	0.1142	0.9330	0.0499 (-0.0273, 0.1272)	0.2025	0.9999
MEHP	myo-Inositol	0.0110 (-0.0576, 0.0796)	0.7501	0.9701	-0.0081 (-0.0801, 0.0640)	0.8239	0.9999
MEHP	N,N-Dimethylglycine	0.0043 (-0.0582, 0.0668)	0.8914	0.9756	0.0035 (-0.0621, 0.0691)	0.9157	0.9999
MEHP	O-Acetylcarnitine	-0.0494 (-0.1168, 0.0179)	0.1483	0.9330	-0.0599 (-0.1282, 0.0084)	0.0847	0.9999
MEHP	Ornithine	0.0916 (-0.0069, 0.1902)	0.0680	0.9330	0.0681 (-0.0318, 0.1681)	0.1791	0.9999
MEHP	Phenylalanine	0.0379 (-0.0281, 0.1038)	0.2571	0.9330	0.0345 (-0.0334, 0.1023)	0.3160	0.9999
MEHP	Proline	0.0797 (0.0020, 0.1574)	0.0445	0.9330	0.0808 (0.0005, 0.1612)	0.0486	0.9999
MEHP	Pyruvate	0.1352 (-0.0060, 0.2764)	0.0603	0.9330	0.1235 (-0.0196, 0.2666)	0.0900	0.9999
MEHP	Serine	0.0446 (-0.0259, 0.1151)	0.2122	0.9330	0.0448 (-0.0256, 0.1153)	0.2096	0.9999
MEHP	Taurine	0.1097 (-0.0161, 0.2356)	0.0867	0.9330	0.1056 (-0.0194, 0.2306)	0.0968	0.9999
MEHP	Threonine	0.0618 (-0.0494, 0.1730)	0.2726	0.9330	0.0599 (-0.0559, 0.1757)	0.3072	0.9999
MEHP	Tyrosine	0.0642 (-0.0140, 0.1423)	0.1067	0.9330	0.0550 (-0.0244, 0.1344)	0.1724	0.9999
MEHP	Urea	0.0479 (-0.0465, 0.1423)	0.3163	0.9330	0.0310 (-0.0631, 0.1251)	0.5146	0.9999
MEHP	Valine	0.0369 (-0.0354, 0.1091)	0.3136	0.9330	0.0366 (-0.0372, 0.1104)	0.3274	0.9999
MEOHP	2-Hydroxybutyrate	0.0050 (-0.1178, 0.1279)	0.9353	0.9901	0.0118 (-0.1160, 0.1397)	0.8546	0.9999
MEOHP	2-Hydroxyisobutyrate	0.0056 (-0.0566, 0.0679)	0.8581	0.9701	0.0014 (-0.0641, 0.0669)	0.9658	0.9999
MEOHP	2-Hydroxyisovalerate	0.0282 (-0.0417, 0.0981)	0.4259	0.9462	0.0263 (-0.0455, 0.0981)	0.4689	0.9999
MEOHP	2-Oxoglutarate	0.0069 (-0.0687, 0.0826)	0.8561	0.9701	0.0235 (-0.0532, 0.1002)	0.5448	0.9999
MEOHP	2-Oxoisocaproate	-0.0430 (-0.1229, 0.0369)	0.2881	0.9330	-0.0432 (-0.1268, 0.0403)	0.3067	0.9999
MEOHP	3-Hydroxybutyrate	-0.0255 (-0.2368, 0.1859)	0.8115	0.9701	-0.0389 (-0.2496, 0.1718)	0.7146	0.9999
MEOHP	3-Hydroxyisobutyrate	0.0336 (-0.0719, 0.1390)	0.5293	0.9556	0.0145 (-0.0862, 0.1152)	0.7757	0.9999
MEOHP	3-Hydroxyisovalerate	-0.0350 (-0.0970, 0.0270)	0.2655	0.9330	-0.0276 (-0.0902, 0.0349)	0.3828	0.9999
MEOHP	3-Methyl-2-oxo-butanoic acid	-0.0192 (-0.0808, 0.0423)	0.5370	0.9556	-0.0191 (-0.0840, 0.0458)	0.5608	0.9999
MEOHP	Acetate	0.0004 (-0.1053, 0.1060)	0.9947	0.9964	0.0000 (-0.1079, 0.1079)	0.9999	0.9999
MEOHP	Acetoacetate	-0.0309 (-0.1869, 0.1251)	0.6951	0.9701	-0.0362 (-0.1959, 0.1235)	0.6537	0.9999
MEOHP	Acetone	0.0872 (-0.0265, 0.2008)	0.1312	0.9330	0.0753 (-0.0388, 0.1893)	0.1935	0.9999
MEOHP	Alanine	0.0509 (-0.0031, 0.1049)	0.0642	0.9330	0.0521 (-0.0031, 0.1074)	0.0642	0.9999
MEOHP	Arginine	-0.0016 (-0.0817, 0.0785)	0.9683	0.9924	-0.0071 (-0.0901, 0.0759)	0.8648	0.9999
MEOHP	Asparagine	0.0482 (-0.0279, 0.1242)	0.2118	0.9330	0.0320 (-0.0435, 0.1075)	0.4019	0.9999
MEOHP	Aspartate	0.0204 (-0.0940, 0.1348)	0.7237	0.9701	-0.0024 (-0.1154, 0.1105)	0.9657	0.9999
MEOHP	Betaine	0.0238 (-0.0384, 0.0861)	0.4496	0.9556	0.0224 (-0.0438, 0.0887)	0.5030	0.9999
MEOHP	Carnitine	0.0032 (-0.0609, 0.0673)	0.9214	0.9829	0.0076 (-0.0588, 0.0739)	0.8214	0.9999
MEOHP	Choline	0.0705 (0.0005, 0.1406)	0.0485	0.9330	0.0640 (-0.0079, 0.1359)	0.0805	0.9999
MEOHP	Citrate	0.0363 (-0.0329, 0.1054)	0.3002	0.9330	0.0485 (-0.0196, 0.1166)	0.1608	0.9999
MEOHP	Creatine	-0.0220 (-0.1344, 0.0904)	0.6987	0.9701	-0.0437 (-0.1578, 0.0705)	0.4494	0.9999
MEOHP	Creatinine	0.0249 (-0.0290, 0.0787)	0.3621	0.9330	0.0234 (-0.0322, 0.0791)	0.4054	0.9999
MEOHP	Cystine	-0.0096 (-0.1062, 0.0869)	0.8433	0.9701	0.0057 (-0.0957, 0.1070)	0.9120	0.9999
MEOHP	Dimethyl sulfone	0.0397 (-0.0798, 0.1593)	0.5110	0.9556	0.0045 (-0.1120, 0.1211)	0.9388	0.9999
MEOHP	Formate	0.0254 (-0.0392, 0.0901)	0.4373	0.9542	0.0186 (-0.0474, 0.0846)	0.5773	0.9999

		Total Effect			Direct Effect		
**Phthalate**	**Metabolite**	**Estimate (95% CI)**	***p***	**FDR *p***	**Estimate (95% CI)**	***p***	**FDR *p***
MEOHP	Glucose	-0.0557 (-0.1227, 0.0113)	0.1024	0.9330	-0.0405 (-0.1096, 0.0286)	0.2479	0.9999
MEOHP	Glutamate	0.0744 (-0.0375, 0.1863)	0.1899	0.9330	0.0743 (-0.0396, 0.1881)	0.1984	0.9999
MEOHP	Glutamine	-0.0208 (-0.0664, 0.0249)	0.3690	0.9330	-0.0275 (-0.0746, 0.0196)	0.2498	0.9999
MEOHP	Glycine	0.0352 (-0.0339, 0.1043)	0.3149	0.9330	0.0260 (-0.0451, 0.0971)	0.4702	0.9999
MEOHP	Isoleucine	0.0086 (-0.0805, 0.0976)	0.8490	0.9701	0.0013 (-0.0898, 0.0923)	0.9777	0.9999
MEOHP	Lactate	0.0667 (-0.0108, 0.1443)	0.0907	0.9330	0.0662 (-0.0126, 0.1450)	0.0986	0.9999
MEOHP	Leucine	0.0122 (-0.0641, 0.0884)	0.7523	0.9701	0.0006 (-0.0764, 0.0777)	0.9870	0.9999
MEOHP	Lysine	0.0129 (-0.0641, 0.0899)	0.7399	0.9701	-0.0004 (-0.0789, 0.0782)	0.9929	0.9999
MEOHP	Methanol	-0.1284 (-0.2999, 0.0431)	0.1405	0.9330	-0.1577 (-0.3317, 0.0163)	0.0751	0.9999
MEOHP	Methionine	0.0238 (-0.0502, 0.0978)	0.5245	0.9556	0.0067 (-0.0685, 0.0818)	0.8603	0.9999
MEOHP	myo-Inositol	0.0072 (-0.0595, 0.0740)	0.8307	0.9701	-0.0032 (-0.0727, 0.0663)	0.9275	0.9999
MEOHP	N,N-Dimethylglycine	0.0158 (-0.0449, 0.0765)	0.6067	0.9623	0.0107 (-0.0525, 0.0739)	0.7378	0.9999
MEOHP	O-Acetylcarnitine	-0.0055 (-0.0717, 0.0607)	0.8696	0.9701	-0.0193 (-0.0861, 0.0475)	0.5670	0.9999
MEOHP	Ornithine	0.0574 (-0.0394, 0.1542)	0.2424	0.9330	0.0316 (-0.0655, 0.1288)	0.5194	0.9999
MEOHP	Phenylalanine	0.0308 (-0.0335, 0.0950)	0.3443	0.9330	0.0171 (-0.0486, 0.0828)	0.6072	0.9999
MEOHP	Proline	0.0418 (-0.0349, 0.1185)	0.2825	0.9330	0.0404 (-0.0383, 0.1191)	0.3103	0.9999
MEOHP	Pyruvate	0.1289 (-0.0085, 0.2663)	0.0657	0.9330	0.1430 (0.0059, 0.2802)	0.0411	0.9999
MEOHP	Serine	0.0518 (-0.0165, 0.1202)	0.1353	0.9330	0.0378 (-0.0303, 0.1059)	0.2733	0.9999
MEOHP	Taurine	0.0814 (-0.0418, 0.2046)	0.1927	0.9330	0.0488 (-0.0731, 0.1707)	0.4289	0.9999
MEOHP	Threonine	0.0490 (-0.0594, 0.1574)	0.3715	0.9330	0.0321 (-0.0801, 0.1443)	0.5712	0.9999
MEOHP	Tyrosine	0.0229 (-0.0541, 0.0998)	0.5567	0.9556	0.0136 (-0.0637, 0.0910)	0.7274	0.9999
MEOHP	Urea	0.0411 (-0.0508, 0.1330)	0.3773	0.9330	0.0215 (-0.0694, 0.1125)	0.6391	0.9999
MEOHP	Valine	0.0346 (-0.0357, 0.1050)	0.3306	0.9330	0.0299 (-0.0414, 0.1012)	0.4074	0.9999
MEP	2-Hydroxybutyrate	-0.0689 (-0.1563, 0.0185)	0.1208	0.9330	-0.0576 (-0.1482, 0.0329)	0.2097	0.9999
MEP	2-Hydroxyisobutyrate	-0.0053 (-0.0501, 0.0395)	0.8139	0.9701	0.0000 (-0.0468, 0.0468)	0.9999	0.9999
MEP	2-Hydroxyisovalerate	-0.0225 (-0.0728, 0.0278)	0.3762	0.9330	-0.0172 (-0.0685, 0.0341)	0.5079	0.9999
MEP	2-Oxoglutarate	-0.0220 (-0.0763, 0.0322)	0.4224	0.9462	-0.0096 (-0.0644, 0.0452)	0.7288	0.9999
MEP	2-Oxoisocaproate	-0.0444 (-0.1016, 0.0127)	0.1260	0.9330	-0.0388 (-0.0983, 0.0207)	0.1985	0.9999
MEP	3-Hydroxybutyrate	-0.1732 (-0.3214, -0.0251)	0.0224	0.9330	-0.1625 (-0.3095, -0.0156)	0.0305	0.9999
MEP	3-Hydroxyisobutyrate	-0.0211 (-0.0970, 0.0549)	0.5832	0.9556	-0.0246 (-0.0964, 0.0472)	0.4986	0.9999
MEP	3-Hydroxyisovalerate	-0.0087 (-0.0536, 0.0361)	0.6997	0.9701	0.0028 (-0.0420, 0.0477)	0.8999	0.9999
MEP	3-Methyl-2-oxo-butanoic acid	-0.0418 (-0.0854, 0.0018)	0.0598	0.9330	-0.0393 (-0.0850, 0.0065)	0.0917	0.9999
MEP	Acetate	0.0260 (-0.0499, 0.1018)	0.4990	0.9556	0.0114 (-0.0657, 0.0885)	0.7696	0.9999
MEP	Acetoacetate	-0.1543 (-0.2623, -0.0462)	0.0056	0.9330	-0.1487 (-0.2588, -0.0385)	0.0087	0.9999
MEP	Acetone	-0.0463 (-0.1286, 0.0359)	0.2666	0.9330	-0.0383 (-0.1202, 0.0435)	0.3550	0.9999
MEP	Alanine	0.0194 (-0.0200, 0.0588)	0.3309	0.9330	0.0121 (-0.0280, 0.0522)	0.5521	0.9999
MEP	Arginine	-0.0020 (-0.0597, 0.0557)	0.9445	0.9901	0.0057 (-0.0535, 0.0650)	0.8481	0.9999
MEP	Asparagine	0.0116 (-0.0436, 0.0667)	0.6777	0.9701	0.0000 (-0.0541, 0.0541)	0.9995	0.9999
MEP	Aspartate	0.0194 (-0.0630, 0.1017)	0.6417	0.9690	0.0189 (-0.0617, 0.0994)	0.6427	0.9999
MEP	Betaine	0.0242 (-0.0204, 0.0689)	0.2844	0.9330	0.0369 (-0.0099, 0.0838)	0.1208	0.9999
MEP	Carnitine	0.0426 (-0.0028, 0.0879)	0.0654	0.9330	0.0446 (-0.0019, 0.0911)	0.0600	0.9999
MEP	Choline	0.0271 (-0.0241, 0.0783)	0.2958	0.9330	0.0365 (-0.0151, 0.0882)	0.1634	0.9999
MEP	Citrate	0.0099 (-0.0401, 0.0599)	0.6957	0.9701	0.0235 (-0.0255, 0.0724)	0.3436	0.9999
MEP	Creatine	-0.0015 (-0.0825, 0.0795)	0.9703	0.9924	0.0003 (-0.0815, 0.0820)	0.9946	0.9999
MEP	Creatinine	0.0321 (-0.0063, 0.0705)	0.1006	0.9330	0.0343 (-0.0050, 0.0736)	0.0867	0.9999
MEP	Cystine	-0.0118 (-0.0813, 0.0577)	0.7362	0.9701	-0.0061 (-0.0785, 0.0663)	0.8667	0.9999
MEP	Dimethyl sulfone	0.0681 (-0.0171, 0.1533)	0.1159	0.9330	0.0434 (-0.0394, 0.1261)	0.3011	0.9999
MEP	Formate	0.0130 (-0.0336, 0.0597)	0.5802	0.9556	0.0039 (-0.0433, 0.0511)	0.8702	0.9999
MEP	Glucose	-0.0016 (-0.0505, 0.0473)	0.9498	0.9901	-0.0014 (-0.0511, 0.0483)	0.9547	0.9999
MEP	Glutamate	-0.0118 (-0.0931, 0.0694)	0.7729	0.9701	-0.0103 (-0.0922, 0.0717)	0.8044	0.9999
MEP	Glutamine	0.0162 (-0.0167, 0.0490)	0.3313	0.9330	0.0197 (-0.0140, 0.0533)	0.2490	0.9999
MEP	Glycine	0.0492 (0.0001, 0.0982)	0.0493	0.9330	0.0493 (-0.0006, 0.0992)	0.0529	0.9999
MEP	Isoleucine	-0.0116 (-0.0757, 0.0524)	0.7192	0.9701	-0.0117 (-0.0767, 0.0533)	0.7221	0.9999
MEP	Lactate	0.0138 (-0.0428, 0.0703)	0.6295	0.9677	0.0078 (-0.0493, 0.0649)	0.7866	0.9999
MEP	Leucine	-0.0068 (-0.0617, 0.0481)	0.8056	0.9701	-0.0058 (-0.0608, 0.0492)	0.8347	0.9999
MEP	Lysine	0.0067 (-0.0488, 0.0621)	0.8121	0.9701	0.0108 (-0.0453, 0.0668)	0.7037	0.9999
MEP	Methanol	-0.0362 (-0.1608, 0.0884)	0.5657	0.9556	-0.0602 (-0.1860, 0.0655)	0.3441	0.9999
MEP	Methionine	0.0172 (-0.0361, 0.0705)	0.5230	0.9556	0.0104 (-0.0433, 0.0640)	0.7017	0.9999
MEP	myo-Inositol	-0.0201 (-0.0680, 0.0278)	0.4080	0.9453	-0.0315 (-0.0808, 0.0177)	0.2071	0.9999
MEP	N,N-Dimethylglycine	0.0213 (-0.0223, 0.0649)	0.3343	0.9330	0.0259 (-0.0190, 0.0708)	0.2547	0.9999

		Total Effect			Direct Effect		
**Phthalate**	**Metabolite**	**Estimate (95% CI)**	***p***	**FDR *p***	**Estimate (95% CI)**	***p***	**FDR *p***
MEP	O-Acetylcarnitine	-0.0107 (-0.0583, 0.0369)	0.6573	0.9701	-0.0095 (-0.0573, 0.0382)	0.6935	0.9999
MEP	Ornithine	0.0390 (-0.0307, 0.1088)	0.2696	0.9330	0.0209 (-0.0485, 0.0903)	0.5505	0.9999
MEP	Phenylalanine	0.0437 (-0.0020, 0.0893)	0.0605	0.9330	0.0436 (-0.0026, 0.0897)	0.0640	0.9999
MEP	Proline	0.0179 (-0.0375, 0.0734)	0.5228	0.9556	0.0164 (-0.0400, 0.0728)	0.5658	0.9999
MEP	Pyruvate	0.0373 (-0.0631, 0.1377)	0.4623	0.9556	0.0118 (-0.0883, 0.1119)	0.8156	0.9999
MEP	Serine	0.0275 (-0.0219, 0.0770)	0.2717	0.9330	0.0271 (-0.0215, 0.0758)	0.2714	0.9999
MEP	Taurine	0.0210 (-0.0683, 0.1104)	0.6418	0.9690	0.0172 (-0.0701, 0.1045)	0.6966	0.9999
MEP	Threonine	-0.0169 (-0.0952, 0.0614)	0.6694	0.9701	-0.0150 (-0.0952, 0.0652)	0.7114	0.9999
MEP	Tyrosine	0.0219 (-0.0334, 0.0772)	0.4347	0.9514	0.0132 (-0.0420, 0.0684)	0.6364	0.9999
MEP	Urea	-0.0018 (-0.0683, 0.0646)	0.9562	0.9901	-0.0102 (-0.0751, 0.0548)	0.7567	0.9999
MEP	Valine	-0.0090 (-0.0598, 0.0418)	0.7257	0.9701	-0.0115 (-0.0625, 0.0396)	0.6561	0.9999
MHBP	2-Hydroxybutyrate	-0.0532 (-0.3298, 0.2233)	0.7032	0.9701	-0.0233 (-0.3037, 0.2571)	0.8691	0.9999
MHBP	2-Hydroxyisobutyrate	-0.0726 (-0.2121, 0.0669)	0.3040	0.9330	-0.0580 (-0.2012, 0.0851)	0.4232	0.9999
MHBP	2-Hydroxyisovalerate	-0.0640 (-0.2214, 0.0934)	0.4219	0.9462	-0.0566 (-0.2142, 0.1010)	0.4775	0.9999
MHBP	2-Oxoglutarate	-0.0273 (-0.1976, 0.1429)	0.7509	0.9701	-0.0142 (-0.1827, 0.1543)	0.8677	0.9999
MHBP	2-Oxoisocaproate	-0.0976 (-0.2775, 0.0822)	0.2841	0.9330	-0.0806 (-0.2641, 0.1029)	0.3854	0.9999
MHBP	3-Hydroxybutyrate	0.0848 (-0.3909, 0.5606)	0.7242	0.9701	0.0824 (-0.3797, 0.5445)	0.7243	0.9999
MHBP	3-Hydroxyisobutyrate	-0.1198 (-0.3566, 0.1170)	0.3178	0.9330	-0.1262 (-0.3458, 0.0933)	0.2565	0.9999
MHBP	3-Hydroxyisovalerate	-0.0326 (-0.1729, 0.1078)	0.6464	0.9690	-0.0242 (-0.1619, 0.1134)	0.7274	0.9999
MHBP	3-Methyl-2-oxo-butanoic acid	-0.1254 (-0.2620, 0.0111)	0.0714	0.9330	-0.1105 (-0.2514, 0.0303)	0.1225	0.9999
MHBP	Acetate	-0.0947 (-0.3320, 0.1425)	0.4301	0.9473	-0.0890 (-0.3250, 0.1471)	0.4562	0.9999
MHBP	Acetoacetate	0.0435 (-0.3080, 0.3951)	0.8064	0.9701	0.0479 (-0.3026, 0.3984)	0.7868	0.9999
MHBP	Acetone	0.1458 (-0.1116, 0.4031)	0.2637	0.9330	0.1433 (-0.1074, 0.3941)	0.2594	0.9999
MHBP	Alanine	-0.0689 (-0.1920, 0.0541)	0.2688	0.9330	-0.0759 (-0.1983, 0.0465)	0.2214	0.9999
MHBP	Arginine	-0.1025 (-0.2818, 0.0768)	0.2594	0.9330	-0.0916 (-0.2727, 0.0895)	0.3181	0.9999
MHBP	Asparagine	0.0261 (-0.1465, 0.1987)	0.7644	0.9701	0.0309 (-0.1351, 0.1969)	0.7129	0.9999
MHBP	Aspartate	0.0489 (-0.2088, 0.3065)	0.7073	0.9701	0.0303 (-0.2172, 0.2779)	0.8083	0.9999
MHBP	Betaine	-0.0444 (-0.1847, 0.0959)	0.5317	0.9556	-0.0228 (-0.1684, 0.1227)	0.7563	0.9999
MHBP	Carnitine	-0.0740 (-0.2176, 0.0696)	0.3091	0.9330	-0.0708 (-0.2156, 0.0741)	0.3344	0.9999
MHBP	Choline	-0.0582 (-0.2187, 0.1024)	0.4737	0.9556	-0.0448 (-0.2048, 0.1152)	0.5798	0.9999
MHBP	Citrate	-0.0162 (-0.1728, 0.1403)	0.8373	0.9701	-0.0010 (-0.1520, 0.1500)	0.9893	0.9999
MHBP	Creatine	-0.0358 (-0.2891, 0.2175)	0.7796	0.9701	-0.0357 (-0.2866, 0.2152)	0.7782	0.9999
MHBP	Creatinine	0.0216 (-0.1002, 0.1433)	0.7261	0.9701	0.0277 (-0.0947, 0.1501)	0.6544	0.9999
MHBP	Cystine	0.0114 (-0.2062, 0.2290)	0.9175	0.9818	0.0323 (-0.1899, 0.2546)	0.7734	0.9999
MHBP	Dimethyl sulfone	-0.1586 (-0.4265, 0.1094)	0.2431	0.9330	-0.1628 (-0.4164, 0.0907)	0.2054	0.9999
MHBP	Formate	-0.0586 (-0.2042, 0.0870)	0.4266	0.9462	-0.0619 (-0.2064, 0.0826)	0.3974	0.9999
MHBP	Glucose	-0.0869 (-0.2389, 0.0651)	0.2594	0.9330	-0.0844 (-0.2361, 0.0672)	0.2719	0.9999
MHBP	Glutamate	0.0013 (-0.2529, 0.2555)	0.9920	0.9964	-0.0038 (-0.2556, 0.2480)	0.9763	0.9999
MHBP	Glutamine	-0.0336 (-0.1366, 0.0694)	0.5192	0.9556	-0.0331 (-0.1370, 0.0708)	0.5284	0.9999
MHBP	Glycine	-0.0433 (-0.1995, 0.1129)	0.5834	0.9556	-0.0443 (-0.2004, 0.1119)	0.5750	0.9999
MHBP	Isoleucine	-0.0245 (-0.2251, 0.1761)	0.8088	0.9701	-0.0292 (-0.2288, 0.1704)	0.7723	0.9999
MHBP	Lactate	0.0727 (-0.1038, 0.2493)	0.4157	0.9462	0.0677 (-0.1070, 0.2425)	0.4437	0.9999
MHBP	Leucine	-0.0292 (-0.2009, 0.1425)	0.7367	0.9701	-0.0321 (-0.2010, 0.1369)	0.7072	0.9999
MHBP	Lysine	-0.0145 (-0.1880, 0.1591)	0.8689	0.9701	-0.0182 (-0.1904, 0.1540)	0.8344	0.9999
MHBP	Methanol	-0.5183 (-0.8948, -0.1418)	0.0075	0.9330	-0.5258 (-0.8989, -0.1527)	0.0062	0.9999
MHBP	Methionine	-0.0231 (-0.1900, 0.1438)	0.7840	0.9701	-0.0277 (-0.1925, 0.1370)	0.7389	0.9999
MHBP	myo-Inositol	-0.0418 (-0.1919, 0.1083)	0.5817	0.9556	-0.0362 (-0.1885, 0.1162)	0.6384	0.9999
MHBP	N,N-Dimethylglycine	-0.0718 (-0.2080, 0.0643)	0.2977	0.9330	-0.0567 (-0.1950, 0.0816)	0.4176	0.9999
MHBP	O-Acetylcarnitine	-0.0153 (-0.1643, 0.1338)	0.8394	0.9701	-0.0065 (-0.1532, 0.1402)	0.9302	0.9999
MHBP	Ornithine	0.0708 (-0.1483, 0.2899)	0.5231	0.9556	0.0508 (-0.1624, 0.2641)	0.6373	0.9999
MHBP	Phenylalanine	0.0038 (-0.1416, 0.1491)	0.9593	0.9901	0.0003 (-0.1441, 0.1446)	0.9969	0.9999
MHBP	Proline	0.0530 (-0.1205, 0.2265)	0.5456	0.9556	0.0521 (-0.1210, 0.2253)	0.5514	0.9999
MHBP	Pyruvate	0.0346 (-0.2803, 0.3494)	0.8280	0.9701	0.0148 (-0.2926, 0.3222)	0.9241	0.9999
MHBP	Serine	0.0124 (-0.1432, 0.1680)	0.8748	0.9701	0.0039 (-0.1465, 0.1542)	0.9595	0.9999
MHBP	Taurine	0.0999 (-0.1792, 0.3791)	0.4791	0.9556	0.0829 (-0.1849, 0.3507)	0.5404	0.9999
MHBP	Threonine	0.0401 (-0.2049, 0.2851)	0.7459	0.9701	0.0482 (-0.1981, 0.2944)	0.6986	0.9999
MHBP	Tyrosine	0.0022 (-0.1714, 0.1757)	0.9803	0.9964	-0.0013 (-0.1711, 0.1684)	0.9875	0.9999
MHBP	Urea	0.0128 (-0.1950, 0.2206)	0.9027	0.9782	0.0191 (-0.1805, 0.2187)	0.8500	0.9999
MHBP	Valine	-0.0163 (-0.1754, 0.1427)	0.8389	0.9701	-0.0198 (-0.1767, 0.1371)	0.8023	0.9999
MHiBP	2-Hydroxybutyrate	-0.0453 (-0.2054, 0.1148)	0.5760	0.9556	-0.0252 (-0.1948, 0.1445)	0.7690	0.9999

		Total Effect			Direct Effect		
**Phthalate**	**Metabolite**	**Estimate (95% CI)**	***p***	**FDR *p***	**Estimate (95% CI)**	***p***	**FDR *p***
MHiBP	2-Hydroxyisobutyrate	-0.0567 (-0.1372, 0.0237)	0.1650	0.9330	-0.0545 (-0.1407, 0.0317)	0.2123	0.9999
MHiBP	2-Hydroxyisovalerate	-0.0516 (-0.1425, 0.0393)	0.2630	0.9330	-0.0493 (-0.1443, 0.0458)	0.3063	0.9999
MHiBP	2-Oxoglutarate	-0.0619 (-0.1598, 0.0360)	0.2125	0.9330	-0.0335 (-0.1353, 0.0682)	0.5144	0.9999
MHiBP	2-Oxoisocaproate	-0.0138 (-0.1186, 0.0910)	0.7949	0.9701	-0.0002 (-0.1116, 0.1113)	0.9977	0.9999
MHiBP	3-Hydroxybutyrate	0.1459 (-0.1285, 0.4202)	0.2939	0.9330	0.1932 (-0.0838, 0.4703)	0.1695	0.9999
MHiBP	3-Hydroxyisobutyrate	-0.0957 (-0.2322, 0.0409)	0.1677	0.9330	-0.1273 (-0.2585, 0.0039)	0.0571	0.9999
MHiBP	3-Hydroxyisovalerate	-0.0427 (-0.1237, 0.0383)	0.2983	0.9330	-0.0202 (-0.1035, 0.0630)	0.6307	0.9999
MHiBP	3-Methyl-2-oxo-butanoic acid	-0.0392 (-0.1193, 0.0409)	0.3342	0.9330	-0.0355 (-0.1215, 0.0505)	0.4144	0.9999
MHiBP	Acetate	0.0563 (-0.0811, 0.1938)	0.4179	0.9462	0.0286 (-0.1145, 0.1717)	0.6924	0.9999
MHiBP	Acetoacetate	0.0569 (-0.1465, 0.2603)	0.5801	0.9556	0.0853 (-0.1262, 0.2968)	0.4252	0.9999
MHiBP	Acetone	0.1202 (-0.0279, 0.2683)	0.1105	0.9330	0.1297 (-0.0208, 0.2801)	0.0904	0.9999
MHiBP	Alanine	-0.0084 (-0.0801, 0.0633)	0.8175	0.9701	-0.0250 (-0.0995, 0.0495)	0.5065	0.9999
MHiBP	Arginine	-0.0574 (-0.1613, 0.0466)	0.2762	0.9330	-0.0490 (-0.1587, 0.0607)	0.3772	0.9999
MHiBP	Asparagine	0.0407 (-0.0590, 0.1404)	0.4200	0.9462	0.0069 (-0.0936, 0.1074)	0.8917	0.9999
MHiBP	Aspartate	-0.0190 (-0.1684, 0.1303)	0.8012	0.9701	-0.0327 (-0.1824, 0.1170)	0.6654	0.9999
MHiBP	Betaine	-0.0408 (-0.1218, 0.0403)	0.3209	0.9330	-0.0276 (-0.1155, 0.0604)	0.5350	0.9999
MHiBP	Carnitine	-0.0543 (-0.1372, 0.0287)	0.1973	0.9330	-0.0553 (-0.1427, 0.0320)	0.2118	0.9999
MHiBP	Choline	-0.0217 (-0.1149, 0.0714)	0.6445	0.9690	-0.0178 (-0.1147, 0.0791)	0.7161	0.9999
MHiBP	Citrate	-0.0466 (-0.1368, 0.0437)	0.3082	0.9330	-0.0276 (-0.1187, 0.0636)	0.5500	0.9999
MHiBP	Creatine	-0.0399 (-0.1865, 0.1067)	0.5901	0.9556	-0.0603 (-0.2117, 0.0911)	0.4309	0.9999
MHiBP	Creatinine	0.0043 (-0.0663, 0.0749)	0.9040	0.9782	0.0024 (-0.0718, 0.0765)	0.9489	0.9999
MHiBP	Cystine	-0.0352 (-0.1611, 0.0906)	0.5797	0.9556	-0.0206 (-0.1550, 0.1139)	0.7622	0.9999
MHiBP	Dimethyl sulfone	-0.0595 (-0.2154, 0.0964)	0.4506	0.9556	-0.1352 (-0.2875, 0.0170)	0.0811	0.9999
MHiBP	Formate	0.0119 (-0.0727, 0.0965)	0.7807	0.9701	-0.0124 (-0.1002, 0.0753)	0.7791	0.9999
MHiBP	Glucose	-0.0378 (-0.1261, 0.0505)	0.3974	0.9389	-0.0283 (-0.1205, 0.0639)	0.5438	0.9999
MHiBP	Glutamate	0.0102 (-0.1371, 0.1575)	0.8906	0.9756	0.0171 (-0.1352, 0.1694)	0.8238	0.9999
MHiBP	Glutamine	0.0202 (-0.0395, 0.0799)	0.5036	0.9556	0.0202 (-0.0427, 0.0830)	0.5260	0.9999
MHiBP	Glycine	0.0208 (-0.0698, 0.1113)	0.6503	0.9690	0.0093 (-0.0853, 0.1039)	0.8455	0.9999
MHiBP	Isoleucine	-0.0372 (-0.1532, 0.0789)	0.5266	0.9556	-0.0422 (-0.1627, 0.0783)	0.4889	0.9999
MHiBP	Lactate	0.0444 (-0.0579, 0.1467)	0.3912	0.9389	0.0340 (-0.0718, 0.1398)	0.5252	0.9999
MHiBP	Leucine	-0.0472 (-0.1463, 0.0518)	0.3464	0.9330	-0.0537 (-0.1554, 0.0480)	0.2973	0.9999
MHiBP	Lysine	-0.0475 (-0.1477, 0.0526)	0.3485	0.9330	-0.0493 (-0.1530, 0.0545)	0.3483	0.9999
MHiBP	Methanol	-0.1559 (-0.3800, 0.0682)	0.1706	0.9330	-0.2351 (-0.4650, -0.0052)	0.0451	0.9999
MHiBP	Methionine	-0.0332 (-0.1298, 0.0633)	0.4960	0.9556	-0.0608 (-0.1597, 0.0382)	0.2260	0.9999
MHiBP	myo-Inositol	0.0202 (-0.0668, 0.1073)	0.6453	0.9690	-0.0033 (-0.0956, 0.0890)	0.9435	0.9999
MHiBP	N,N-Dimethylglycine	-0.0511 (-0.1298, 0.0276)	0.2005	0.9330	-0.0545 (-0.1377, 0.0288)	0.1969	0.9999
MHiBP	O-Acetylcarnitine	0.0177 (-0.0686, 0.1040)	0.6853	0.9701	0.0184 (-0.0703, 0.1071)	0.6817	0.9999
MHiBP	Ornithine	0.0569 (-0.0699, 0.1836)	0.3754	0.9330	0.0140 (-0.1152, 0.1432)	0.8301	0.9999
MHiBP	Phenylalanine	-0.0435 (-0.1272, 0.0403)	0.3059	0.9330	-0.0603 (-0.1468, 0.0262)	0.1694	0.9999
MHiBP	Proline	-0.0117 (-0.1124, 0.0890)	0.8177	0.9701	-0.0130 (-0.1179, 0.0920)	0.8067	0.9999
MHiBP	Pyruvate	0.1475 (-0.0325, 0.3276)	0.1072	0.9330	0.1169 (-0.0676, 0.3014)	0.2116	0.9999
MHiBP	Serine	0.0245 (-0.0656, 0.1145)	0.5909	0.9556	0.0096 (-0.0814, 0.1005)	0.8346	0.9999
MHiBP	Taurine	0.0426 (-0.1193, 0.2045)	0.6028	0.9623	0.0165 (-0.1458, 0.1788)	0.8402	0.9999
MHiBP	Threonine	0.0277 (-0.1142, 0.1696)	0.6993	0.9701	0.0172 (-0.1319, 0.1663)	0.8194	0.9999
MHiBP	Tyrosine	-0.0164 (-0.1169, 0.0841)	0.7466	0.9701	-0.0425 (-0.1448, 0.0599)	0.4121	0.9999
MHiBP	Urea	-0.0598 (-0.1796, 0.0600)	0.3244	0.9330	-0.1040 (-0.2229, 0.0150)	0.0859	0.9999
MHiBP	Valine	-0.0449 (-0.1367, 0.0469)	0.3339	0.9330	-0.0564 (-0.1507, 0.0378)	0.2377	0.9999
MiBP	2-Hydroxybutyrate	-0.0625 (-0.1958, 0.0708)	0.3543	0.9330	-0.0484 (-0.1869, 0.0900)	0.4893	0.9999
MiBP	2-Hydroxyisobutyrate	-0.0623 (-0.1290, 0.0044)	0.0670	0.9330	-0.0566 (-0.1268, 0.0136)	0.1127	0.9999
MiBP	2-Hydroxyisovalerate	-0.0457 (-0.1216, 0.0302)	0.2348	0.9330	-0.0409 (-0.1186, 0.0369)	0.2996	0.9999
MiBP	2-Oxoglutarate	-0.0545 (-0.1362, 0.0272)	0.1884	0.9330	-0.0348 (-0.1179, 0.0483)	0.4084	0.9999
MiBP	2-Oxoisocaproate	-0.0335 (-0.1208, 0.0537)	0.4474	0.9556	-0.0228 (-0.1139, 0.0683)	0.6204	0.9999
MiBP	3-Hydroxybutyrate	0.0131 (-0.2172, 0.2435)	0.9101	0.9785	0.0306 (-0.1982, 0.2594)	0.7909	0.9999
MiBP	3-Hydroxyisobutyrate	-0.0825 (-0.1965, 0.0315)	0.1539	0.9330	-0.1078 (-0.2150, -0.0006)	0.0488	0.9999
MiBP	3-Hydroxyisovalerate	-0.0591 (-0.1260, 0.0079)	0.0831	0.9330	-0.0429 (-0.1105, 0.0247)	0.2110	0.9999
MiBP	3-Methyl-2-oxo-butanoic acid	-0.0500 (-0.1165, 0.0164)	0.1383	0.9330	-0.0450 (-0.1150, 0.0250)	0.2050	0.9999
MiBP	Acetate	0.0334 (-0.0816, 0.1484)	0.5656	0.9556	0.0139 (-0.1033, 0.1310)	0.8147	0.9999
MiBP	Acetoacetate	-0.0263 (-0.1964, 0.1438)	0.7597	0.9701	-0.0129 (-0.1864, 0.1607)	0.8833	0.9999
MiBP	Acetone	0.0662 (-0.0584, 0.1908)	0.2942	0.9330	0.0680 (-0.0562, 0.1922)	0.2797	0.9999

		Total Effect			Direct Effect		
**Phthalate**	**Metabolite**	**Estimate (95% CI)**	***p***	**FDR *p***	**Estimate (95% CI)**	***p***	**FDR *p***
MiBP	Alanine	0.0055 (-0.0544, 0.0653)	0.8570	0.9701	-0.0081 (-0.0691, 0.0530)	0.7940	0.9999
MiBP	Arginine	-0.0769 (-0.1629, 0.0090)	0.0788	0.9330	-0.0680 (-0.1570, 0.0211)	0.1329	0.9999
MiBP	Asparagine	-0.0004 (-0.0840, 0.0831)	0.9923	0.9964	-0.0214 (-0.1035, 0.0607)	0.6057	0.9999
MiBP	Aspartate	-0.0412 (-0.1657, 0.0833)	0.5125	0.9556	-0.0570 (-0.1790, 0.0650)	0.3562	0.9999
MiBP	Betaine	-0.0240 (-0.0919, 0.0438)	0.4836	0.9556	-0.0069 (-0.0790, 0.0652)	0.8493	0.9999
MiBP	Carnitine	-0.0430 (-0.1123, 0.0264)	0.2216	0.9330	-0.0451 (-0.1166, 0.0264)	0.2134	0.9999
MiBP	Choline	-0.0246 (-0.1023, 0.0531)	0.5316	0.9556	-0.0186 (-0.0978, 0.0606)	0.6419	0.9999
MiBP	Citrate	-0.0365 (-0.1119, 0.0389)	0.3389	0.9330	-0.0203 (-0.0949, 0.0543)	0.5904	0.9999
MiBP	Creatine	-0.0325 (-0.1549, 0.0899)	0.5994	0.9623	-0.0425 (-0.1664, 0.0815)	0.4979	0.9999
MiBP	Creatinine	-0.0107 (-0.0696, 0.0482)	0.7195	0.9701	-0.0107 (-0.0713, 0.0500)	0.7278	0.9999
MiBP	Cystine	-0.0559 (-0.1606, 0.0487)	0.2915	0.9330	-0.0442 (-0.1539, 0.0654)	0.4253	0.9999
MiBP	Dimethyl sulfone	-0.0115 (-0.1421, 0.1190)	0.8611	0.9701	-0.0526 (-0.1786, 0.0735)	0.4101	0.9999
MiBP	Formate	-0.0251 (-0.0956, 0.0454)	0.4809	0.9556	-0.0449 (-0.1161, 0.0263)	0.2140	0.9999
MiBP	Glucose	-0.0177 (-0.0917, 0.0562)	0.6351	0.9677	-0.0120 (-0.0876, 0.0635)	0.7521	0.9999
MiBP	Glutamate	0.0063 (-0.1167, 0.1293)	0.9194	0.9823	0.0064 (-0.1182, 0.1310)	0.9187	0.9999
MiBP	Glutamine	-0.0122 (-0.0621, 0.0377)	0.6288	0.9677	-0.0117 (-0.0631, 0.0398)	0.6540	0.9999
MiBP	Glycine	0.0004 (-0.0753, 0.0761)	0.9924	0.9964	-0.0066 (-0.0840, 0.0708)	0.8667	0.9999
MiBP	Isoleucine	-0.0672 (-0.1633, 0.0290)	0.1688	0.9330	-0.0735 (-0.1712, 0.0242)	0.1387	0.9999
MiBP	Lactate	0.0589 (-0.0260, 0.1438)	0.1717	0.9330	0.0487 (-0.0375, 0.1349)	0.2645	0.9999
MiBP	Leucine	-0.0699 (-0.1518, 0.0121)	0.0937	0.9330	-0.0758 (-0.1581, 0.0064)	0.0704	0.9999
MiBP	Lysine	-0.0522 (-0.1356, 0.0311)	0.2164	0.9330	-0.0535 (-0.1381, 0.0311)	0.2122	0.9999
MiBP	Methanol	-0.1102 (-0.2979, 0.0775)	0.2468	0.9330	-0.1646 (-0.3537, 0.0246)	0.0874	0.9999
MiBP	Methionine	-0.0489 (-0.1291, 0.0313)	0.2288	0.9330	-0.0668 (-0.1473, 0.0136)	0.1026	0.9999
MiBP	myo-Inositol	0.0257 (-0.0469, 0.0983)	0.4839	0.9556	0.0094 (-0.0660, 0.0849)	0.8045	0.9999
MiBP	N,N-Dimethylglycine	-0.0472 (-0.1128, 0.0184)	0.1563	0.9330	-0.0453 (-0.1134, 0.0228)	0.1899	0.9999
MiBP	O-Acetylcarnitine	-0.0078 (-0.0799, 0.0643)	0.8309	0.9701	-0.0085 (-0.0811, 0.0641)	0.8175	0.9999
MiBP	Ornithine	0.0122 (-0.0941, 0.1184)	0.8206	0.9701	-0.0235 (-0.1291, 0.0821)	0.6596	0.9999
MiBP	Phenylalanine	-0.0601 (-0.1294, 0.0092)	0.0884	0.9330	-0.0708 (-0.1408, -0.0008)	0.0474	0.9999
MiBP	Proline	-0.0105 (-0.0946, 0.0736)	0.8051	0.9701	-0.0117 (-0.0975, 0.0742)	0.7879	0.9999
MiBP	Pyruvate	0.1549 (0.0057, 0.3041)	0.0420	0.9330	0.1229 (-0.0272, 0.2730)	0.1073	0.9999
MiBP	Serine	-0.0130 (-0.0883, 0.0622)	0.7318	0.9701	-0.0261 (-0.1003, 0.0482)	0.4874	0.9999
MiBP	Taurine	0.0461 (-0.0890, 0.1812)	0.5002	0.9556	0.0243 (-0.1084, 0.1571)	0.7166	0.9999
MiBP	Threonine	-0.0347 (-0.1531, 0.0837)	0.5625	0.9556	-0.0377 (-0.1594, 0.0840)	0.5402	0.9999
MiBP	Tyrosine	-0.0360 (-0.1197, 0.0477)	0.3950	0.9389	-0.0548 (-0.1381, 0.0285)	0.1945	0.9999
MiBP	Urea	-0.0621 (-0.1618, 0.0377)	0.2202	0.9330	-0.0883 (-0.1855, 0.0089)	0.0744	0.9999
MiBP	Valine	-0.0636 (-0.1395, 0.0123)	0.0997	0.9330	-0.0742 (-0.1504, 0.0020)	0.0562	0.9999
MNP	2-Hydroxybutyrate	-0.0620 (-0.1793, 0.0552)	0.2963	0.9330	-0.0162 (-0.1439, 0.1115)	0.8015	0.9999
MNP	2-Hydroxyisobutyrate	-0.0402 (-0.0994, 0.0190)	0.1810	0.9330	-0.0291 (-0.0943, 0.0360)	0.3773	0.9999
MNP	2-Hydroxyisovalerate	-0.0282 (-0.0953, 0.0388)	0.4054	0.9426	-0.0198 (-0.0916, 0.0521)	0.5862	0.9999
MNP	2-Oxoglutarate	-0.0383 (-0.1104, 0.0339)	0.2952	0.9330	-0.0207 (-0.0973, 0.0560)	0.5937	0.9999
MNP	2-Oxoisocaproate	-0.0867 (-0.1618, -0.0116)	0.0242	0.9330	-0.0741 (-0.1566, 0.0085)	0.0781	0.9999
MNP	3-Hydroxybutyrate	-0.2142 (-0.4124, -0.0160)	0.0345	0.9330	-0.1291 (-0.3380, 0.0799)	0.2232	0.9999
MNP	3-Hydroxyisobutyrate	0.0010 (-0.1004, 0.1025)	0.9837	0.9964	0.0520 (-0.0481, 0.1521)	0.3053	0.9999
MNP	3-Hydroxyisovalerate	-0.0264 (-0.0860, 0.0333)	0.3826	0.9350	-0.0150 (-0.0777, 0.0476)	0.6353	0.9999
MNP	3-Methyl-2-oxo-butanoic acid	-0.0439 (-0.1024, 0.0146)	0.1394	0.9330	-0.0379 (-0.1024, 0.0266)	0.2466	0.9999
MNP	Acetate	0.0547 (-0.0461, 0.1555)	0.2840	0.9330	0.0280 (-0.0796, 0.1357)	0.6063	0.9999
MNP	Acetoacetate	-0.1274 (-0.2750, 0.0202)	0.0899	0.9330	-0.0940 (-0.2526, 0.0645)	0.2421	0.9999
MNP	Acetone	0.0267 (-0.0835, 0.1369)	0.6316	0.9677	0.0656 (-0.0486, 0.1798)	0.2573	0.9999
MNP	Alanine	0.0383 (-0.0139, 0.0905)	0.1482	0.9330	0.0360 (-0.0198, 0.0917)	0.2033	0.9999
MNP	Arginine	-0.0488 (-0.1251, 0.0274)	0.2069	0.9330	-0.0426 (-0.1250, 0.0399)	0.3079	0.9999
MNP	Asparagine	0.0731 (0.0010, 0.1451)	0.0471	0.9330	0.0626 (-0.0119, 0.1372)	0.0988	0.9999
MNP	Aspartate	0.0033 (-0.1065, 0.1132)	0.9520	0.9901	0.0499 (-0.0624, 0.1623)	0.3797	0.9999
MNP	Betaine	0.0137 (-0.0461, 0.0736)	0.6494	0.9690	0.0240 (-0.0422, 0.0901)	0.4736	0.9999
MNP	Carnitine	-0.0296 (-0.0908, 0.0317)	0.3402	0.9330	-0.0160 (-0.0822, 0.0502)	0.6320	0.9999
MNP	Choline	-0.0125 (-0.0810, 0.0560)	0.7179	0.9701	0.0122 (-0.0607, 0.0851)	0.7407	0.9999
MNP	Citrate	0.0240 (-0.0426, 0.0905)	0.4764	0.9556	0.0406 (-0.0277, 0.1088)	0.2413	0.9999
MNP	Creatine	0.0263 (-0.0815, 0.1341)	0.6295	0.9677	0.0414 (-0.0726, 0.1554)	0.4727	0.9999
MNP	Creatinine	0.0567 (0.0060, 0.1073)	0.0287	0.9330	0.0641 (0.0098, 0.1183)	0.0212	0.9999
MNP	Cystine	-0.0408 (-0.1331, 0.0515)	0.3825	0.9350	-0.0449 (-0.1458, 0.0559)	0.3788	0.9999
MNP	Dimethyl sulfone	0.0885 (-0.0251, 0.2021)	0.1254	0.9330	0.0557 (-0.0602, 0.1716)	0.3422	0.9999

		Total Effect			Direct Effect		
**Phthalate**	**Metabolite**	**Estimate (95% CI)**	***p***	**FDR *p***	**Estimate (95% CI)**	***p***	**FDR *p***
MNP	Formate	0.0083 (-0.0540, 0.0705)	0.7929	0.9701	0.0068 (-0.0593, 0.0728)	0.8387	0.9999
MNP	Glucose	0.0202 (-0.0449, 0.0852)	0.5394	0.9556	0.0118 (-0.0577, 0.0813)	0.7361	0.9999
MNP	Glutamate	-0.0192 (-0.1274, 0.0891)	0.7260	0.9701	0.0090 (-0.1057, 0.1237)	0.8767	0.9999
MNP	Glutamine	-0.0152 (-0.0591, 0.0287)	0.4933	0.9556	-0.0199 (-0.0672, 0.0273)	0.4042	0.9999
MNP	Glycine	0.0527 (-0.0131, 0.1186)	0.1149	0.9330	0.0555 (-0.0148, 0.1258)	0.1206	0.9999
MNP	Isoleucine	-0.0196 (-0.1050, 0.0657)	0.6491	0.9690	0.0004 (-0.0905, 0.0914)	0.9926	0.9999
MNP	Lactate	0.0674 (-0.0068, 0.1417)	0.0747	0.9330	0.0794 (0.0012, 0.1576)	0.0467	0.9999
MNP	Leucine	-0.0163 (-0.0894, 0.0568)	0.6587	0.9701	0.0082 (-0.0688, 0.0852)	0.8333	0.9999
MNP	Lysine	0.0177 (-0.0561, 0.0916)	0.6349	0.9677	0.0502 (-0.0276, 0.1280)	0.2031	0.9999
MNP	Methanol	-0.0308 (-0.1971, 0.1354)	0.7135	0.9701	-0.0203 (-0.1970, 0.1564)	0.8199	0.9999
MNP	Methionine	0.0819 (0.0127, 0.1511)	0.0208	0.9330	0.0938 (0.0211, 0.1664)	0.0120	0.9999
MNP	myo-Inositol	0.0520 (-0.0112, 0.1152)	0.1055	0.9330	0.0670 (-0.0011, 0.1351)	0.0538	0.9999
MNP	N,N-Dimethylglycine	0.0077 (-0.0506, 0.0661)	0.7926	0.9701	0.0203 (-0.0428, 0.0834)	0.5240	0.9999
MNP	O-Acetylcarnitine	-0.1000 (-0.1603, -0.0398)	0.0014	0.9228	-0.0684 (-0.1337, -0.0030)	0.0406	0.9999
MNP	Ornithine	0.0630 (-0.0297, 0.1557)	0.1804	0.9330	0.0845 (-0.0112, 0.1803)	0.0828	0.9999
MNP	Phenylalanine	0.0355 (-0.0260, 0.0971)	0.2544	0.9330	0.0502 (-0.0147, 0.1152)	0.1282	0.9999
MNP	Proline	0.0472 (-0.0262, 0.1206)	0.2052	0.9330	0.0610 (-0.0171, 0.1390)	0.1242	0.9999
MNP	Pyruvate	0.0602 (-0.0735, 0.1938)	0.3739	0.9330	0.0253 (-0.1146, 0.1653)	0.7200	0.9999
MNP	Serine	0.0315 (-0.0345, 0.0975)	0.3458	0.9330	0.0457 (-0.0221, 0.1136)	0.1841	0.9999
MNP	Taurine	0.0658 (-0.0526, 0.1843)	0.2729	0.9330	0.1186 (-0.0012, 0.2384)	0.0524	0.9999
MNP	Threonine	0.0487 (-0.0553, 0.1526)	0.3553	0.9330	0.0442 (-0.0677, 0.1561)	0.4347	0.9999
MNP	Tyrosine	0.0817 (0.0096, 0.1538)	0.0268	0.9330	0.0852 (0.0098, 0.1606)	0.0271	0.9999
MNP	Urea	0.0066 (-0.0819, 0.0951)	0.8824	0.9737	0.0108 (-0.0801, 0.1017)	0.8143	0.9999
MNP	Valine	0.0071 (-0.0607, 0.0749)	0.8360	0.9701	0.0264 (-0.0449, 0.0977)	0.4637	0.9999

Table S3: Estimates of the total and direct effects of a one quartile increase of each phthalate metabolite in the mixture on each serum metabolite. The estimate and 95% confidence interval (CI) for each serum metabolite were bootstrapped independently using quantile g-computation models. The total effect was adjusted for birth year, fasted time, gestational age at sample collection, homeownership, maternal education, and maternal race/ethnicity. The direct effect models were adjusted for birth year, birth year, fasted time, gestational age at sample collection, homeownership, maternal metabolic condition, and maternal race/ethnicity. All *p*-values were adjusted for false discovery rate (FDR) and both the original and FDR *p*-values are reported.

	Total Effect			Direct Effect		
**Metabolite**	**Estimate (95% CI)**	***p***	**FDR *p***	**Estimate (95% CI)**	***p***	**FDR *p***
2-Hydroxybutyrate	-0.0184 (-0.0833, 0.0465)	0.5792	0.9613	-0.0108 (-0.0746, 0.0531)	0.7414	0.9692
2-Hydroxyisobutyrate	-0.0173 (-0.0452, 0.0105)	0.2253	0.8688	-0.0192 (-0.0473, 0.0089)	0.1838	0.9692
2-Hydroxyisovalerate	-0.0008 (-0.0362, 0.0347)	0.9665	0.9790	-0.0022 (-0.0417, 0.0372)	0.9125	0.9692
2-Oxoglutarate	-0.0005 (-0.0394, 0.0383)	0.9790	0.9790	0.0048 (-0.0346, 0.0442)	0.8118	0.9692
2-Oxoisocaproate	-0.0256 (-0.0692, 0.0180)	0.2537	0.8688	-0.0230 (-0.0672, 0.0212)	0.3106	0.9692
3-Hydroxybutyrate	-0.0547 (-0.1673, 0.0580)	0.3444	0.8701	-0.0469 (-0.1564, 0.0627)	0.4041	0.9692
3-Hydroxyisobutyrate	-0.0142 (-0.0673, 0.0390)	0.6030	0.9613	-0.0135 (-0.0639, 0.0369)	0.6015	0.9692
3-Hydroxyisovalerate	-0.0154 (-0.0460, 0.0151)	0.3258	0.8688	-0.0125 (-0.0461, 0.0210)	0.4669	0.9692
3-Methyl-2-oxo-butanoic acid	-0.0228 (-0.0509, 0.0054)	0.1170	0.8688	-0.0225 (-0.0536, 0.0085)	0.1591	0.9692
Acetate	-0.0047 (-0.0612, 0.0518)	0.8712	0.9790	-0.0054 (-0.0650, 0.0543)	0.8606	0.9692
Acetoacetate	-0.0507 (-0.1420, 0.0406)	0.2793	0.8688	-0.0469 (-0.1389, 0.0451)	0.3203	0.9692
Acetone	0.0134 (-0.0454, 0.0722)	0.6566	0.9613	0.0166 (-0.0451, 0.0783)	0.6000	0.9692
Alanine	0.0210 (-0.0080, 0.0499)	0.1603	0.8688	0.0219 (-0.0093, 0.0531)	0.1721	0.9692
Arginine	-0.0158 (-0.0540, 0.0223)	0.4189	0.9091	-0.0184 (-0.0606, 0.0237)	0.3941	0.9692
Asparagine	0.0230 (-0.0157, 0.0617)	0.2478	0.8688	0.0184 (-0.0221, 0.0589)	0.3749	0.9692
Aspartate	-0.0030 (-0.0643, 0.0584)	0.9250	0.9790	-0.0047 (-0.0677, 0.0583)	0.8848	0.9692
Betaine	0.0018 (-0.0337, 0.0372)	0.9214	0.9790	0.0027 (-0.0341, 0.0395)	0.8849	0.9692
Carnitine	-0.0080 (-0.0437, 0.0277)	0.6609	0.9613	-0.0034 (-0.0394, 0.0326)	0.8539	0.9692
Choline	0.0096 (-0.0289, 0.0481)	0.6271	0.9613	0.0112 (-0.0316, 0.0540)	0.6102	0.9692
Citrate	-0.0020 (-0.0357, 0.0317)	0.9076	0.9790	0.0011 (-0.0346, 0.0367)	0.9539	0.9692
Creatine	0.0123 (-0.0507, 0.0753)	0.7031	0.9790	0.0073 (-0.0555, 0.0700)	0.8213	0.9692
Creatinine	0.0101 (-0.0169, 0.0371)	0.4645	0.9290	0.0116 (-0.0172, 0.0405)	0.4323	0.9692
Cystine	-0.0224 (-0.0716, 0.0267)	0.3730	0.8919	-0.0188 (-0.0690, 0.0314)	0.4640	0.9692
Dimethyl sulfone	0.0236 (-0.0355, 0.0828)	0.4356	0.9091	0.0019 (-0.0549, 0.0587)	0.9477	0.9692
Formate	-0.0122 (-0.0510, 0.0266)	0.5393	0.9613	-0.0136 (-0.0536, 0.0265)	0.5093	0.9692
Glucose	-0.0288 (-0.0643, 0.0068)	0.1166	0.8688	-0.0245 (-0.0598, 0.0109)	0.1784	0.9692
Glutamate	0.0025 (-0.0527, 0.0578)	0.9282	0.9790	0.0059 (-0.0536, 0.0653)	0.8472	0.9692
Glutamine	-0.0035 (-0.0290, 0.0220)	0.7904	0.9790	-0.0035 (-0.0311, 0.0241)	0.8029	0.9692
Glycine	0.0196 (-0.0178, 0.0570)	0.3076	0.8688	0.0183 (-0.0207, 0.0572)	0.3612	0.9692
Isoleucine	0.0010 (-0.0466, 0.0486)	0.9668	0.9790	0.0019 (-0.0504, 0.0542)	0.9438	0.9692
Lactate	0.0344 (-0.0069, 0.0758)	0.1065	0.8688	0.0372 (-0.0043, 0.0788)	0.0829	0.9692
Leucine	-0.0039 (-0.0446, 0.0368)	0.8530	0.9790	-0.0039 (-0.0466, 0.0389)	0.8595	0.9692
Lysine	0.0048 (-0.0419, 0.0515)	0.8414	0.9790	0.0053 (-0.0443, 0.0548)	0.8349	0.9692
Methanol	-0.1060 (-0.2141, 0.0021)	0.0580	0.8688	-0.1077 (-0.2145, -0.0008)	0.0516	0.9692
Methionine	0.0213 (-0.0198, 0.0624)	0.3127	0.8688	0.0171 (-0.0242, 0.0584)	0.4188	0.9692
myo-Inositol	-0.0162 (-0.0528, 0.0205)	0.3902	0.8919	-0.0179 (-0.0536, 0.0178)	0.3295	0.9692
N,N-Dimethylglycine	-0.0089 (-0.0396, 0.0218)	0.5722	0.9613	-0.0086 (-0.0397, 0.0224)	0.5871	0.9692
O-Acetylcarnitine	-0.0178 (-0.0512, 0.0157)	0.3008	0.8688	-0.0175 (-0.0493, 0.0143)	0.2846	0.9692
Ornithine	0.0296 (-0.0167, 0.0760)	0.2140	0.8688	0.0299 (-0.0166, 0.0764)	0.2112	0.9692
Phenylalanine	0.0092 (-0.0252, 0.0437)	0.6007	0.9613	0.0065 (-0.0284, 0.0415)	0.7148	0.9692
Proline	0.0312 (-0.0099, 0.0722)	0.1402	0.8688	0.0310 (-0.0120, 0.0741)	0.1617	0.9692
Pyruvate	0.0583 (-0.0161, 0.1328)	0.1285	0.8688	0.0620 (-0.0067, 0.1307)	0.0808	0.9692
Serine	0.0112 (-0.0213, 0.0438)	0.5005	0.9610	0.0096 (-0.0253, 0.0445)	0.5917	0.9692
Taurine	0.0420 (-0.0229, 0.1070)	0.2084	0.8688	0.0393 (-0.0246, 0.1033)	0.2312	0.9692
Threonine	0.0085 (-0.0508, 0.0678)	0.7798	0.9790	0.0038 (-0.0581, 0.0658)	0.9040	0.9692
Tyrosine	0.0211 (-0.0192, 0.0614)	0.3079	0.8688	0.0185 (-0.0233, 0.0603)	0.3887	0.9692
Urea	0.0062 (-0.0427, 0.0551)	0.8037	0.9790	-0.0028 (-0.0485, 0.0430)	0.9054	0.9692
Valine	0.0010 (-0.0368, 0.0387)	0.9604	0.9790	0.0008 (-0.0376, 0.0391)	0.9692	0.9692

Table S4: Permutational multivariate analysis of variance (PERMANOVA) to assess the association between the serum metabolome and neurodevelopmental outcome stratified by child of interest's sex. Serum metabolites were log_{10}-transformed. The marginal effects of terms were tested under 9999 permutations.

	**All Children**				**Females**				**Males**			
**Term**	**df**	***F***	R^2	***p***	**df**	***F***	R^2	***p***	**df**	***F***	R^2	***p***
Fasted time, minutes	1	2.22	0.0200	0.0217	1	2.25	0.0446	0.0236	1	2.05	0.0342	0.0273
Gestational age at serum collection, days	1	2.09	0.0189	0.0306	1	1.80	0.0357	0.0724	1	1.30	0.0217	0.1980
Birth year	1	1.91	0.0172	0.0490	1	3.62	0.0716	0.0016	1	0.69	0.0116	0.7554
Homeownership	1	1.20	0.0108	0.2577	1	1.77	0.0350	0.0752	1	0.59	0.0099	0.8642
Maternal race/ethnicity	1	1.24	0.0112	0.2330	1	1.08	0.0213	0.3392	1	0.60	0.0100	0.8574
Maternal metabolic condition	4	1.58	0.0569	0.0212	4	1.32	0.1044	0.1069	4	1.17	0.0780	0.2131
Neurodevelopmental Outcome	2	0.80	0.0144	0.7125	2	0.85	0.0337	0.6273	2	0.77	0.0257	0.7662
Residual	94		0.8492		34		0.6727		48		0.8023	
Total	105		1.0000		45		1.0000		59		1.0000	

Table S5: Associations between serum metabolites and neurodevelopmental outcomes. Single-response PERMANOVA was used to assess the association between each log_{10}-transformed serum metabolite concentration and neurodevelopmental outcome after adjustment for fasted time, gestational age at serum collection, birth year, homeownership status, maternal metabolic condition, and maternal race/ethnicity. Significance was assessed using permutation testing under 4999 permutations and *p*-values were corrected for false discovery rate (FDR).

Metabolite	F	R^2	*p*	FDR *p*
2-Hydroxybutyrate	1.81	0.0295	0.1675	0.9168
2-Hydroxyisobutyrate	1.22	0.0223	0.2865	0.9168
2-Hydroxyisovalerate	1.69	0.0322	0.2030	0.9168
2-Oxoglutarate	0.08	0.0013	0.9300	0.9935
2-Oxoisocaproate	1.93	0.0337	0.1625	0.9168
3-Hydroxybutyrate	1.55	0.0286	0.2225	0.9168
3-Hydroxyisobutyrate	0.87	0.0146	0.4200	0.9600
3-Hydroxyisovalerate	1.36	0.0251	0.2605	0.9168
3-Methyl-2-oxo-butanoic acid	0.95	0.0173	0.3890	0.9516
Acetate	1.98	0.0359	0.1480	0.9168
Acetoacetate	0.01	0.0002	0.9875	0.9935
Acetone	0.13	0.0025	0.8690	0.9935
Alanine	1.83	0.0334	0.1735	0.9168
Arginine	0.50	0.0094	0.6135	0.9935
Asparagine	0.62	0.0112	0.5565	0.9935
Aspartate	0.39	0.0063	0.6745	0.9935
Betaine	1.03	0.0184	0.3665	0.9516
Carnitine	3.07	0.0553	0.0390	0.9168
Choline	0.29	0.0044	0.7540	0.9935
Citrate	1.45	0.0227	0.2465	0.9168
Creatine	1.30	0.0250	0.2850	0.9168
Creatinine	1.05	0.0185	0.3670	0.9516
Cystine	3.22	0.0567	0.0380	0.9168
Dimethyl sulfone	0.02	0.0004	0.9815	0.9935
Formate	0.08	0.0016	0.9205	0.9935
Glucose	0.17	0.0029	0.8390	0.9935
Glutamate	0.52	0.0096	0.5990	0.9935
Glutamine	2.28	0.0448	0.1070	0.9168
Glycine	0.72	0.0138	0.5015	0.9935
Isoleucine	0.02	0.0004	0.9785	0.9935
Lactate	1.71	0.0281	0.1865	0.9168
Leucine	0.10	0.0019	0.8945	0.9935
Lysine	0.31	0.0059	0.7295	0.9935
Methanol	0.04	0.0007	0.9660	0.9935
Methionine	0.38	0.0072	0.6865	0.9935
myo-Inositol	0.15	0.0025	0.8645	0.9935
N,N-Dimethylglycine	1.03	0.0175	0.3740	0.9516
O-Acetylcarnitine	2.20	0.0359	0.1225	0.9168
Ornithine	0.15	0.0027	0.8545	0.9935
Phenylalanine	0.15	0.0027	0.8550	0.9935
Proline	0.01	0.0001	0.9935	0.9935
Pyruvate	0.51	0.0086	0.6055	0.9935
Serine	0.61	0.0108	0.5600	0.9935
Taurine	0.43	0.0070	0.6625	0.9935
Threonine	0.78	0.0153	0.4480	0.9775
Tyrosine	0.94	0.0174	0.3965	0.9516
Urea	0.54	0.0096	0.5925	0.9935
Valine	0.17	0.0032	0.8525	0.9935

Table S6: Estimates of the total and direct effects of each phthalate metabolite on each placental metabolite. The esitmated effect of each phthtalate metabolite on each placenta metabolite was modeled using multiple linear regression and the estimated coefficients and 95% confidence intervals (CI) are reported. The total effect was adjusted for birth year, maternal education, maternal race/ethnicity, and homeownership status. The direct effect models were adjusted for birth year, maternal education, maternal race/ethnicity, homeownership status, maternal metabolic condition, delivery mode, and gestational age at delivery. All *p*-values were adjusted for false discovery rate (FDR) and both the original and FDR *p*-values are reported.

		**Total Effect**			**Direct Effect**		
**Phthalate**	**Metabolite**	**Estimate (95% CI)**	**p**	**FDR p**	**Estimate (95% CI)**	**p**	**FDR p**
MBP	1,3-Dihydroxyacetone	-0.1729 (-0.4262, 0.0803)	0.1789	0.9986	-0.1804 (-0.4423, 0.0814)	0.1750	0.9993
MBP	2-Hydroxybutyrate	-0.0509 (-0.1762, 0.0744)	0.4229	0.9986	-0.0541 (-0.1829, 0.0746)	0.4067	0.9993
MBP	3-Hydroxybutyrate	0.1611 (-0.1205, 0.4426)	0.2597	0.9986	0.1358 (-0.1493, 0.4210)	0.3475	0.9993
MBP	4-Aminobutyrate	-0.0341 (-0.1924, 0.1242)	0.6708	0.9986	0.0057 (-0.1505, 0.1618)	0.9428	0.9993
MBP	4-Hydroxybutyrate	0.1234 (-0.2365, 0.4833)	0.4987	0.9986	0.1098 (-0.2630, 0.4826)	0.5608	0.9993
MBP	Acetate	-0.0286 (-0.2488, 0.1915)	0.7974	0.9986	-0.0102 (-0.2365, 0.2161)	0.9292	0.9993
MBP	Alanine	-0.0360 (-0.1350, 0.0631)	0.4738	0.9986	-0.0172 (-0.1169, 0.0825)	0.7335	0.9993
MBP	Arginine	-0.0250 (-0.1574, 0.1074)	0.7093	0.9986	-0.0228 (-0.1590, 0.1134)	0.7407	0.9993
MBP	Asparagine	-0.0260 (-0.1417, 0.0897)	0.6574	0.9986	-0.0173 (-0.1337, 0.0992)	0.7696	0.9993
MBP	Aspartate	-0.0353 (-0.1367, 0.0661)	0.4920	0.9986	-0.0431 (-0.1467, 0.0606)	0.4121	0.9993
MBP	Betaine	-0.0738 (-0.1883, 0.0407)	0.2043	0.9986	-0.0681 (-0.1854, 0.0492)	0.2527	0.9993
MBP	Carnitine	-0.0326 (-0.1329, 0.0677)	0.5212	0.9986	-0.0237 (-0.1272, 0.0798)	0.6513	0.9993
MBP	Choline	-0.0275 (-0.1351, 0.0800)	0.6132	0.9986	-0.0223 (-0.1332, 0.0885)	0.6907	0.9993
MBP	Creatine	-0.0323 (-0.1179, 0.0532)	0.4556	0.9986	-0.0257 (-0.1115, 0.0600)	0.5537	0.9993
MBP	Cystine	0.0259 (-0.1899, 0.2417)	0.8126	0.9986	0.0267 (-0.1964, 0.2497)	0.8133	0.9993
MBP	Ethanolamine	-0.0421 (-0.1540, 0.0697)	0.4573	0.9986	-0.0307 (-0.1453, 0.0839)	0.5970	0.9993
MBP	Formate	-0.0774 (-0.2015, 0.0467)	0.2196	0.9986	-0.0623 (-0.1873, 0.0626)	0.3255	0.9993
MBP	Fumarate	-0.0770 (-0.2349, 0.0809)	0.3365	0.9986	-0.0880 (-0.2508, 0.0747)	0.2864	0.9993
MBP	Glucitol	-0.0764 (-0.2017, 0.0489)	0.2297	0.9986	-0.0701 (-0.1987, 0.0585)	0.2824	0.9993
MBP	Glutamate	-0.0096 (-0.1036, 0.0845)	0.8408	0.9986	-0.0039 (-0.0998, 0.0921)	0.9364	0.9993
MBP	Glutamine	-0.0278 (-0.0923, 0.0367)	0.3956	0.9986	-0.0228 (-0.0867, 0.0411)	0.4813	0.9993
MBP	Glutathione	-0.0747 (-0.3161, 0.1667)	0.5414	0.9986	-0.0652 (-0.3102, 0.1798)	0.5995	0.9993
MBP	Glycerol	-0.0195 (-0.1440, 0.1049)	0.7566	0.9986	-0.0139 (-0.1421, 0.1143)	0.8305	0.9993
MBP	Glycine	-0.0178 (-0.1199, 0.0843)	0.7305	0.9986	-0.0090 (-0.1131, 0.0951)	0.8647	0.9993
MBP	Hypoxanthine	-0.0350 (-0.1308, 0.0608)	0.4707	0.9986	-0.0211 (-0.1184, 0.0762)	0.6681	0.9993
MBP	Inosine	-0.0115 (-0.2359, 0.2130)	0.9196	0.9986	-0.0296 (-0.2554, 0.1961)	0.7953	0.9993
MBP	Isoleucine	-0.0083 (-0.1207, 0.1042)	0.8845	0.9986	0.0044 (-0.1094, 0.1182)	0.9392	0.9993
MBP	Kynurenine	-0.0372 (-0.2102, 0.1359)	0.6716	0.9986	-0.0303 (-0.2076, 0.1470)	0.7356	0.9993
MBP	Lactate	0.0230 (-0.0498, 0.0958)	0.5333	0.9986	0.0339 (-0.0406, 0.1084)	0.3698	0.9993
MBP	Leucine	-0.0101 (-0.1266, 0.1064)	0.8641	0.9986	0.0040 (-0.1136, 0.1216)	0.9466	0.9993
MBP	Lysine	-0.0439 (-0.1757, 0.0880)	0.5117	0.9986	-0.0331 (-0.1665, 0.1003)	0.6241	0.9993
MBP	Methionine	0.0054 (-0.1182, 0.1290)	0.9314	0.9986	0.0173 (-0.1067, 0.1413)	0.7830	0.9993
MBP	myo-Inositol	0.0430 (-0.0682, 0.1541)	0.4458	0.9986	0.0291 (-0.0840, 0.1421)	0.6114	0.9993
MBP	N-Acetylneuraminate	-0.0749 (-0.2097, 0.0598)	0.2733	0.9986	-0.0747 (-0.2134, 0.0641)	0.2887	0.9993
MBP	NAD+	-0.0280 (-0.2838, 0.2278)	0.8287	0.9986	-0.0151 (-0.2770, 0.2469)	0.9095	0.9993
MBP	Niacinamide	0.0988 (-0.3800, 0.5776)	0.6838	0.9986	0.0720 (-0.4165, 0.5606)	0.7709	0.9993
MBP	O-Acetylcarnitine	0.0028 (-0.1155, 0.1210)	0.9628	0.9986	0.0082 (-0.1137, 0.1302)	0.8938	0.9993
MBP	O-Phosphocholine	-0.0410 (-0.3479, 0.2659)	0.7919	0.9986	-0.0154 (-0.3326, 0.3017)	0.9234	0.9993
MBP	O-Phosphoethanolamine	0.0433 (-0.1768, 0.2633)	0.6978	0.9986	0.0355 (-0.1878, 0.2588)	0.7535	0.9993
MBP	Ornithine	-0.0274 (-0.1984, 0.1437)	0.7521	0.9986	-0.0226 (-0.1974, 0.1522)	0.7987	0.9993
MBP	Pantothenate	-0.0647 (-0.2168, 0.0873)	0.4010	0.9986	-0.0616 (-0.2161, 0.0930)	0.4318	0.9993
MBP	Phenylalanine	-0.0109 (-0.1290, 0.1072)	0.8552	0.9986	0.0005 (-0.1190, 0.1200)	0.9937	0.9998
MBP	Proline	-0.0083 (-0.1095, 0.0929)	0.8711	0.9986	0.0035 (-0.0996, 0.1065)	0.9472	0.9993
MBP	Pyroglutamate	-0.1001 (-0.2389, 0.0386)	0.1556	0.9986	-0.1002 (-0.2436, 0.0432)	0.1689	0.9993
MBP	Serine	-0.0276 (-0.1479, 0.0926)	0.6501	0.9986	-0.0184 (-0.1397, 0.1028)	0.7638	0.9993
MBP	sn-Glycero-3-phosphocholine	-0.0864 (-0.2736, 0.1008)	0.3628	0.9986	-0.0589 (-0.2439, 0.1261)	0.5299	0.9993
MBP	Succinate	0.0382 (-0.1738, 0.2503)	0.7218	0.9986	0.0637 (-0.1531, 0.2805)	0.5617	0.9993
MBP	Taurine	-0.0187 (-0.1093, 0.0718)	0.6829	0.9986	-0.0090 (-0.1018, 0.0838)	0.8486	0.9993
MBP	Threonine	-0.0204 (-0.1296, 0.0889)	0.7126	0.9986	-0.0120 (-0.1214, 0.0973)	0.8280	0.9993

		Total Effect			Direct Effect		
**Phthalate**	**Metabolite**	**Estimate (95% CI)**	**p**	**FDR p**	**Estimate (95% CI)**	**p**	**FDR p**
MBP	Tryptophan	0.1301 (-0.0589, 0.3190)	0.1756	0.9986	0.1440 (-0.0475, 0.3354)	0.1392	0.9993
MBP	Tyrosine	-0.0120 (-0.1227, 0.0988)	0.8309	0.9986	-0.0019 (-0.1138, 0.1100)	0.9730	0.9993
MBP	Uracil	0.0033 (-0.1543, 0.1608)	0.9675	0.9986	0.0092 (-0.1523, 0.1707)	0.9106	0.9993
MBP	Uridine	-0.0821 (-0.2854, 0.1212)	0.4257	0.9986	-0.0905 (-0.2986, 0.1175)	0.3908	0.9993
MBP	Valine	-0.0145 (-0.1185, 0.0895)	0.7834	0.9986	-0.0030 (-0.1080, 0.1021)	0.9556	0.9993
MBzP	1,3-Dihydroxyacetone	-0.0190 (-0.1934, 0.1553)	0.8295	0.9986	-0.0188 (-0.2012, 0.1637)	0.8388	0.9993
MBzP	2-Hydroxybutyrate	-0.0986 (-0.1827, -0.0145)	0.0220	0.9986	-0.1046 (-0.1919, -0.0174)	0.0192	0.9993
MBzP	3-Hydroxybutyrate	-0.0889 (-0.2817, 0.1040)	0.3636	0.9986	-0.1286 (-0.3252, 0.0680)	0.1977	0.9993
MBzP	4-Aminobutyrate	0.0509 (-0.0570, 0.1589)	0.3523	0.9986	0.0919 (-0.0148, 0.1986)	0.0907	0.9993
MBzP	4-Hydroxybutyrate	0.1808 (-0.0637, 0.4252)	0.1458	0.9986	0.1698 (-0.0865, 0.4262)	0.1921	0.9993
MBzP	Acetate	-0.0302 (-0.1807, 0.1202)	0.6917	0.9986	-0.0190 (-0.1755, 0.1375)	0.8104	0.9993
MBzP	Alanine	-0.0024 (-0.0702, 0.0655)	0.9450	0.9986	0.0135 (-0.0555, 0.0824)	0.6994	0.9993
MBzP	Arginine	-0.0079 (-0.0985, 0.0827)	0.8633	0.9986	-0.0045 (-0.0988, 0.0897)	0.9247	0.9993
MBzP	Asparagine	0.0059 (-0.0733, 0.0851)	0.8835	0.9986	0.0108 (-0.0698, 0.0913)	0.7919	0.9993
MBzP	Aspartate	-0.0161 (-0.0854, 0.0533)	0.6476	0.9986	-0.0278 (-0.0995, 0.0439)	0.4444	0.9993
MBzP	Betaine	-0.0359 (-0.1144, 0.0427)	0.3677	0.9986	-0.0412 (-0.1225, 0.0400)	0.3168	0.9993
MBzP	Carnitine	-0.0328 (-0.1012, 0.0357)	0.3450	0.9986	-0.0286 (-0.1001, 0.0428)	0.4292	0.9993
MBzP	Choline	0.0222 (-0.0513, 0.0957)	0.5510	0.9986	0.0307 (-0.0459, 0.1072)	0.4292	0.9993
MBzP	Creatine	-0.0278 (-0.0862, 0.0307)	0.3489	0.9986	-0.0292 (-0.0884, 0.0299)	0.3298	0.9993
MBzP	Cystine	0.0511 (-0.0962, 0.1984)	0.4933	0.9986	0.0620 (-0.0919, 0.2159)	0.4265	0.9993
MBzP	Ethanolamine	0.0066 (-0.0700, 0.0833)	0.8643	0.9986	0.0170 (-0.0623, 0.0963)	0.6725	0.9993
MBzP	Formate	-0.0547 (-0.1395, 0.0302)	0.2047	0.9986	-0.0461 (-0.1325, 0.0402)	0.2925	0.9993
MBzP	Fumarate	-0.0603 (-0.1681, 0.0476)	0.2710	0.9986	-0.0751 (-0.1874, 0.0371)	0.1877	0.9993
MBzP	Glucitol	-0.0252 (-0.1112, 0.0608)	0.5632	0.9986	-0.0242 (-0.1135, 0.0651)	0.5923	0.9993
MBzP	Glutamate	0.0058 (-0.0585, 0.0702)	0.8577	0.9986	0.0050 (-0.0614, 0.0714)	0.8819	0.9993
MBzP	Glutamine	-0.0334 (-0.0773, 0.0105)	0.1345	0.9986	-0.0379 (-0.0816, 0.0059)	0.0892	0.9993
MBzP	Glutathione	-0.0601 (-0.2251, 0.1048)	0.4721	0.9986	-0.0699 (-0.2391, 0.0992)	0.4146	0.9993
MBzP	Glycerol	0.0303 (-0.0547, 0.1153)	0.4813	0.9986	0.0416 (-0.0468, 0.1300)	0.3530	0.9993
MBzP	Glycine	0.0007 (-0.0692, 0.0705)	0.9849	0.9986	0.0051 (-0.0669, 0.0771)	0.8883	0.9993
MBzP	Hypoxanthine	0.0101 (-0.0555, 0.0757)	0.7612	0.9986	0.0200 (-0.0473, 0.0872)	0.5580	0.9993
MBzP	Inosine	-0.0517 (-0.2049, 0.1015)	0.5056	0.9986	-0.0873 (-0.2427, 0.0680)	0.2680	0.9993
MBzP	Isoleucine	-0.0039 (-0.0808, 0.0730)	0.9192	0.9986	0.0072 (-0.0715, 0.0859)	0.8574	0.9993
MBzP	Kynurenine	-0.0347 (-0.1530, 0.0835)	0.5621	0.9986	-0.0364 (-0.1589, 0.0861)	0.5576	0.9993
MBzP	Lactate	0.0143 (-0.0355, 0.0641)	0.5698	0.9986	0.0215 (-0.0301, 0.0731)	0.4104	0.9993
MBzP	Leucine	0.0016 (-0.0781, 0.0813)	0.9690	0.9986	0.0139 (-0.0673, 0.0952)	0.7347	0.9993
MBzP	Lysine	-0.0136 (-0.1039, 0.0767)	0.7660	0.9986	-0.0018 (-0.0941, 0.0906)	0.9700	0.9993
MBzP	Methionine	0.0029 (-0.0816, 0.0874)	0.9454	0.9986	0.0161 (-0.0697, 0.1018)	0.7116	0.9993
MBzP	myo-Inositol	-0.0028 (-0.0789, 0.0734)	0.9429	0.9986	-0.0222 (-0.1003, 0.0560)	0.5756	0.9993
MBzP	N-Acetylneuraminate	-0.0265 (-0.1189, 0.0660)	0.5721	0.9986	-0.0252 (-0.1215, 0.0711)	0.6054	0.9993
MBzP	NAD+	-0.0938 (-0.2680, 0.0804)	0.2885	0.9986	-0.0986 (-0.2789, 0.0817)	0.2811	0.9993
MBzP	Niacinamide	0.0592 (-0.2682, 0.3867)	0.7210	0.9986	0.0408 (-0.2971, 0.3788)	0.8113	0.9993
MBzP	O-Acetylcarnitine	-0.0295 (-0.1101, 0.0512)	0.4714	0.9986	-0.0278 (-0.1120, 0.0564)	0.5145	0.9993
MBzP	O-Phosphocholine	-0.0965 (-0.3057, 0.1128)	0.3632	0.9986	-0.0849 (-0.3038, 0.1339)	0.4437	0.9993
MBzP	O-Phosphoethanolamine	-0.0030 (-0.1536, 0.1476)	0.9685	0.9986	-0.0238 (-0.1783, 0.1307)	0.7609	0.9993
MBzP	Ornithine	-0.0140 (-0.1310, 0.1029)	0.8126	0.9986	-0.0063 (-0.1273, 0.1146)	0.9174	0.9993
MBzP	Pantothenate	-0.0637 (-0.1673, 0.0400)	0.2265	0.9986	-0.0532 (-0.1599, 0.0535)	0.3258	0.9993
MBzP	Phenylalanine	0.0031 (-0.0777, 0.0839)	0.9394	0.9986	0.0133 (-0.0694, 0.0959)	0.7514	0.9993
MBzP	Proline	-0.0114 (-0.0806, 0.0577)	0.7440	0.9986	-0.0019 (-0.0732, 0.0694)	0.9583	0.9993
MBzP	Pyroglutamate	0.0025 (-0.0931, 0.0981)	0.9588	0.9986	0.0081 (-0.0918, 0.1081)	0.8722	0.9993
MBzP	Serine	0.0102 (-0.0720, 0.0925)	0.8059	0.9986	0.0192 (-0.0647, 0.1030)	0.6517	0.9993
MBzP	sn-Glycero-3-phosphocholine	-0.0108 (-0.1392, 0.1175)	0.8675	0.9986	0.0116 (-0.1166, 0.1397)	0.8585	0.9993
MBzP	Succinate	0.0133 (-0.1318, 0.1584)	0.8564	0.9986	0.0284 (-0.1217, 0.1784)	0.7089	0.9993
MBzP	Taurine	-0.0024 (-0.0644, 0.0596)	0.9389	0.9986	0.0002 (-0.0640, 0.0644)	0.9947	0.9998
MBzP	Threonine	0.0003 (-0.0744, 0.0751)	0.9930	0.9986	0.0073 (-0.0684, 0.0829)	0.8498	0.9993
MBzP	Tryptophan	0.0931 (-0.0360, 0.2222)	0.1560	0.9986	0.1035 (-0.0289, 0.2358)	0.1242	0.9993
MBzP	Tyrosine	0.0093 (-0.0664, 0.0850)	0.8089	0.9986	0.0181 (-0.0592, 0.0955)	0.6433	0.9993
MBzP	Uracil	0.0559 (-0.0513, 0.1632)	0.3041	0.9986	0.0743 (-0.0366, 0.1851)	0.1874	0.9993
MBzP	Uridine	0.0086 (-0.1308, 0.1480)	0.9029	0.9986	-0.0095 (-0.1539, 0.1348)	0.8963	0.9993
MBzP	Valine	-0.0105 (-0.0816, 0.0606)	0.7706	0.9986	-0.0011 (-0.0737, 0.0715)	0.9763	0.9993
MCNP	1,3-Dihydroxyacetone	0.0286 (-0.1487, 0.2059)	0.7501	0.9986	0.0392 (-0.1503, 0.2288)	0.6827	0.9993

		Total Effect			Direct Effect		
**Phthalate**	**Metabolite**	**Estimate (95% CI)**	**p**	**FDR p**	**Estimate (95% CI)**	**p**	**FDR p**
MCNP	2-Hydroxybutyrate	0.0433 (-0.0438, 0.1303)	0.3271	0.9986	0.0448 (-0.0477, 0.1372)	0.3397	0.9993
MCNP	3-Hydroxybutyrate	0.1380 (-0.0573, 0.3332)	0.1645	0.9986	0.1356 (-0.0687, 0.3399)	0.1913	0.9993
MCNP	4-Aminobutyrate	-0.0113 (-0.1214, 0.0989)	0.8396	0.9986	-0.0403 (-0.1523, 0.0718)	0.4781	0.9993
MCNP	4-Hydroxybutyrate	-0.0892 (-0.3395, 0.1610)	0.4816	0.9986	-0.0753 (-0.3433, 0.1927)	0.5789	0.9993
MCNP	Acetate	-0.0036 (-0.1568, 0.1495)	0.9627	0.9986	-0.0056 (-0.1683, 0.1570)	0.9454	0.9993
MCNP	Alanine	-0.0434 (-0.1120, 0.0252)	0.2128	0.9986	-0.0542 (-0.1252, 0.0168)	0.1336	0.9993
MCNP	Arginine	-0.0289 (-0.1209, 0.0631)	0.5347	0.9986	-0.0458 (-0.1434, 0.0518)	0.3544	0.9993
MCNP	Asparagine	-0.0435 (-0.1237, 0.0367)	0.2854	0.9986	-0.0654 (-0.1483, 0.0175)	0.1208	0.9993
MCNP	Aspartate	-0.0238 (-0.0943, 0.0467)	0.5047	0.9986	-0.0313 (-0.1058, 0.0432)	0.4075	0.9993
MCNP	Betaine	-0.0094 (-0.0895, 0.0707)	0.8162	0.9986	-0.0093 (-0.0941, 0.0755)	0.8285	0.9993
MCNP	Carnitine	-0.0097 (-0.0796, 0.0601)	0.7832	0.9986	-0.0269 (-0.1012, 0.0474)	0.4756	0.9993
MCNP	Choline	-0.0384 (-0.1129, 0.0362)	0.3105	0.9986	-0.0336 (-0.1131, 0.0459)	0.4042	0.9993
MCNP	Creatine	0.0001 (-0.0596, 0.0597)	0.9984	0.9986	0.0042 (-0.0575, 0.0659)	0.8930	0.9993
MCNP	Cystine	-0.0070 (-0.1571, 0.1430)	0.9261	0.9986	-0.0126 (-0.1730, 0.1477)	0.8762	0.9993
MCNP	Ethanolamine	-0.0263 (-0.1041, 0.0515)	0.5050	0.9986	-0.0308 (-0.1131, 0.0515)	0.4608	0.9993
MCNP	Formate	-0.0245 (-0.1113, 0.0622)	0.5768	0.9986	-0.0123 (-0.1025, 0.0778)	0.7874	0.9993
MCNP	Fumarate	-0.0245 (-0.1347, 0.0856)	0.6601	0.9986	-0.0341 (-0.1515, 0.0833)	0.5664	0.9993
MCNP	Glucitol	-0.0589 (-0.1459, 0.0281)	0.1827	0.9986	-0.0623 (-0.1545, 0.0299)	0.1833	0.9993
MCNP	Glutamate	-0.0106 (-0.0760, 0.0548)	0.7488	0.9986	-0.0178 (-0.0867, 0.0511)	0.6103	0.9993
MCNP	Glutamine	-0.0056 (-0.0506, 0.0395)	0.8074	0.9986	-0.0221 (-0.0679, 0.0238)	0.3424	0.9993
MCNP	Glutathione	-0.0708 (-0.2385, 0.0968)	0.4048	0.9986	-0.0991 (-0.2745, 0.0763)	0.2656	0.9993
MCNP	Glycerol	-0.0733 (-0.1589, 0.0124)	0.0929	0.9986	-0.0726 (-0.1639, 0.0186)	0.1176	0.9993
MCNP	Glycine	-0.0268 (-0.0977, 0.0440)	0.4550	0.9986	-0.0331 (-0.1077, 0.0415)	0.3813	0.9993
MCNP	Hypoxanthine	-0.0122 (-0.0789, 0.0546)	0.7188	0.9986	-0.0207 (-0.0905, 0.0492)	0.5596	0.9993
MCNP	Inosine	0.0932 (-0.0620, 0.2484)	0.2370	0.9986	0.0681 (-0.0937, 0.2300)	0.4063	0.9993
MCNP	Isoleucine	-0.0406 (-0.1184, 0.0373)	0.3048	0.9986	-0.0470 (-0.1284, 0.0343)	0.2548	0.9993
MCNP	Kynurenine	-0.0060 (-0.1264, 0.1145)	0.9220	0.9986	-0.0254 (-0.1528, 0.1020)	0.6934	0.9993
MCNP	Lactate	0.0004 (-0.0504, 0.0511)	0.9889	0.9986	-0.0032 (-0.0570, 0.0505)	0.9051	0.9993
MCNP	Leucine	-0.0489 (-0.1295, 0.0317)	0.2321	0.9986	-0.0571 (-0.1410, 0.0268)	0.1802	0.9993
MCNP	Lysine	-0.0757 (-0.1667, 0.0152)	0.1017	0.9986	-0.0826 (-0.1774, 0.0122)	0.0872	0.9993
MCNP	Methionine	-0.0291 (-0.1149, 0.0567)	0.5033	0.9986	-0.0336 (-0.1226, 0.0554)	0.4563	0.9993
MCNP	myo-Inositol	0.0304 (-0.0469, 0.1077)	0.4382	0.9986	0.0290 (-0.0521, 0.1102)	0.4802	0.9993
MCNP	N-Acetylneuraminate	-0.0917 (-0.1845, 0.0011)	0.0527	0.9986	-0.1020 (-0.2005, -0.0035)	0.0424	0.9993
MCNP	NAD+	-0.0020 (-0.1800, 0.1759)	0.9819	0.9986	-0.0351 (-0.2233, 0.1532)	0.7129	0.9993
MCNP	Niacinamide	0.0545 (-0.2786, 0.3875)	0.7468	0.9986	0.1110 (-0.2397, 0.4618)	0.5320	0.9993
MCNP	O-Acetylcarnitine	0.0063 (-0.0760, 0.0885)	0.8803	0.9986	0.0005 (-0.0872, 0.0881)	0.9919	0.9998
MCNP	O-Phosphocholine	0.0594 (-0.1538, 0.2727)	0.5822	0.9986	0.0213 (-0.2066, 0.2493)	0.8532	0.9993
MCNP	O-Phosphoethanolamine	-0.0358 (-0.1888, 0.1172)	0.6438	0.9986	-0.0642 (-0.2244, 0.0960)	0.4290	0.9993
MCNP	Ornithine	-0.0976 (-0.2153, 0.0202)	0.1036	0.9986	-0.0952 (-0.2197, 0.0293)	0.1327	0.9993
MCNP	Pantothenate	0.0618 (-0.0437, 0.1673)	0.2487	0.9986	0.0511 (-0.0599, 0.1621)	0.3639	0.9993
MCNP	Phenylalanine	-0.0700 (-0.1512, 0.0112)	0.0906	0.9986	-0.0785 (-0.1632, 0.0063)	0.0693	0.9993
MCNP	Proline	-0.0408 (-0.1108, 0.0292)	0.2507	0.9986	-0.0507 (-0.1242, 0.0228)	0.1747	0.9993
MCNP	Pyroglutamate	-0.0765 (-0.1729, 0.0198)	0.1183	0.9986	-0.0748 (-0.1778, 0.0283)	0.1533	0.9993
MCNP	Serine	-0.0459 (-0.1292, 0.0374)	0.2775	0.9986	-0.0606 (-0.1471, 0.0259)	0.1680	0.9993
MCNP	sn-Glycero-3-phosphocholine	0.0281 (-0.1024, 0.1586)	0.6710	0.9986	0.0362 (-0.0968, 0.1693)	0.5908	0.9993
MCNP	Succinate	0.0710 (-0.0760, 0.2180)	0.3412	0.9986	0.0618 (-0.0939, 0.2174)	0.4336	0.9993
MCNP	Taurine	0.0003 (-0.0627, 0.0634)	0.9917	0.9986	-0.0122 (-0.0789, 0.0545)	0.7180	0.9993
MCNP	Threonine	-0.0247 (-0.1006, 0.0512)	0.5210	0.9986	-0.0359 (-0.1143, 0.0425)	0.3660	0.9993
MCNP	Tryptophan	-0.0212 (-0.1536, 0.1111)	0.7512	0.9986	-0.0310 (-0.1697, 0.1078)	0.6596	0.9993
MCNP	Tyrosine	-0.0478 (-0.1244, 0.0288)	0.2191	0.9986	-0.0607 (-0.1404, 0.0190)	0.1344	0.9993
MCNP	Uracil	-0.0579 (-0.1670, 0.0512)	0.2957	0.9986	-0.0485 (-0.1643, 0.0672)	0.4083	0.9993
MCNP	Uridine	-0.0027 (-0.1445, 0.1390)	0.9699	0.9986	-0.0109 (-0.1609, 0.1391)	0.8862	0.9993
MCNP	Valine	-0.0377 (-0.1098, 0.0343)	0.3019	0.9986	-0.0451 (-0.1201, 0.0300)	0.2366	0.9993
MCOP	1,3-Dihydroxyacetone	0.0318 (-0.1210, 0.1847)	0.6809	0.9986	0.0261 (-0.1338, 0.1860)	0.7470	0.9993
MCOP	2-Hydroxybutyrate	-0.0055 (-0.0808, 0.0698)	0.8853	0.9986	-0.0118 (-0.0900, 0.0665)	0.7666	0.9993
MCOP	3-Hydroxybutyrate	0.0302 (-0.1394, 0.1998)	0.7249	0.9986	0.0454 (-0.1280, 0.2187)	0.6052	0.9993
MCOP	4-Aminobutyrate	-0.0216 (-0.1165, 0.0733)	0.6537	0.9986	-0.0337 (-0.1282, 0.0608)	0.4815	0.9993
MCOP	4-Hydroxybutyrate	0.0182 (-0.1979, 0.2344)	0.8677	0.9986	0.0238 (-0.2026, 0.2501)	0.8356	0.9993
MCOP	Acetate	0.0609 (-0.0707, 0.1925)	0.3617	0.9986	0.0719 (-0.0647, 0.2085)	0.2994	0.9993
MCOP	Alanine	-0.0038 (-0.0633, 0.0557)	0.8991	0.9986	-0.0011 (-0.0616, 0.0594)	0.9710	0.9993

		Total Effect			Direct Effect		
**Phthalate**	**Metabolite**	**Estimate (95% CI)**	**p**	**FDR p**	**Estimate (95% CI)**	**p**	**FDR p**
MCOP	Arginine	-0.0246 (-0.1039, 0.0547)	0.5408	0.9986	-0.0267 (-0.1092, 0.0558)	0.5222	0.9993
MCOP	Asparagine	-0.0306 (-0.0998, 0.0386)	0.3834	0.9986	-0.0294 (-0.0998, 0.0410)	0.4102	0.9993
MCOP	Aspartate	-0.0005 (-0.0614, 0.0604)	0.9877	0.9986	0.0018 (-0.0612, 0.0648)	0.9557	0.9993
MCOP	Betaine	-0.0176 (-0.0866, 0.0514)	0.6149	0.9986	-0.0147 (-0.0862, 0.0568)	0.6848	0.9993
MCOP	Carnitine	-0.0472 (-0.1069, 0.0125)	0.1199	0.9986	-0.0559 (-0.1179, 0.0061)	0.0766	0.9993
MCOP	Choline	-0.0211 (-0.0855, 0.0433)	0.5182	0.9986	-0.0161 (-0.0833, 0.0511)	0.6359	0.9993
MCOP	Creatine	0.0040 (-0.0474, 0.0554)	0.8786	0.9986	0.0069 (-0.0452, 0.0590)	0.7933	0.9993
MCOP	Cystine	0.0185 (-0.1108, 0.1479)	0.7773	0.9986	0.0180 (-0.1173, 0.1532)	0.7931	0.9993
MCOP	Ethanolamine	0.0070 (-0.0602, 0.0743)	0.8360	0.9986	0.0066 (-0.0629, 0.0762)	0.8504	0.9993
MCOP	Formate	0.0041 (-0.0708, 0.0790)	0.9139	0.9986	0.0173 (-0.0587, 0.0934)	0.6522	0.9993
MCOP	Fumarate	-0.0322 (-0.1271, 0.0627)	0.5030	0.9986	-0.0349 (-0.1339, 0.0641)	0.4865	0.9993
MCOP	Glucitol	-0.0279 (-0.1033, 0.0475)	0.4656	0.9986	-0.0204 (-0.0987, 0.0579)	0.6067	0.9993
MCOP	Glutamate	0.0124 (-0.0440, 0.0687)	0.6647	0.9986	0.0165 (-0.0417, 0.0746)	0.5762	0.9993
MCOP	Glutamine	-0.0019 (-0.0407, 0.0369)	0.9231	0.9986	-0.0084 (-0.0472, 0.0304)	0.6691	0.9993
MCOP	Glutathione	-0.0933 (-0.2374, 0.0507)	0.2021	0.9986	-0.1070 (-0.2545, 0.0404)	0.1533	0.9993
MCOP	Glycerol	-0.0309 (-0.1054, 0.0435)	0.4126	0.9986	-0.0263 (-0.1039, 0.0513)	0.5037	0.9993
MCOP	Glycine	-0.0048 (-0.0660, 0.0564)	0.8774	0.9986	0.0019 (-0.0612, 0.0650)	0.9527	0.9993
MCOP	Hypoxanthine	-0.0103 (-0.0679, 0.0472)	0.7233	0.9986	-0.0086 (-0.0676, 0.0504)	0.7741	0.9993
MCOP	Inosine	0.0281 (-0.1064, 0.1626)	0.6803	0.9986	0.0110 (-0.1259, 0.1479)	0.8739	0.9993
MCOP	Isoleucine	-0.0082 (-0.0757, 0.0592)	0.8094	0.9986	-0.0001 (-0.0691, 0.0689)	0.9985	0.9998
MCOP	Kynurenine	-0.0856 (-0.1884, 0.0171)	0.1016	0.9986	-0.0899 (-0.1962, 0.0164)	0.0965	0.9993
MCOP	Lactate	0.0115 (-0.0322, 0.0551)	0.6042	0.9986	0.0105 (-0.0348, 0.0558)	0.6471	0.9993
MCOP	Leucine	-0.0173 (-0.0871, 0.0526)	0.6255	0.9986	-0.0090 (-0.0803, 0.0623)	0.8026	0.9993
MCOP	Lysine	-0.0150 (-0.0942, 0.0642)	0.7089	0.9986	-0.0037 (-0.0847, 0.0773)	0.9285	0.9993
MCOP	Methionine	0.0008 (-0.0733, 0.0749)	0.9821	0.9986	0.0115 (-0.0637, 0.0867)	0.7620	0.9993
MCOP	myo-Inositol	0.0365 (-0.0300, 0.1029)	0.2799	0.9986	0.0383 (-0.0300, 0.1066)	0.2687	0.9993
MCOP	N-Acetylneuraminate	-0.0718 (-0.1520, 0.0084)	0.0788	0.9986	-0.0680 (-0.1516, 0.0157)	0.1103	0.9993
MCOP	NAD+	-0.0368 (-0.1901, 0.1165)	0.6354	0.9986	-0.0591 (-0.2176, 0.0994)	0.4619	0.9993
MCOP	Niacinamide	0.0646 (-0.2225, 0.3517)	0.6569	0.9986	0.0651 (-0.2310, 0.3613)	0.6639	0.9993
MCOP	O-Acetylcarnitine	-0.0320 (-0.1027, 0.0386)	0.3713	0.9986	-0.0347 (-0.1084, 0.0389)	0.3524	0.9993
MCOP	O-Phosphocholine	-0.1533 (-0.3354, 0.0288)	0.0981	0.9986	-0.1815 (-0.3710, 0.0080)	0.0604	0.9993
MCOP	O-Phosphoethanolamine	-0.1181 (-0.2485, 0.0123)	0.0754	0.9986	-0.1197 (-0.2534, 0.0140)	0.0789	0.9993
MCOP	Ornithine	-0.0052 (-0.1078, 0.0974)	0.9197	0.9986	0.0083 (-0.0977, 0.1144)	0.8764	0.9993
MCOP	Pantothenate	0.0061 (-0.0853, 0.0975)	0.8952	0.9986	-0.0133 (-0.1072, 0.0806)	0.7794	0.9993
MCOP	Phenylalanine	-0.0317 (-0.1023, 0.0390)	0.3768	0.9986	-0.0234 (-0.0958, 0.0489)	0.5223	0.9993
MCOP	Proline	-0.0092 (-0.0699, 0.0514)	0.7642	0.9986	-0.0058 (-0.0683, 0.0567)	0.8546	0.9993
MCOP	Pyroglutamate	0.0019 (-0.0819, 0.0858)	0.9637	0.9986	0.0042 (-0.0834, 0.0919)	0.9237	0.9993
MCOP	Serine	-0.0103 (-0.0824, 0.0619)	0.7788	0.9986	-0.0038 (-0.0774, 0.0697)	0.9186	0.9993
MCOP	sn-Glycero-3-phosphocholine	-0.0436 (-0.1559, 0.0688)	0.4442	0.9986	-0.0299 (-0.1421, 0.0824)	0.5990	0.9993
MCOP	Succinate	0.1120 (-0.0137, 0.2377)	0.0803	0.9986	0.1124 (-0.0176, 0.2425)	0.0896	0.9993
MCOP	Taurine	0.0032 (-0.0511, 0.0576)	0.9068	0.9986	0.0008 (-0.0555, 0.0571)	0.9783	0.9993
MCOP	Threonine	-0.0288 (-0.0941, 0.0366)	0.3852	0.9986	-0.0224 (-0.0887, 0.0438)	0.5034	0.9993
MCOP	Tryptophan	0.0985 (-0.0143, 0.2113)	0.0863	0.9986	0.1134 (-0.0020, 0.2287)	0.0540	0.9993
MCOP	Tyrosine	-0.0085 (-0.0749, 0.0579)	0.7998	0.9986	-0.0022 (-0.0701, 0.0657)	0.9491	0.9993
MCOP	Uracil	-0.0141 (-0.1085, 0.0803)	0.7683	0.9986	-0.0066 (-0.1045, 0.0913)	0.8943	0.9993
MCOP	Uridine	0.0738 (-0.0478, 0.1953)	0.2320	0.9986	0.0869 (-0.0387, 0.2125)	0.1732	0.9993
MCOP	Valine	-0.0117 (-0.0740, 0.0506)	0.7110	0.9986	-0.0044 (-0.0680, 0.0593)	0.8924	0.9993
MCPP	1,3-Dihydroxyacetone	-0.0100 (-0.2282, 0.2082)	0.9280	0.9986	-0.0065 (-0.2363, 0.2232)	0.9553	0.9993
MCPP	2-Hydroxybutyrate	-0.0284 (-0.1357, 0.0789)	0.6014	0.9986	-0.0296 (-0.1419, 0.0827)	0.6028	0.9993
MCPP	3-Hydroxybutyrate	0.0678 (-0.1740, 0.3096)	0.5801	0.9986	0.0618 (-0.1872, 0.3108)	0.6241	0.9993
MCPP	4-Aminobutyrate	-0.0326 (-0.1680, 0.1028)	0.6349	0.9986	-0.0398 (-0.1756, 0.0960)	0.5628	0.9993
MCPP	4-Hydroxybutyrate	-0.0170 (-0.3255, 0.2914)	0.9131	0.9986	-0.0095 (-0.3346, 0.3156)	0.9540	0.9993
MCPP	Acetate	-0.0052 (-0.1936, 0.1832)	0.9569	0.9986	0.0120 (-0.1851, 0.2090)	0.9044	0.9993
MCPP	Alanine	-0.0252 (-0.1101, 0.0596)	0.5570	0.9986	-0.0201 (-0.1069, 0.0667)	0.6473	0.9993
MCPP	Arginine	-0.0288 (-0.1421, 0.0844)	0.6155	0.9986	-0.0348 (-0.1533, 0.0837)	0.5617	0.9993
MCPP	Asparagine	-0.0316 (-0.1306, 0.0673)	0.5279	0.9986	-0.0394 (-0.1406, 0.0617)	0.4419	0.9993
MCPP	Aspartate	0.0183 (-0.0685, 0.1051)	0.6773	0.9986	0.0132 (-0.0772, 0.1037)	0.7729	0.9993
MCPP	Betaine	-0.0591 (-0.1572, 0.0389)	0.2348	0.9986	-0.0668 (-0.1688, 0.0351)	0.1969	0.9993
MCPP	Carnitine	-0.0135 (-0.0995, 0.0724)	0.7557	0.9986	-0.0255 (-0.1156, 0.0645)	0.5756	0.9993
MCPP	Choline	-0.0346 (-0.1265, 0.0573)	0.4577	0.9986	-0.0228 (-0.1193, 0.0737)	0.6407	0.9993

		Total Effect			Direct Effect		
**Phthalate**	**Metabolite**	**Estimate (95% CI)**	**p**	**FDR p**	**Estimate (95% CI)**	**p**	**FDR p**
MCPP	Creatine	-0.0436 (-0.1165, 0.0293)	0.2390	0.9986	-0.0451 (-0.1194, 0.0293)	0.2326	0.9993
MCPP	Cystine	-0.0196 (-0.2043, 0.1650)	0.8336	0.9986	-0.0175 (-0.2118, 0.1767)	0.8585	0.9993
MCPP	Ethanolamine	-0.0021 (-0.0980, 0.0938)	0.9658	0.9986	-0.0001 (-0.1000, 0.0998)	0.9984	0.9998
MCPP	Formate	-0.0372 (-0.1438, 0.0695)	0.4914	0.9986	-0.0151 (-0.1243, 0.0941)	0.7844	0.9993
MCPP	Fumarate	0.0247 (-0.1108, 0.1603)	0.7185	0.9986	0.0122 (-0.1302, 0.1546)	0.8654	0.9993
MCPP	Glucitol	-0.0270 (-0.1347, 0.0807)	0.6208	0.9986	-0.0177 (-0.1302, 0.0948)	0.7560	0.9993
MCPP	Glutamate	0.0227 (-0.0577, 0.1031)	0.5769	0.9986	0.0253 (-0.0582, 0.1087)	0.5501	0.9993
MCPP	Glutamine	0.0062 (-0.0492, 0.0616)	0.8251	0.9986	-0.0082 (-0.0639, 0.0476)	0.7721	0.9993
MCPP	Glutathione	-0.0856 (-0.2919, 0.1208)	0.4134	0.9986	-0.1040 (-0.3167, 0.1088)	0.3352	0.9993
MCPP	Glycerol	-0.0567 (-0.1628, 0.0494)	0.2923	0.9986	-0.0443 (-0.1557, 0.0670)	0.4322	0.9993
MCPP	Glycine	0.0019 (-0.0854, 0.0893)	0.9651	0.9986	0.0066 (-0.0841, 0.0973)	0.8857	0.9993
MCPP	Hypoxanthine	-0.0116 (-0.0937, 0.0705)	0.7805	0.9986	-0.0093 (-0.0941, 0.0755)	0.8286	0.9993
MCPP	Inosine	0.0897 (-0.1017, 0.2810)	0.3557	0.9986	0.0536 (-0.1428, 0.2500)	0.5898	0.9993
MCPP	Isoleucine	-0.0245 (-0.1206, 0.0717)	0.6152	0.9986	-0.0162 (-0.1153, 0.0828)	0.7463	0.9993
MCPP	Kynurenine	-0.0431 (-0.1911, 0.1049)	0.5655	0.9986	-0.0584 (-0.2125, 0.0957)	0.4546	0.9993
MCPP	Lactate	0.0036 (-0.0588, 0.0660)	0.9095	0.9986	0.0045 (-0.0606, 0.0697)	0.8907	0.9993
MCPP	Leucine	-0.0450 (-0.1444, 0.0544)	0.3721	0.9986	-0.0382 (-0.1404, 0.0639)	0.4605	0.9993
MCPP	Lysine	-0.0418 (-0.1546, 0.0710)	0.4646	0.9986	-0.0336 (-0.1497, 0.0826)	0.5683	0.9993
MCPP	Methionine	-0.0286 (-0.1342, 0.0770)	0.5928	0.9986	-0.0148 (-0.1228, 0.0931)	0.7860	0.9993
MCPP	myo-Inositol	0.0301 (-0.0651, 0.1253)	0.5325	0.9986	0.0248 (-0.0736, 0.1232)	0.6188	0.9993
MCPP	N-Acetylneuraminate	-0.0540 (-0.1695, 0.0614)	0.3561	0.9986	-0.0491 (-0.1701, 0.0719)	0.4235	0.9993
MCPP	NAD+	0.0034 (-0.2155, 0.2223)	0.9757	0.9986	-0.0284 (-0.2565, 0.1997)	0.8058	0.9993
MCPP	Niacinamide	0.2743 (-0.1328, 0.6814)	0.1847	0.9986	0.2971 (-0.1251, 0.7192)	0.1661	0.9993
MCPP	O-Acetylcarnitine	-0.0134 (-0.1145, 0.0878)	0.7942	0.9986	-0.0211 (-0.1272, 0.0850)	0.6944	0.9993
MCPP	O-Phosphocholine	-0.0870 (-0.3493, 0.1752)	0.5124	0.9986	-0.1304 (-0.4056, 0.1447)	0.3498	0.9993
MCPP	O-Phosphoethanolamine	-0.0944 (-0.2821, 0.0933)	0.3214	0.9986	-0.1251 (-0.3183, 0.0681)	0.2023	0.9993
MCPP	Ornithine	-0.0421 (-0.1883, 0.1042)	0.5702	0.9986	-0.0264 (-0.1785, 0.1258)	0.7322	0.9993
MCPP	Pantothenate	0.0081 (-0.1223, 0.1386)	0.9019	0.9986	-0.0124 (-0.1473, 0.1225)	0.8557	0.9993
MCPP	Phenylalanine	-0.0639 (-0.1644, 0.0365)	0.2103	0.9986	-0.0554 (-0.1590, 0.0482)	0.2915	0.9993
MCPP	Proline	-0.0213 (-0.1078, 0.0652)	0.6273	0.9986	-0.0166 (-0.1062, 0.0731)	0.7150	0.9993
MCPP	Pyroglutamate	-0.0273 (-0.1468, 0.0923)	0.6523	0.9986	-0.0184 (-0.1442, 0.1074)	0.7730	0.9993
MCPP	Serine	-0.0151 (-0.1181, 0.0878)	0.7715	0.9986	-0.0113 (-0.1169, 0.0943)	0.8327	0.9993
MCPP	sn-Glycero-3-phosphocholine	-0.0096 (-0.1702, 0.1511)	0.9063	0.9986	0.0094 (-0.1519, 0.1708)	0.9079	0.9993
MCPP	Succinate	0.0451 (-0.1363, 0.2265)	0.6235	0.9986	0.0471 (-0.1417, 0.2360)	0.6221	0.9993
MCPP	Taurine	0.0231 (-0.0543, 0.1006)	0.5559	0.9986	0.0171 (-0.0637, 0.0978)	0.6764	0.9993
MCPP	Threonine	-0.0268 (-0.1202, 0.0666)	0.5714	0.9986	-0.0233 (-0.1185, 0.0718)	0.6283	0.9993
MCPP	Tryptophan	0.0133 (-0.1495, 0.1762)	0.8714	0.9986	0.0242 (-0.1440, 0.1924)	0.7764	0.9993
MCPP	Tyrosine	-0.0188 (-0.1135, 0.0759)	0.6956	0.9986	-0.0158 (-0.1132, 0.0816)	0.7488	0.9993
MCPP	Uracil	-0.0530 (-0.1875, 0.0814)	0.4366	0.9986	-0.0333 (-0.1738, 0.1072)	0.6397	0.9993
MCPP	Uridine	0.0830 (-0.0908, 0.2568)	0.3463	0.9986	0.0839 (-0.0972, 0.2650)	0.3607	0.9993
MCPP	Valine	-0.0244 (-0.1133, 0.0645)	0.5881	0.9986	-0.0175 (-0.1089, 0.0739)	0.7057	0.9993
MECPP	1,3-Dihydroxyacetone	0.0174 (-0.1753, 0.2102)	0.8581	0.9986	0.0000 (-0.2046, 0.2046)	1.0000	1.0000
MECPP	2-Hydroxybutyrate	-0.0281 (-0.1228, 0.0667)	0.5592	0.9986	-0.0511 (-0.1508, 0.0486)	0.3122	0.9993
MECPP	3-Hydroxybutyrate	-0.0751 (-0.2885, 0.1384)	0.4877	0.9986	-0.0751 (-0.2967, 0.1465)	0.5036	0.9993
MECPP	4-Aminobutyrate	-0.0137 (-0.1334, 0.1060)	0.8206	0.9986	0.0053 (-0.1158, 0.1265)	0.9305	0.9993
MECPP	4-Hydroxybutyrate	-0.0563 (-0.3286, 0.2161)	0.6834	0.9986	-0.0947 (-0.3837, 0.1944)	0.5180	0.9993
MECPP	Acetate	-0.0275 (-0.1939, 0.1389)	0.7441	0.9986	-0.0241 (-0.1995, 0.1514)	0.7865	0.9993
MECPP	Alanine	-0.0022 (-0.0773, 0.0728)	0.9532	0.9986	0.0144 (-0.0629, 0.0917)	0.7134	0.9993
MECPP	Arginine	0.0007 (-0.0994, 0.1009)	0.9887	0.9986	0.0102 (-0.0955, 0.1158)	0.8492	0.9993
MECPP	Asparagine	-0.0041 (-0.0917, 0.0834)	0.9257	0.9986	0.0174 (-0.0729, 0.1077)	0.7039	0.9993
MECPP	Aspartate	-0.0002 (-0.0770, 0.0765)	0.9955	0.9986	0.0013 (-0.0793, 0.0820)	0.9736	0.9993
MECPP	Betaine	-0.0906 (-0.1762, -0.0050)	0.0382	0.9986	-0.0929 (-0.1829, -0.0030)	0.0429	0.9993
MECPP	Carnitine	-0.0629 (-0.1381, 0.0122)	0.0998	0.9986	-0.0625 (-0.1420, 0.0171)	0.1225	0.9993
MECPP	Choline	0.0004 (-0.0810, 0.0817)	0.9930	0.9986	0.0054 (-0.0806, 0.0914)	0.9012	0.9993
MECPP	Creatine	0.0130 (-0.0518, 0.0777)	0.6929	0.9986	0.0104 (-0.0562, 0.0770)	0.7586	0.9993
MECPP	Cystine	0.1060 (-0.0561, 0.2680)	0.1979	0.9986	0.1149 (-0.0569, 0.2867)	0.1880	0.9993
MECPP	Ethanolamine	-0.0129 (-0.0977, 0.0718)	0.7630	0.9986	-0.0031 (-0.0921, 0.0859)	0.9451	0.9993
MECPP	Formate	-0.0283 (-0.1226, 0.0660)	0.5535	0.9986	-0.0205 (-0.1178, 0.0767)	0.6768	0.9993
MECPP	Fumarate	-0.0150 (-0.1347, 0.1048)	0.8052	0.9986	-0.0157 (-0.1425, 0.1112)	0.8072	0.9993
MECPP	Glucitol	-0.0594 (-0.1541, 0.0352)	0.2165	0.9986	-0.0564 (-0.1561, 0.0433)	0.2648	0.9993

		Total Effect			Direct Effect		
**Phthalate**	**Metabolite**	**Estimate (95% CI)**	**p**	**FDR p**	**Estimate (95% CI)**	**p**	**FDR p**
MECPP	Glutamate	0.0016 (-0.0695, 0.0727)	0.9650	0.9986	0.0060 (-0.0684, 0.0804)	0.8731	0.9993
MECPP	Glutamine	-0.0059 (-0.0548, 0.0430)	0.8109	0.9986	-0.0053 (-0.0550, 0.0443)	0.8325	0.9993
MECPP	Glutathione	-0.1571 (-0.3377, 0.0235)	0.0877	0.9986	-0.1801 (-0.3675, 0.0074)	0.0596	0.9993
MECPP	Glycerol	0.0190 (-0.0750, 0.1131)	0.6896	0.9986	0.0281 (-0.0712, 0.1275)	0.5758	0.9993
MECPP	Glycine	0.0109 (-0.0662, 0.0881)	0.7801	0.9986	0.0258 (-0.0548, 0.1064)	0.5277	0.9993
MECPP	Hypoxanthine	-0.0074 (-0.0800, 0.0652)	0.8404	0.9986	0.0048 (-0.0708, 0.0803)	0.9009	0.9993
MECPP	Inosine	-0.0545 (-0.2239, 0.1149)	0.5253	0.9986	-0.0909 (-0.2652, 0.0835)	0.3042	0.9993
MECPP	Isoleucine	0.0041 (-0.0809, 0.0891)	0.9246	0.9986	0.0261 (-0.0620, 0.1143)	0.5583	0.9993
MECPP	Kynurenine	-0.1094 (-0.2389, 0.0201)	0.0971	0.9986	-0.0975 (-0.2340, 0.0389)	0.1595	0.9993
MECPP	Lactate	0.0126 (-0.0424, 0.0677)	0.6509	0.9986	0.0157 (-0.0422, 0.0736)	0.5925	0.9993
MECPP	Leucine	-0.0029 (-0.0910, 0.0852)	0.9480	0.9986	0.0210 (-0.0702, 0.1121)	0.6495	0.9993
MECPP	Lysine	-0.0082 (-0.1081, 0.0916)	0.8710	0.9986	0.0220 (-0.0815, 0.1255)	0.6747	0.9993
MECPP	Methionine	0.0324 (-0.0608, 0.1257)	0.4925	0.9986	0.0586 (-0.0371, 0.1542)	0.2277	0.9993
MECPP	myo-Inositol	0.0484 (-0.0353, 0.1322)	0.2546	0.9986	0.0355 (-0.0520, 0.1230)	0.4237	0.9993
MECPP	N-Acetylneuraminate	-0.0653 (-0.1670, 0.0364)	0.2062	0.9986	-0.0593 (-0.1668, 0.0483)	0.2776	0.9993
MECPP	NAD+	-0.1184 (-0.3106, 0.0739)	0.2254	0.9986	-0.1305 (-0.3323, 0.0713)	0.2029	0.9993
MECPP	Niacinamide	0.1477 (-0.2135, 0.5089)	0.4199	0.9986	0.1388 (-0.2394, 0.5170)	0.4689	0.9993
MECPP	O-Acetylcarnitine	-0.0805 (-0.1687, 0.0078)	0.0735	0.9986	-0.0726 (-0.1662, 0.0211)	0.1277	0.9993
MECPP	O-Phosphocholine	-0.2919 (-0.5181, -0.0656)	0.0119	0.9986	-0.2944 (-0.5346, -0.0542)	0.0167	0.9993
MECPP	O-Phosphoethanolamine	-0.1822 (-0.3456, -0.0189)	0.0290	0.9986	-0.1818 (-0.3519, -0.0116)	0.0365	0.9993
MECPP	Ornithine	-0.0129 (-0.1422, 0.1165)	0.8441	0.9986	0.0007 (-0.1349, 0.1363)	0.9920	0.9998
MECPP	Pantothenate	0.0375 (-0.0776, 0.1525)	0.5204	0.9986	0.0392 (-0.0808, 0.1591)	0.5192	0.9993
MECPP	Phenylalanine	0.0071 (-0.0822, 0.0964)	0.8754	0.9986	0.0266 (-0.0660, 0.1192)	0.5708	0.9993
MECPP	Proline	0.0304 (-0.0459, 0.1067)	0.4314	0.9986	0.0458 (-0.0337, 0.1253)	0.2559	0.9993
MECPP	Pyroglutamate	0.0581 (-0.0472, 0.1633)	0.2769	0.9986	0.0582 (-0.0534, 0.1698)	0.3037	0.9993
MECPP	Serine	0.0189 (-0.0720, 0.1098)	0.6820	0.9986	0.0428 (-0.0510, 0.1365)	0.3683	0.9993
MECPP	sn-Glycero-3-phosphocholine	-0.0757 (-0.2170, 0.0656)	0.2913	0.9986	-0.0312 (-0.1748, 0.1124)	0.6678	0.9993
MECPP	Succinate	-0.0432 (-0.2034, 0.1170)	0.5946	0.9986	-0.0393 (-0.2075, 0.1290)	0.6449	0.9993
MECPP	Taurine	-0.0262 (-0.0945, 0.0422)	0.4498	0.9986	-0.0225 (-0.0944, 0.0494)	0.5362	0.9993
MECPP	Threonine	0.0089 (-0.0737, 0.0915)	0.8315	0.9986	0.0324 (-0.0523, 0.1170)	0.4507	0.9993
MECPP	Tryptophan	0.1081 (-0.0345, 0.2507)	0.1360	0.9986	0.1235 (-0.0247, 0.2717)	0.1015	0.9993
MECPP	Tyrosine	0.0031 (-0.0806, 0.0869)	0.9409	0.9986	0.0256 (-0.0611, 0.1122)	0.5603	0.9993
MECPP	Uracil	0.0063 (-0.1128, 0.1253)	0.9173	0.9986	0.0205 (-0.1047, 0.1456)	0.7469	0.9993
MECPP	Uridine	0.0051 (-0.1490, 0.1591)	0.9482	0.9986	0.0096 (-0.1523, 0.1715)	0.9067	0.9993
MECPP	Valine	0.0103 (-0.0683, 0.0889)	0.7961	0.9986	0.0304 (-0.0508, 0.1117)	0.4597	0.9993
MEHHP	1,3-Dihydroxyacetone	0.0282 (-0.1431, 0.1994)	0.7453	0.9986	0.0110 (-0.1709, 0.1929)	0.9047	0.9993
MEHHP	2-Hydroxybutyrate	-0.0162 (-0.1005, 0.0681)	0.7044	0.9986	-0.0339 (-0.1227, 0.0549)	0.4510	0.9993
MEHHP	3-Hydroxybutyrate	-0.0785 (-0.2681, 0.1110)	0.4137	0.9986	-0.0779 (-0.2748, 0.1189)	0.4348	0.9993
MEHHP	4-Aminobutyrate	0.0043 (-0.1021, 0.1107)	0.9361	0.9986	0.0289 (-0.0787, 0.1364)	0.5960	0.9993
MEHHP	4-Hydroxybutyrate	-0.0028 (-0.2450, 0.2393)	0.9815	0.9986	-0.0336 (-0.2909, 0.2237)	0.7966	0.9993
MEHHP	Acetate	-0.0467 (-0.1944, 0.1009)	0.5323	0.9986	-0.0444 (-0.2002, 0.1115)	0.5741	0.9993
MEHHP	Alanine	0.0134 (-0.0532, 0.0800)	0.6916	0.9986	0.0299 (-0.0387, 0.0984)	0.3899	0.9993
MEHHP	Arginine	0.0002 (-0.0888, 0.0892)	0.9961	0.9986	0.0103 (-0.0836, 0.1043)	0.8278	0.9993
MEHHP	Asparagine	0.0083 (-0.0695, 0.0861)	0.8327	0.9986	0.0296 (-0.0505, 0.1098)	0.4654	0.9993
MEHHP	Aspartate	0.0010 (-0.0672, 0.0692)	0.9768	0.9986	0.0023 (-0.0693, 0.0740)	0.9489	0.9993
MEHHP	Betaine	-0.0702 (-0.1466, 0.0061)	0.0711	0.9986	-0.0714 (-0.1517, 0.0089)	0.0809	0.9993
MEHHP	Carnitine	-0.0518 (-0.1186, 0.0151)	0.1280	0.9986	-0.0489 (-0.1198, 0.0219)	0.1741	0.9993
MEHHP	Choline	0.0064 (-0.0659, 0.0787)	0.8606	0.9986	0.0103 (-0.0662, 0.0867)	0.7907	0.9993
MEHHP	Creatine	0.0190 (-0.0385, 0.0765)	0.5137	0.9986	0.0192 (-0.0399, 0.0784)	0.5207	0.9993
MEHHP	Cystine	0.1119 (-0.0317, 0.2555)	0.1254	0.9986	0.1245 (-0.0277, 0.2767)	0.1079	0.9993
MEHHP	Ethanolamine	-0.0014 (-0.0767, 0.0739)	0.9704	0.9986	0.0061 (-0.0730, 0.0852)	0.8792	0.9993
MEHHP	Formate	-0.0196 (-0.1034, 0.0641)	0.6434	0.9986	-0.0145 (-0.1009, 0.0720)	0.7408	0.9993
MEHHP	Fumarate	0.0098 (-0.0966, 0.1163)	0.8552	0.9986	0.0130 (-0.0997, 0.1257)	0.8199	0.9993
MEHHP	Glucitol	-0.0554 (-0.1394, 0.0286)	0.1945	0.9986	-0.0537 (-0.1422, 0.0349)	0.2326	0.9993
MEHHP	Glutamate	0.0033 (-0.0598, 0.0665)	0.9168	0.9986	0.0075 (-0.0586, 0.0737)	0.8217	0.9993
MEHHP	Glutamine	-0.0110 (-0.0544, 0.0325)	0.6180	0.9986	-0.0104 (-0.0545, 0.0337)	0.6404	0.9993
MEHHP	Glutathione	-0.1297 (-0.2904, 0.0311)	0.1129	0.9986	-0.1519 (-0.3188, 0.0149)	0.0739	0.9993
MEHHP	Glycerol	0.0294 (-0.0541, 0.1128)	0.4878	0.9986	0.0370 (-0.0512, 0.1252)	0.4077	0.9993
MEHHP	Glycine	0.0171 (-0.0514, 0.0856)	0.6214	0.9986	0.0319 (-0.0396, 0.1035)	0.3788	0.9993
MEHHP	Hypoxanthine	-0.0037 (-0.0682, 0.0607)	0.9086	0.9986	0.0072 (-0.0599, 0.0743)	0.8317	0.9993

		Total Effect			Direct Effect		
**Phthalate**	**Metabolite**	**Estimate (95% CI)**	**p**	**FDR p**	**Estimate (95% CI)**	**p**	**FDR p**
MEHHP	Inosine	-0.0729 (-0.2231, 0.0773)	0.3387	0.9986	-0.1043 (-0.2588, 0.0503)	0.1841	0.9993
MEHHP	Isoleucine	0.0158 (-0.0596, 0.0913)	0.6787	0.9986	0.0368 (-0.0414, 0.1150)	0.3536	0.9993
MEHHP	Kynurenine	-0.0958 (-0.2109, 0.0193)	0.1021	0.9986	-0.0853 (-0.2066, 0.0360)	0.1666	0.9993
MEHHP	Lactate	0.0158 (-0.0331, 0.0647)	0.5231	0.9986	0.0190 (-0.0325, 0.0705)	0.4661	0.9993
MEHHP	Leucine	0.0125 (-0.0657, 0.0908)	0.7518	0.9986	0.0355 (-0.0453, 0.1163)	0.3858	0.9993
MEHHP	Lysine	0.0118 (-0.0770, 0.1005)	0.7936	0.9986	0.0405 (-0.0513, 0.1323)	0.3843	0.9993
MEHHP	Methionine	0.0422 (-0.0404, 0.1249)	0.3138	0.9986	0.0661 (-0.0186, 0.1508)	0.1247	0.9993
MEHHP	myo-Inositol	0.0449 (-0.0295, 0.1193)	0.2344	0.9986	0.0350 (-0.0428, 0.1127)	0.3750	0.9993
MEHHP	N-Acetylneuraminate	-0.0492 (-0.1398, 0.0413)	0.2837	0.9986	-0.0432 (-0.1390, 0.0526)	0.3738	0.9993
MEHHP	NAD+	-0.1143 (-0.2850, 0.0564)	0.1874	0.9986	-0.1264 (-0.3056, 0.0527)	0.1649	0.9993
MEHHP	Niacinamide	0.0809 (-0.2406, 0.4024)	0.6193	0.9986	0.0541 (-0.2827, 0.3909)	0.7509	0.9993
MEHHP	O-Acetylcarnitine	-0.0820 (-0.1601, -0.0039)	0.0397	0.9986	-0.0771 (-0.1600, 0.0058)	0.0682	0.9993
MEHHP	O-Phosphocholine	-0.2578 (-0.4589, -0.0567)	0.0124	0.9986	-0.2557 (-0.4695, -0.0420)	0.0194	0.9993
MEHHP	O-Phosphoethanolamine	-0.1461 (-0.2917, -0.0005)	0.0492	0.9986	-0.1405 (-0.2925, 0.0114)	0.0696	0.9993
MEHHP	Ornithine	0.0188 (-0.0961, 0.1337)	0.7470	0.9986	0.0349 (-0.0855, 0.1553)	0.5673	0.9993
MEHHP	Pantothenate	0.0249 (-0.0774, 0.1273)	0.6303	0.9986	0.0290 (-0.0777, 0.1357)	0.5913	0.9993
MEHHP	Phenylalanine	0.0196 (-0.0596, 0.0989)	0.6246	0.9986	0.0384 (-0.0437, 0.1205)	0.3564	0.9993
MEHHP	Proline	0.0420 (-0.0256, 0.1095)	0.2209	0.9986	0.0580 (-0.0123, 0.1282)	0.1050	0.9993
MEHHP	Pyroglutamate	0.0541 (-0.0394, 0.1475)	0.2544	0.9986	0.0545 (-0.0446, 0.1537)	0.2783	0.9993
MEHHP	Serine	0.0273 (-0.0534, 0.1079)	0.5051	0.9986	0.0502 (-0.0329, 0.1334)	0.2341	0.9993
MEHHP	sn-Glycero-3-phosphocholine	-0.0597 (-0.1854, 0.0660)	0.3489	0.9986	-0.0261 (-0.1538, 0.1016)	0.6860	0.9993
MEHHP	Succinate	-0.0471 (-0.1894, 0.0951)	0.5132	0.9986	-0.0457 (-0.1952, 0.1038)	0.5460	0.9993
MEHHP	Taurine	-0.0273 (-0.0880, 0.0334)	0.3751	0.9986	-0.0235 (-0.0874, 0.0404)	0.4675	0.9993
MEHHP	Threonine	0.0251 (-0.0481, 0.0984)	0.4985	0.9986	0.0477 (-0.0272, 0.1227)	0.2095	0.9993
MEHHP	Tryptophan	0.0785 (-0.0485, 0.2056)	0.2236	0.9986	0.0962 (-0.0359, 0.2282)	0.1521	0.9993
MEHHP	Tyrosine	0.0181 (-0.0563, 0.0924)	0.6315	0.9986	0.0400 (-0.0369, 0.1168)	0.3051	0.9993
MEHHP	Uracil	0.0192 (-0.0865, 0.1250)	0.7194	0.9986	0.0297 (-0.0815, 0.1410)	0.5974	0.9993
MEHHP	Uridine	-0.0039 (-0.1408, 0.1330)	0.9553	0.9986	-0.0005 (-0.1444, 0.1434)	0.9944	0.9998
MEHHP	Valine	0.0227 (-0.0470, 0.0925)	0.5197	0.9986	0.0427 (-0.0293, 0.1147)	0.2428	0.9993
MEHP	1,3-Dihydroxyacetone	0.0260 (-0.1588, 0.2108)	0.7811	0.9986	0.0053 (-0.1872, 0.1978)	0.9567	0.9993
MEHP	2-Hydroxybutyrate	0.0042 (-0.0868, 0.0952)	0.9272	0.9986	-0.0059 (-0.1001, 0.0883)	0.9015	0.9993
MEHP	3-Hydroxybutyrate	-0.0483 (-0.2532, 0.1566)	0.6416	0.9986	-0.0304 (-0.2392, 0.1784)	0.7735	0.9993
MEHP	4-Aminobutyrate	-0.0026 (-0.1174, 0.1122)	0.9641	0.9986	0.0133 (-0.1006, 0.1272)	0.8172	0.9993
MEHP	4-Hydroxybutyrate	-0.0631 (-0.3242, 0.1980)	0.6335	0.9986	-0.0809 (-0.3529, 0.1911)	0.5568	0.9993
MEHP	Acetate	-0.1446 (-0.3022, 0.0130)	0.0717	0.9986	-0.1409 (-0.3040, 0.0223)	0.0899	0.9993
MEHP	Alanine	0.0027 (-0.0693, 0.0746)	0.9413	0.9986	0.0138 (-0.0589, 0.0866)	0.7074	0.9993
MEHP	Arginine	-0.0102 (-0.1063, 0.0858)	0.8333	0.9986	-0.0023 (-0.1017, 0.0972)	0.9642	0.9993
MEHP	Asparagine	-0.0084 (-0.0923, 0.0756)	0.8442	0.9986	0.0085 (-0.0764, 0.0935)	0.8428	0.9993
MEHP	Aspartate	-0.0197 (-0.0932, 0.0538)	0.5973	0.9986	-0.0167 (-0.0925, 0.0591)	0.6637	0.9993
MEHP	Betaine	-0.1159 (-0.1969, -0.0349)	0.0054	0.9986	-0.1129 (-0.1965, -0.0293)	0.0085	0.9993
MEHP	Carnitine	-0.0759 (-0.1475, -0.0043)	0.0378	0.9986	-0.0742 (-0.1486, 0.0002)	0.0506	0.9993
MEHP	Choline	-0.0149 (-0.0929, 0.0631)	0.7059	0.9986	-0.0133 (-0.0942, 0.0676)	0.7459	0.9993
MEHP	Creatine	0.0032 (-0.0589, 0.0653)	0.9187	0.9986	0.0084 (-0.0542, 0.0711)	0.7904	0.9993
MEHP	Cystine	0.1090 (-0.0463, 0.2642)	0.1673	0.9986	0.1173 (-0.0441, 0.2787)	0.1528	0.9993
MEHP	Ethanolamine	-0.0075 (-0.0888, 0.0737)	0.8551	0.9986	-0.0075 (-0.0912, 0.0763)	0.8603	0.9993
MEHP	Formate	-0.0460 (-0.1361, 0.0442)	0.3151	0.9986	-0.0405 (-0.1318, 0.0507)	0.3808	0.9993
MEHP	Fumarate	-0.0191 (-0.1340, 0.0957)	0.7422	0.9986	-0.0140 (-0.1333, 0.1053)	0.8166	0.9993
MEHP	Glucitol	-0.0734 (-0.1638, 0.0170)	0.1108	0.9986	-0.0678 (-0.1613, 0.0257)	0.1538	0.9993
MEHP	Glutamate	-0.0198 (-0.0879, 0.0483)	0.5654	0.9986	-0.0146 (-0.0846, 0.0554)	0.6804	0.9993
MEHP	Glutamine	-0.0287 (-0.0754, 0.0179)	0.2253	0.9986	-0.0295 (-0.0759, 0.0169)	0.2111	0.9993
MEHP	Glutathione	-0.1680 (-0.3407, 0.0048)	0.0566	0.9986	-0.1867 (-0.3624, -0.0109)	0.0376	0.9993
MEHP	Glycerol	0.0165 (-0.0738, 0.1067)	0.7188	0.9986	0.0194 (-0.0741, 0.1129)	0.6823	0.9993
MEHP	Glycine	-0.0031 (-0.0771, 0.0709)	0.9343	0.9986	0.0110 (-0.0650, 0.0869)	0.7750	0.9993
MEHP	Hypoxanthine	-0.0287 (-0.0981, 0.0408)	0.4155	0.9986	-0.0215 (-0.0924, 0.0495)	0.5500	0.9993
MEHP	Inosine	-0.0808 (-0.2429, 0.0812)	0.3254	0.9986	-0.0981 (-0.2619, 0.0657)	0.2378	0.9993
MEHP	Isoleucine	-0.0034 (-0.0849, 0.0781)	0.9341	0.9986	0.0130 (-0.0700, 0.0960)	0.7567	0.9993
MEHP	Kynurenine	-0.1063 (-0.2304, 0.0178)	0.0926	0.9986	-0.0972 (-0.2254, 0.0310)	0.1361	0.9993
MEHP	Lactate	0.0031 (-0.0497, 0.0560)	0.9077	0.9986	0.0037 (-0.0509, 0.0582)	0.8945	0.9993
MEHP	Leucine	-0.0066 (-0.0911, 0.0778)	0.8765	0.9986	0.0118 (-0.0740, 0.0975)	0.7864	0.9993
MEHP	Lysine	0.0083 (-0.0874, 0.1041)	0.8635	0.9986	0.0317 (-0.0656, 0.1290)	0.5202	0.9993

		Total Effect			Direct Effect		
**Phthalate**	**Metabolite**	**Estimate (95% CI)**	**p**	**FDR p**	**Estimate (95% CI)**	**p**	**FDR p**
MEHP	Methionine	0.0251 (-0.0644, 0.1145)	0.5803	0.9986	0.0422 (-0.0480, 0.1324)	0.3559	0.9993
MEHP	myo-Inositol	0.0490 (-0.0313, 0.1293)	0.2296	0.9986	0.0476 (-0.0345, 0.1298)	0.2530	0.9993
MEHP	N-Acetylneuraminate	-0.0561 (-0.1537, 0.0416)	0.2581	0.9986	-0.0482 (-0.1495, 0.0531)	0.3483	0.9993
MEHP	NAD+	-0.1255 (-0.3096, 0.0587)	0.1799	0.9986	-0.1411 (-0.3305, 0.0484)	0.1430	0.9993
MEHP	Niacinamide	0.0464 (-0.3008, 0.3936)	0.7918	0.9986	0.0015 (-0.3551, 0.3581)	0.9934	0.9998
MEHP	O-Acetylcarnitine	-0.1047 (-0.1884, -0.0210)	0.0147	0.9986	-0.1038 (-0.1908, -0.0169)	0.0197	0.9993
MEHP	O-Phosphocholine	-0.2174 (-0.4366, 0.0018)	0.0519	0.9986	-0.2143 (-0.4424, 0.0139)	0.0654	0.9993
MEHP	O-Phosphoethanolamine	-0.1752 (-0.3318, -0.0186)	0.0287	0.9986	-0.1571 (-0.3177, 0.0034)	0.0549	0.9993
MEHP	Ornithine	0.0091 (-0.1149, 0.1332)	0.8842	0.9986	0.0270 (-0.1005, 0.1544)	0.6762	0.9993
MEHP	Pantothenate	-0.0056 (-0.1161, 0.1050)	0.9207	0.9986	-0.0130 (-0.1260, 0.1000)	0.8202	0.9993
MEHP	Phenylalanine	-0.0070 (-0.0926, 0.0787)	0.8722	0.9986	0.0086 (-0.0786, 0.0958)	0.8463	0.9993
MEHP	Proline	0.0268 (-0.0464, 0.0999)	0.4708	0.9986	0.0388 (-0.0361, 0.1136)	0.3074	0.9993
MEHP	Pyroglutamate	0.0080 (-0.0933, 0.1094)	0.8758	0.9986	0.0058 (-0.0997, 0.1112)	0.9141	0.9993
MEHP	Serine	0.0118 (-0.0754, 0.0990)	0.7896	0.9986	0.0300 (-0.0583, 0.1183)	0.5027	0.9993
MEHP	sn-Glycero-3-phosphocholine	-0.0865 (-0.2218, 0.0488)	0.2081	0.9986	-0.0691 (-0.2037, 0.0655)	0.3116	0.9993
MEHP	Succinate	-0.0387 (-0.1924, 0.1149)	0.6186	0.9986	-0.0415 (-0.1997, 0.1167)	0.6044	0.9993
MEHP	Taurine	-0.0415 (-0.1068, 0.0238)	0.2109	0.9986	-0.0380 (-0.1054, 0.0294)	0.2664	0.9993
MEHP	Threonine	0.0218 (-0.0573, 0.1010)	0.5858	0.9986	0.0402 (-0.0393, 0.1197)	0.3183	0.9993
MEHP	Tryptophan	0.0368 (-0.1010, 0.1746)	0.5983	0.9986	0.0596 (-0.0810, 0.2002)	0.4028	0.9993
MEHP	Tyrosine	0.0025 (-0.0778, 0.0828)	0.9514	0.9986	0.0198 (-0.0618, 0.1014)	0.6313	0.9993
MEHP	Uracil	-0.0129 (-0.1271, 0.1013)	0.8232	0.9986	-0.0127 (-0.1305, 0.1051)	0.8319	0.9993
MEHP	Uridine	-0.0325 (-0.1801, 0.1152)	0.6642	0.9986	-0.0217 (-0.1739, 0.1305)	0.7781	0.9993
MEHP	Valine	0.0060 (-0.0694, 0.0814)	0.8752	0.9986	0.0226 (-0.0539, 0.0991)	0.5593	0.9993
MEOHP	1,3-Dihydroxyacetone	0.0261 (-0.1603, 0.2124)	0.7823	0.9986	0.0063 (-0.1920, 0.2046)	0.9500	0.9993
MEOHP	2-Hydroxybutyrate	-0.0231 (-0.1148, 0.0686)	0.6191	0.9986	-0.0437 (-0.1404, 0.0530)	0.3726	0.9993
MEOHP	3-Hydroxybutyrate	-0.0695 (-0.2759, 0.1369)	0.5066	0.9986	-0.0640 (-0.2788, 0.1508)	0.5561	0.9993
MEOHP	4-Aminobutyrate	0.0011 (-0.1147, 0.1168)	0.9854	0.9986	0.0253 (-0.0920, 0.1425)	0.6703	0.9993
MEOHP	4-Hydroxybutyrate	0.0153 (-0.2482, 0.2788)	0.9087	0.9986	-0.0165 (-0.2971, 0.2640)	0.9073	0.9993
MEOHP	Acetate	-0.0511 (-0.2118, 0.1095)	0.5301	0.9986	-0.0506 (-0.2204, 0.1192)	0.5562	0.9993
MEOHP	Alanine	0.0044 (-0.0681, 0.0770)	0.9036	0.9986	0.0205 (-0.0544, 0.0954)	0.5886	0.9993
MEOHP	Arginine	-0.0058 (-0.1026, 0.0910)	0.9055	0.9986	0.0048 (-0.0976, 0.1073)	0.9255	0.9993
MEOHP	Asparagine	-0.0001 (-0.0847, 0.0846)	0.9986	0.9986	0.0232 (-0.0643, 0.1106)	0.6008	0.9993
MEOHP	Aspartate	-0.0033 (-0.0775, 0.0709)	0.9302	0.9986	-0.0008 (-0.0789, 0.0773)	0.9837	0.9996
MEOHP	Betaine	-0.0855 (-0.1684, -0.0027)	0.0431	0.9986	-0.0869 (-0.1742, 0.0003)	0.0508	0.9993
MEOHP	Carnitine	-0.0623 (-0.1349, 0.0103)	0.0919	0.9986	-0.0595 (-0.1366, 0.0176)	0.1292	0.9993
MEOHP	Choline	-0.0012 (-0.0798, 0.0775)	0.9769	0.9986	0.0010 (-0.0824, 0.0844)	0.9808	0.9993
MEOHP	Creatine	0.0065 (-0.0561, 0.0691)	0.8377	0.9986	0.0053 (-0.0592, 0.0699)	0.8705	0.9993
MEOHP	Cystine	0.1203 (-0.0359, 0.2766)	0.1301	0.9986	0.1331 (-0.0329, 0.2991)	0.1150	0.9993
MEOHP	Ethanolamine	-0.0092 (-0.0912, 0.0727)	0.8239	0.9986	-0.0021 (-0.0883, 0.0842)	0.9621	0.9993
MEOHP	Formate	-0.0172 (-0.1084, 0.0740)	0.7100	0.9986	-0.0132 (-0.1075, 0.0811)	0.7821	0.9993
MEOHP	Fumarate	-0.0068 (-0.1226, 0.1090)	0.9081	0.9986	-0.0031 (-0.1260, 0.1198)	0.9607	0.9993
MEOHP	Glucitol	-0.0637 (-0.1551, 0.0276)	0.1699	0.9986	-0.0629 (-0.1593, 0.0336)	0.1993	0.9993
MEOHP	Glutamate	-0.0006 (-0.0693, 0.0682)	0.9871	0.9986	0.0040 (-0.0681, 0.0761)	0.9124	0.9993
MEOHP	Glutamine	-0.0153 (-0.0626, 0.0319)	0.5217	0.9986	-0.0136 (-0.0617, 0.0345)	0.5761	0.9993
MEOHP	Glutathione	-0.1452 (-0.3201, 0.0296)	0.1026	0.9986	-0.1685 (-0.3503, 0.0133)	0.0690	0.9993
MEOHP	Glycerol	0.0265 (-0.0643, 0.1174)	0.5643	0.9986	0.0329 (-0.0633, 0.1291)	0.4992	0.9993
MEOHP	Glycine	0.0173 (-0.0572, 0.0919)	0.6466	0.9986	0.0332 (-0.0448, 0.1112)	0.4009	0.9993
MEOHP	Hypoxanthine	-0.0055 (-0.0757, 0.0646)	0.8763	0.9986	0.0059 (-0.0672, 0.0791)	0.8725	0.9993
MEOHP	Inosine	-0.0926 (-0.2558, 0.0706)	0.2635	0.9986	-0.1236 (-0.2918, 0.0446)	0.1483	0.9993
MEOHP	Isoleucine	0.0102 (-0.0720, 0.0924)	0.8065	0.9986	0.0316 (-0.0537, 0.1169)	0.4650	0.9993
MEOHP	Kynurenine	-0.1070 (-0.2322, 0.0181)	0.0931	0.9986	-0.0945 (-0.2267, 0.0377)	0.1597	0.9993
MEOHP	Lactate	0.0137 (-0.0395, 0.0670)	0.6103	0.9986	0.0166 (-0.0395, 0.0727)	0.5591	0.9993
MEOHP	Leucine	0.0070 (-0.0782, 0.0921)	0.8715	0.9986	0.0306 (-0.0576, 0.1188)	0.4928	0.9993
MEOHP	Lysine	0.0031 (-0.0935, 0.0996)	0.9502	0.9986	0.0327 (-0.0675, 0.1329)	0.5194	0.9993
MEOHP	Methionine	0.0390 (-0.0510, 0.1291)	0.3926	0.9986	0.0633 (-0.0292, 0.1559)	0.1780	0.9993
MEOHP	myo-Inositol	0.0362 (-0.0450, 0.1173)	0.3795	0.9986	0.0258 (-0.0591, 0.1108)	0.5479	0.9993
MEOHP	N-Acetylneuraminate	-0.0528 (-0.1514, 0.0457)	0.2906	0.9986	-0.0468 (-0.1512, 0.0576)	0.3768	0.9993
MEOHP	NAD+	-0.1426 (-0.3279, 0.0427)	0.1303	0.9986	-0.1553 (-0.3502, 0.0396)	0.1173	0.9993
MEOHP	Niacinamide	0.1012 (-0.2485, 0.4509)	0.5679	0.9986	0.0749 (-0.2922, 0.4419)	0.6870	0.9993
MEOHP	O-Acetylcarnitine	-0.0933 (-0.1781, -0.0084)	0.0315	0.9986	-0.0877 (-0.1780, 0.0025)	0.0567	0.9993

		Total Effect			Direct Effect		
**Phthalate**	**Metabolite**	**Estimate (95% CI)**	**p**	**FDR p**	**Estimate (95% CI)**	**p**	**FDR p**
MEOHP	O-Phosphocholine	-0.2913 (-0.5097, -0.0729)	0.0093	0.9986	-0.2890 (-0.5216, -0.0564)	0.0153	0.9993
MEOHP	O-Phosphoethanolamine	-0.1680 (-0.3261, -0.0098)	0.0376	0.9986	-0.1595 (-0.3249, 0.0059)	0.0587	0.9993
MEOHP	Ornithine	0.0102 (-0.1148, 0.1353)	0.8719	0.9986	0.0252 (-0.1061, 0.1565)	0.7046	0.9993
MEOHP	Pantothenate	0.0210 (-0.0903, 0.1324)	0.7092	0.9986	0.0249 (-0.0914, 0.1413)	0.6722	0.9993
MEOHP	Phenylalanine	0.0182 (-0.0681, 0.1044)	0.6777	0.9986	0.0374 (-0.0522, 0.1269)	0.4104	0.9993
MEOHP	Proline	0.0384 (-0.0352, 0.1120)	0.3040	0.9986	0.0545 (-0.0223, 0.1313)	0.1624	0.9993
MEOHP	Pyroglutamate	0.0544 (-0.0474, 0.1561)	0.2923	0.9986	0.0533 (-0.0549, 0.1615)	0.3311	0.9993
MEOHP	Serine	0.0240 (-0.0638, 0.1119)	0.5896	0.9986	0.0484 (-0.0424, 0.1391)	0.2934	0.9993
MEOHP	sn-Glycero-3-phosphocholine	-0.0943 (-0.2306, 0.0419)	0.1730	0.9986	-0.0615 (-0.2003, 0.0773)	0.3823	0.9993
MEOHP	Succinate	-0.0509 (-0.2057, 0.1039)	0.5165	0.9986	-0.0498 (-0.2127, 0.1131)	0.5461	0.9993
MEOHP	Taurine	-0.0336 (-0.0996, 0.0324)	0.3156	0.9986	-0.0293 (-0.0988, 0.0403)	0.4067	0.9993
MEOHP	Threonine	0.0216 (-0.0581, 0.1014)	0.5924	0.9986	0.0457 (-0.0361, 0.1275)	0.2704	0.9993
MEOHP	Tryptophan	0.0894 (-0.0488, 0.2276)	0.2028	0.9986	0.1081 (-0.0358, 0.2520)	0.1394	0.9993
MEOHP	Tyrosine	0.0117 (-0.0692, 0.0926)	0.7750	0.9986	0.0347 (-0.0491, 0.1186)	0.4140	0.9993
MEOHP	Uracil	0.0252 (-0.0898, 0.1403)	0.6653	0.9986	0.0348 (-0.0864, 0.1560)	0.5713	0.9993
MEOHP	Uridine	-0.0133 (-0.1623, 0.1356)	0.8597	0.9986	-0.0083 (-0.1651, 0.1486)	0.9169	0.9993
MEOHP	Valine	0.0175 (-0.0585, 0.0934)	0.6500	0.9986	0.0379 (-0.0408, 0.1165)	0.3424	0.9993
MEP	1,3-Dihydroxyacetone	0.0255 (-0.1139, 0.1649)	0.7178	0.9986	0.0190 (-0.1286, 0.1667)	0.7989	0.9993
MEP	2-Hydroxybutyrate	-0.0372 (-0.1056, 0.0311)	0.2833	0.9986	-0.0389 (-0.1108, 0.0330)	0.2861	0.9993
MEP	3-Hydroxybutyrate	-0.0540 (-0.2084, 0.1005)	0.4905	0.9986	-0.0498 (-0.2097, 0.1102)	0.5392	0.9993
MEP	4-Aminobutyrate	0.0693 (-0.0165, 0.1551)	0.1123	0.9986	0.0774 (-0.0089, 0.1636)	0.0784	0.9993
MEP	4-Hydroxybutyrate	0.0378 (-0.1592, 0.2349)	0.7047	0.9986	0.0498 (-0.1590, 0.2586)	0.6375	0.9993
MEP	Acetate	0.0495 (-0.0706, 0.1696)	0.4166	0.9986	0.0508 (-0.0755, 0.1771)	0.4275	0.9993
MEP	Alanine	-0.0063 (-0.0606, 0.0480)	0.8183	0.9986	-0.0101 (-0.0659, 0.0457)	0.7211	0.9993
MEP	Arginine	-0.0226 (-0.0949, 0.0497)	0.5375	0.9986	-0.0260 (-0.1021, 0.0501)	0.5004	0.9993
MEP	Asparagine	-0.0088 (-0.0722, 0.0545)	0.7829	0.9986	-0.0082 (-0.0733, 0.0570)	0.8047	0.9993
MEP	Aspartate	-0.0211 (-0.0765, 0.0343)	0.4519	0.9986	-0.0181 (-0.0761, 0.0400)	0.5392	0.9993
MEP	Betaine	-0.0069 (-0.0699, 0.0561)	0.8277	0.9986	0.0054 (-0.0606, 0.0714)	0.8709	0.9993
MEP	Carnitine	-0.0201 (-0.0749, 0.0347)	0.4695	0.9986	-0.0162 (-0.0741, 0.0417)	0.5813	0.9993
MEP	Choline	0.0206 (-0.0382, 0.0793)	0.4892	0.9986	0.0151 (-0.0469, 0.0771)	0.6303	0.9993
MEP	Creatine	-0.0248 (-0.0715, 0.0219)	0.2950	0.9986	-0.0148 (-0.0628, 0.0332)	0.5423	0.9993
MEP	Cystine	0.0480 (-0.0697, 0.1657)	0.4208	0.9986	0.0472 (-0.0774, 0.1718)	0.4550	0.9993
MEP	Ethanolamine	0.0251 (-0.0360, 0.0863)	0.4173	0.9986	0.0221 (-0.0420, 0.0862)	0.4967	0.9993
MEP	Formate	-0.0154 (-0.0836, 0.0529)	0.6569	0.9986	-0.0226 (-0.0927, 0.0475)	0.5249	0.9993
MEP	Fumarate	-0.0021 (-0.0888, 0.0846)	0.9620	0.9986	0.0093 (-0.0823, 0.1008)	0.8415	0.9993
MEP	Glucitol	-0.0542 (-0.1224, 0.0141)	0.1188	0.9986	-0.0592 (-0.1307, 0.0124)	0.1041	0.9993
MEP	Glutamate	-0.0035 (-0.0550, 0.0479)	0.8917	0.9986	-0.0011 (-0.0548, 0.0527)	0.9690	0.9993
MEP	Glutamine	-0.0068 (-0.0421, 0.0286)	0.7061	0.9986	0.0007 (-0.0351, 0.0366)	0.9671	0.9993
MEP	Glutathione	-0.0898 (-0.2211, 0.0414)	0.1781	0.9986	-0.0918 (-0.2281, 0.0444)	0.1846	0.9993
MEP	Glycerol	0.0303 (-0.0375, 0.0982)	0.3781	0.9986	0.0214 (-0.0502, 0.0931)	0.5549	0.9993
MEP	Glycine	0.0168 (-0.0390, 0.0725)	0.5531	0.9986	0.0204 (-0.0378, 0.0786)	0.4890	0.9993
MEP	Hypoxanthine	0.0078 (-0.0446, 0.0603)	0.7677	0.9986	0.0074 (-0.0470, 0.0619)	0.7875	0.9993
MEP	Inosine	-0.1354 (-0.2558, -0.0150)	0.0278	0.9986	-0.1208 (-0.2453, 0.0037)	0.0570	0.9993
MEP	Isoleucine	0.0139 (-0.0476, 0.0753)	0.6554	0.9986	0.0106 (-0.0531, 0.0742)	0.7433	0.9993
MEP	Kynurenine	0.0406 (-0.0538, 0.1351)	0.3963	0.9986	0.0480 (-0.0509, 0.1469)	0.3388	0.9993
MEP	Lactate	0.0004 (-0.0395, 0.0403)	0.9839	0.9986	0.0010 (-0.0409, 0.0429)	0.9625	0.9993
MEP	Leucine	0.0137 (-0.0500, 0.0774)	0.6704	0.9986	0.0109 (-0.0549, 0.0767)	0.7434	0.9993
MEP	Lysine	0.0026 (-0.0697, 0.0748)	0.9438	0.9986	-0.0021 (-0.0769, 0.0726)	0.9547	0.9993
MEP	Methionine	0.0386 (-0.0286, 0.1058)	0.2582	0.9986	0.0303 (-0.0389, 0.0995)	0.3878	0.9993
MEP	myo-Inositol	0.0019 (-0.0590, 0.0628)	0.9507	0.9986	0.0111 (-0.0522, 0.0744)	0.7286	0.9993
MEP	N-Acetylneuraminate	-0.0166 (-0.0906, 0.0573)	0.6569	0.9986	-0.0229 (-0.1008, 0.0550)	0.5612	0.9993
MEP	NAD+	-0.1048 (-0.2435, 0.0339)	0.1374	0.9986	-0.1031 (-0.2486, 0.0423)	0.1630	0.9993
MEP	Niacinamide	-0.0131 (-0.2751, 0.2489)	0.9213	0.9986	-0.0305 (-0.3040, 0.2430)	0.8254	0.9993
MEP	O-Acetylcarnitine	-0.0218 (-0.0863, 0.0428)	0.5058	0.9986	-0.0237 (-0.0918, 0.0445)	0.4931	0.9993
MEP	O-Phosphocholine	-0.1065 (-0.2733, 0.0604)	0.2089	0.9986	-0.0994 (-0.2760, 0.0772)	0.2673	0.9993
MEP	O-Phosphoethanolamine	-0.0999 (-0.2190, 0.0193)	0.0996	0.9986	-0.0862 (-0.2103, 0.0379)	0.1715	0.9993
MEP	Ornithine	0.0260 (-0.0675, 0.1195)	0.5828	0.9986	0.0257 (-0.0721, 0.1235)	0.6037	0.9993
MEP	Pantothenate	0.0222 (-0.0611, 0.1055)	0.5982	0.9986	0.0254 (-0.0612, 0.1120)	0.5623	0.9993
MEP	Phenylalanine	0.0091 (-0.0555, 0.0737)	0.7812	0.9986	0.0045 (-0.0624, 0.0714)	0.8949	0.9993
MEP	Proline	0.0179 (-0.0373, 0.0732)	0.5222	0.9986	0.0167 (-0.0409, 0.0743)	0.5678	0.9993

		Total Effect			Direct Effect		
**Phthalate**	**Metabolite**	**Estimate (95% CI)**	**p**	**FDR p**	**Estimate (95% CI)**	**p**	**FDR p**
MEP	Pyroglutamate	-0.0015 (-0.0780, 0.0749)	0.9682	0.9986	-0.0051 (-0.0860, 0.0758)	0.9013	0.9993
MEP	Serine	0.0219 (-0.0438, 0.0876)	0.5109	0.9986	0.0194 (-0.0484, 0.0872)	0.5720	0.9993
MEP	sn-Glycero-3-phosphocholine	-0.0142 (-0.1168, 0.0885)	0.7855	0.9986	-0.0319 (-0.1355, 0.0717)	0.5430	0.9993
MEP	Succinate	-0.0334 (-0.1492, 0.0825)	0.5697	0.9986	-0.0395 (-0.1608, 0.0818)	0.5200	0.9993
MEP	Taurine	-0.0268 (-0.0762, 0.0225)	0.2839	0.9986	-0.0230 (-0.0748, 0.0288)	0.3812	0.9993
MEP	Threonine	0.0028 (-0.0570, 0.0625)	0.9273	0.9986	-0.0015 (-0.0627, 0.0597)	0.9617	0.9993
MEP	Tryptophan	0.0118 (-0.0922, 0.1159)	0.8225	0.9986	0.0198 (-0.0883, 0.1279)	0.7170	0.9993
MEP	Tyrosine	0.0277 (-0.0327, 0.0881)	0.3655	0.9986	0.0271 (-0.0354, 0.0895)	0.3925	0.9993
MEP	Uracil	0.0454 (-0.0404, 0.1312)	0.2970	0.9986	0.0283 (-0.0620, 0.1185)	0.5363	0.9993
MEP	Uridine	-0.0079 (-0.1193, 0.1036)	0.8891	0.9986	0.0023 (-0.1145, 0.1191)	0.9685	0.9993
MEP	Valine	0.0106 (-0.0462, 0.0675)	0.7117	0.9986	0.0088 (-0.0500, 0.0675)	0.7677	0.9993
MHBP	1,3-Dihydroxyacetone	-0.1401 (-0.5541, 0.2739)	0.5043	0.9986	-0.1333 (-0.5609, 0.2944)	0.5384	0.9993
MHBP	2-Hydroxybutyrate	-0.0993 (-0.3028, 0.1042)	0.3359	0.9986	-0.0938 (-0.3027, 0.1150)	0.3755	0.9993
MHBP	3-Hydroxybutyrate	0.2759 (-0.1816, 0.7334)	0.2350	0.9986	0.2178 (-0.2452, 0.6808)	0.3535	0.9993
MHBP	4-Aminobutyrate	0.0272 (-0.2303, 0.2847)	0.8348	0.9986	0.0853 (-0.1677, 0.3383)	0.5059	0.9993
MHBP	4-Hydroxybutyrate	0.2253 (-0.3595, 0.8102)	0.4472	0.9986	0.2192 (-0.3855, 0.8240)	0.4743	0.9993
MHBP	Acetate	-0.1527 (-0.5097, 0.2044)	0.3991	0.9986	-0.1359 (-0.5024, 0.2306)	0.4644	0.9993
MHBP	Alanine	-0.0402 (-0.2015, 0.1211)	0.6225	0.9986	-0.0206 (-0.1825, 0.1413)	0.8013	0.9993
MHBP	Arginine	-0.0291 (-0.2445, 0.1862)	0.7893	0.9986	-0.0322 (-0.2534, 0.1889)	0.7733	0.9993
MHBP	Asparagine	-0.0002 (-0.1885, 0.1882)	0.9985	0.9986	-0.0009 (-0.1900, 0.1882)	0.9927	0.9998
MHBP	Aspartate	-0.0212 (-0.1863, 0.1438)	0.7994	0.9986	-0.0366 (-0.2052, 0.1320)	0.6680	0.9993
MHBP	Betaine	-0.0123 (-0.1996, 0.1750)	0.8967	0.9986	-0.0050 (-0.1964, 0.1865)	0.9589	0.9993
MHBP	Carnitine	0.0107 (-0.1526, 0.1741)	0.8966	0.9986	0.0243 (-0.1438, 0.1924)	0.7751	0.9993
MHBP	Choline	0.0063 (-0.1687, 0.1814)	0.9430	0.9986	0.0119 (-0.1681, 0.1920)	0.8957	0.9993
MHBP	Creatine	-0.1062 (-0.2443, 0.0319)	0.1306	0.9986	-0.0966 (-0.2349, 0.0418)	0.1694	0.9993
MHBP	Cystine	0.1003 (-0.2502, 0.4508)	0.5722	0.9986	0.1035 (-0.2582, 0.4653)	0.5721	0.9993
MHBP	Ethanolamine	-0.0187 (-0.2010, 0.1636)	0.8394	0.9986	-0.0036 (-0.1899, 0.1827)	0.9695	0.9993
MHBP	Formate	-0.1370 (-0.3386, 0.0646)	0.1811	0.9986	-0.1233 (-0.3258, 0.0791)	0.2300	0.9993
MHBP	Fumarate	0.0099 (-0.2478, 0.2675)	0.9398	0.9986	-0.0048 (-0.2702, 0.2607)	0.9718	0.9993
MHBP	Glucitol	-0.0831 (-0.2875, 0.1212)	0.4223	0.9986	-0.0816 (-0.2909, 0.1276)	0.4414	0.9993
MHBP	Glutamate	0.0292 (-0.1237, 0.1820)	0.7064	0.9986	0.0325 (-0.1232, 0.1882)	0.6803	0.9993
MHBP	Glutamine	-0.0020 (-0.1073, 0.1032)	0.9698	0.9986	0.0060 (-0.0980, 0.1099)	0.9093	0.9993
MHBP	Glutathione	-0.0073 (-0.4004, 0.3858)	0.9707	0.9986	0.0117 (-0.3865, 0.4099)	0.9537	0.9993
MHBP	Glycerol	0.0303 (-0.1721, 0.2327)	0.7676	0.9986	0.0357 (-0.1724, 0.2437)	0.7350	0.9993
MHBP	Glycine	0.0286 (-0.1373, 0.1946)	0.7334	0.9986	0.0325 (-0.1364, 0.2015)	0.7037	0.9993
MHBP	Hypoxanthine	-0.0305 (-0.1865, 0.1255)	0.6996	0.9986	-0.0170 (-0.1750, 0.1411)	0.8320	0.9993
MHBP	Inosine	-0.0453 (-0.4102, 0.3196)	0.8063	0.9986	-0.0610 (-0.4274, 0.3053)	0.7421	0.9993
MHBP	Isoleucine	-0.0027 (-0.1856, 0.1802)	0.9765	0.9986	0.0024 (-0.1824, 0.1871)	0.9797	0.9993
MHBP	Kynurenine	-0.0492 (-0.3307, 0.2323)	0.7302	0.9986	-0.0520 (-0.3397, 0.2358)	0.7212	0.9993
MHBP	Lactate	0.0450 (-0.0732, 0.1633)	0.4525	0.9986	0.0613 (-0.0596, 0.1822)	0.3178	0.9993
MHBP	Leucine	0.0019 (-0.1876, 0.1914)	0.9843	0.9986	0.0079 (-0.1830, 0.1988)	0.9348	0.9993
MHBP	Lysine	-0.0301 (-0.2448, 0.1847)	0.7822	0.9986	-0.0315 (-0.2482, 0.1853)	0.7743	0.9993
MHBP	Methionine	0.0203 (-0.1806, 0.2212)	0.8421	0.9986	0.0220 (-0.1793, 0.2234)	0.8289	0.9993
MHBP	myo-Inositol	0.0229 (-0.1582, 0.2040)	0.8028	0.9986	0.0040 (-0.1797, 0.1877)	0.9659	0.9993
MHBP	N-Acetylneuraminate	-0.0053 (-0.2255, 0.2149)	0.9622	0.9986	-0.0120 (-0.2383, 0.2142)	0.9163	0.9993
MHBP	NAD+	-0.1335 (-0.5489, 0.2819)	0.5260	0.9986	-0.1106 (-0.5355, 0.3142)	0.6071	0.9993
MHBP	Niacinamide	0.3201 (-0.4569, 1.0971)	0.4165	0.9986	0.3069 (-0.4846, 1.0983)	0.4442	0.9993
MHBP	O-Acetylcarnitine	0.0599 (-0.1321, 0.2519)	0.5380	0.9986	0.0634 (-0.1342, 0.2610)	0.5262	0.9993
MHBP	O-Phosphocholine	-0.0859 (-0.5849, 0.4131)	0.7339	0.9986	-0.0520 (-0.5667, 0.4628)	0.8420	0.9993
MHBP	O-Phosphoethanolamine	0.0808 (-0.2770, 0.4385)	0.6558	0.9986	0.0519 (-0.3107, 0.4144)	0.7775	0.9993
MHBP	Ornithine	-0.0495 (-0.3277, 0.2286)	0.7251	0.9986	-0.0533 (-0.3370, 0.2304)	0.7105	0.9993
MHBP	Pantothenate	-0.1233 (-0.3703, 0.1237)	0.3252	0.9986	-0.1065 (-0.3573, 0.1443)	0.4021	0.9993
MHBP	Phenylalanine	0.0163 (-0.1758, 0.2084)	0.8667	0.9986	0.0202 (-0.1738, 0.2142)	0.8371	0.9993
MHBP	Proline	-0.0057 (-0.1702, 0.1588)	0.9453	0.9986	0.0035 (-0.1638, 0.1708)	0.9671	0.9993
MHBP	Pyroglutamate	-0.1634 (-0.3889, 0.0622)	0.1543	0.9986	-0.1601 (-0.3929, 0.0728)	0.1761	0.9993
MHBP	Serine	0.0075 (-0.1881, 0.2032)	0.9393	0.9986	0.0069 (-0.1900, 0.2038)	0.9448	0.9993
MHBP	sn-Glycero-3-phosphocholine	-0.1239 (-0.4284, 0.1807)	0.4225	0.9986	-0.1098 (-0.4100, 0.1904)	0.4705	0.9993
MHBP	Succinate	0.0343 (-0.3106, 0.3792)	0.8443	0.9986	0.0683 (-0.2840, 0.4205)	0.7019	0.9993
MHBP	Taurine	0.0230 (-0.1243, 0.1703)	0.7580	0.9986	0.0354 (-0.1152, 0.1859)	0.6428	0.9993

		Total Effect			Direct Effect		
Phthalate	Metabolite	Estimate (95% CI)	p	FDR p	Estimate (95% CI)	p	FDR p
MHBP	Threonine	0.0440 (-0.1335, 0.2216)	0.6243	0.9986	0.0422 (-0.1352, 0.2197)	0.6382	0.9993
MHBP	Tryptophan	-0.0031 (-0.3126, 0.3064)	0.9840	0.9986	0.0007 (-0.3130, 0.3143)	0.9967	0.9998
MHBP	Tyrosine	0.0183 (-0.1618, 0.1984)	0.8410	0.9986	0.0195 (-0.1621, 0.2011)	0.8321	0.9993
MHBP	Uracil	0.0374 (-0.2187, 0.2935)	0.7730	0.9986	0.0411 (-0.2209, 0.3032)	0.7565	0.9993
MHBP	Uridine	0.0169 (-0.3145, 0.3483)	0.9198	0.9986	-0.0041 (-0.3429, 0.3347)	0.9808	0.9993
MHBP	Valine	-0.0063 (-0.1755, 0.1628)	0.9409	0.9986	-0.0018 (-0.1723, 0.1686)	0.9831	0.9996
MHiBP	1,3-Dihydroxyacetone	-0.1610 (-0.4575, 0.1354)	0.2844	0.9986	-0.1955 (-0.5048, 0.1138)	0.2132	0.9993
MHiBP	2-Hydroxybutyrate	-0.0439 (-0.1903, 0.1026)	0.5543	0.9986	-0.0657 (-0.2176, 0.0861)	0.3931	0.9993
MHiBP	3-Hydroxybutyrate	0.0612 (-0.2690, 0.3914)	0.7145	0.9986	0.0983 (-0.2390, 0.4355)	0.5652	0.9993
MHiBP	4-Aminobutyrate	0.0728 (-0.1117, 0.2573)	0.4362	0.9986	0.0738 (-0.1100, 0.2575)	0.4284	0.9993
MHiBP	4-Hydroxybutyrate	0.2779 (-0.1402, 0.6960)	0.1908	0.9986	0.2872 (-0.1502, 0.7246)	0.1961	0.9993
MHiBP	Acetate	-0.0773 (-0.3340, 0.1794)	0.5523	0.9986	-0.0615 (-0.3283, 0.2052)	0.6487	0.9993
MHiBP	Alanine	0.0319 (-0.0839, 0.1477)	0.5864	0.9986	0.0494 (-0.0680, 0.1667)	0.4066	0.9993
MHiBP	Arginine	0.0231 (-0.1315, 0.1777)	0.7679	0.9986	0.0272 (-0.1335, 0.1880)	0.7377	0.9993
MHiBP	Asparagine	0.0160 (-0.1192, 0.1512)	0.8149	0.9986	0.0394 (-0.0979, 0.1766)	0.5712	0.9993
MHiBP	Aspartate	0.0127 (-0.1058, 0.1313)	0.8320	0.9986	0.0249 (-0.0976, 0.1475)	0.6880	0.9993
MHiBP	Betaine	0.0420 (-0.0923, 0.1763)	0.5373	0.9986	0.0690 (-0.0696, 0.2076)	0.3263	0.9993
MHiBP	Carnitine	0.0003 (-0.1170, 0.1176)	0.9960	0.9986	0.0016 (-0.1207, 0.1238)	0.9799	0.9993
MHiBP	Choline	0.0219 (-0.1037, 0.1475)	0.7309	0.9986	0.0296 (-0.1011, 0.1604)	0.6545	0.9993
MHiBP	Creatine	0.0010 (-0.0991, 0.1011)	0.9840	0.9986	0.0139 (-0.0874, 0.1152)	0.7863	0.9993
MHiBP	Cystine	0.1164 (-0.1347, 0.3675)	0.3608	0.9986	0.1084 (-0.1541, 0.3710)	0.4152	0.9993
MHiBP	Ethanolamine	-0.0219 (-0.1527, 0.1090)	0.7413	0.9986	-0.0164 (-0.1518, 0.1189)	0.8104	0.9993
MHiBP	Formate	-0.0752 (-0.2204, 0.0700)	0.3076	0.9986	-0.0521 (-0.1999, 0.0956)	0.4860	0.9993
MHiBP	Fumarate	-0.1044 (-0.2886, 0.0797)	0.2638	0.9986	-0.1036 (-0.2957, 0.0885)	0.2876	0.9993
MHiBP	Glucitol	-0.0993 (-0.2454, 0.0468)	0.1809	0.9986	-0.0810 (-0.2327, 0.0708)	0.2931	0.9993
MHiBP	Glutamate	0.0218 (-0.0880, 0.1315)	0.6955	0.9986	0.0399 (-0.0732, 0.1529)	0.4863	0.9993
MHiBP	Glutamine	0.0314 (-0.0440, 0.1067)	0.4117	0.9986	0.0388 (-0.0364, 0.1141)	0.3089	0.9993
MHiBP	Glutathione	-0.0388 (-0.3210, 0.2435)	0.7863	0.9986	-0.0304 (-0.3198, 0.2590)	0.8357	0.9993
MHiBP	Glycerol	0.0472 (-0.0979, 0.1924)	0.5208	0.9986	0.0542 (-0.0968, 0.2052)	0.4787	0.9993
MHiBP	Glycine	0.0612 (-0.0576, 0.1799)	0.3099	0.9986	0.0872 (-0.0346, 0.2091)	0.1589	0.9993
MHiBP	Hypoxanthine	-0.0133 (-0.1254, 0.0988)	0.8146	0.9986	0.0023 (-0.1125, 0.1172)	0.9679	0.9993
MHiBP	Inosine	-0.0606 (-0.3224, 0.2013)	0.6479	0.9986	-0.0754 (-0.3415, 0.1907)	0.5758	0.9993
MHiBP	Isoleucine	0.0359 (-0.0952, 0.1671)	0.5888	0.9986	0.0608 (-0.0731, 0.1946)	0.3706	0.9993
MHiBP	Kynurenine	-0.2033 (-0.4023, -0.0043)	0.0454	0.9986	-0.1905 (-0.3969, 0.0159)	0.0701	0.9993
MHiBP	Lactate	0.0819 (-0.0020, 0.1658)	0.0556	0.9986	0.0897 (0.0029, 0.1764)	0.0429	0.9993
MHiBP	Leucine	0.0360 (-0.0999, 0.1720)	0.6009	0.9986	0.0635 (-0.0747, 0.2018)	0.3648	0.9993
MHiBP	Lysine	-0.0092 (-0.1634, 0.1451)	0.9066	0.9986	0.0195 (-0.1380, 0.1771)	0.8064	0.9993
MHiBP	Methionine	0.0512 (-0.0929, 0.1952)	0.4834	0.9986	0.0755 (-0.0703, 0.2212)	0.3075	0.9993
MHiBP	myo-Inositol	0.0466 (-0.0832, 0.1764)	0.4790	0.9986	0.0490 (-0.0842, 0.1823)	0.4676	0.9993
MHiBP	N-Acetylneuraminate	-0.0995 (-0.2566, 0.0576)	0.2125	0.9986	-0.0897 (-0.2534, 0.0740)	0.2801	0.9993
MHiBP	NAD+	-0.0765 (-0.3749, 0.2220)	0.6131	0.9986	-0.0869 (-0.3956, 0.2219)	0.5785	0.9993
MHiBP	Niacinamide	0.4123 (-0.1423, 0.9670)	0.1437	0.9986	0.4052 (-0.1668, 0.9773)	0.1633	0.9993
MHiBP	O-Acetylcarnitine	-0.0294 (-0.1674, 0.1086)	0.6739	0.9986	-0.0216 (-0.1654, 0.1222)	0.7664	0.9993
MHiBP	O-Phosphocholine	-0.1092 (-0.4672, 0.2487)	0.5470	0.9986	-0.1131 (-0.4868, 0.2606)	0.5502	0.9993
MHiBP	O-Phosphoethanolamine	-0.0155 (-0.2726, 0.2416)	0.9052	0.9986	0.0204 (-0.2432, 0.2840)	0.8785	0.9993
MHiBP	Ornithine	0.0587 (-0.1409, 0.2582)	0.5617	0.9986	0.0817 (-0.1241, 0.2875)	0.4335	0.9993
MHiBP	Pantothenate	-0.0152 (-0.1932, 0.1628)	0.8660	0.9986	-0.0544 (-0.2369, 0.1282)	0.5565	0.9993
MHiBP	Phenylalanine	0.0430 (-0.0947, 0.1808)	0.5374	0.9986	0.0686 (-0.0719, 0.2091)	0.3355	0.9993
MHiBP	Proline	0.0610 (-0.0567, 0.1786)	0.3071	0.9986	0.0772 (-0.0436, 0.1980)	0.2082	0.9993
MHiBP	Pyroglutamate	-0.0499 (-0.2130, 0.1132)	0.5458	0.9986	-0.0572 (-0.2275, 0.1130)	0.5069	0.9993
MHiBP	Serine	0.0310 (-0.1094, 0.1714)	0.6626	0.9986	0.0548 (-0.0880, 0.1976)	0.4488	0.9993
MHiBP	sn-Glycero-3-phosphocholine	0.0339 (-0.1853, 0.2531)	0.7603	0.9986	0.0874 (-0.1307, 0.3055)	0.4292	0.9993
MHiBP	Succinate	0.0140 (-0.2337, 0.2617)	0.9112	0.9986	0.0234 (-0.2327, 0.2796)	0.8567	0.9993
MHiBP	Taurine	-0.0086 (-0.1144, 0.0972)	0.8725	0.9986	0.0019 (-0.1077, 0.1114)	0.9733	0.9993
MHiBP	Threonine	0.0781 (-0.0487, 0.2050)	0.2252	0.9986	0.1063 (-0.0214, 0.2339)	0.1018	0.9993
MHiBP	Tryptophan	0.0036 (-0.2187, 0.2258)	0.9748	0.9986	0.0264 (-0.2016, 0.2543)	0.8192	0.9993
MHiBP	Tyrosine	0.0627 (-0.0662, 0.1915)	0.3374	0.9986	0.0883 (-0.0428, 0.2194)	0.1847	0.9993
MHiBP	Uracil	0.0645 (-0.1191, 0.2481)	0.4884	0.9986	0.0678 (-0.1224, 0.2580)	0.4816	0.9993
MHiBP	Uridine	-0.1246 (-0.3616, 0.1123)	0.2999	0.9986	-0.0962 (-0.3418, 0.1495)	0.4398	0.9993
MHiBP	Valine	0.0324 (-0.0889, 0.1537)	0.5977	0.9986	0.0559 (-0.0676, 0.1794)	0.3717	0.9993

Phthalate	Metabolite	Total Effect Estimate (95% CI)	p	FDR p	Direct Effect Estimate (95% CI)	p	FDR p
MiBP	1,3-Dihydroxyacetone	-0.1639 (-0.4056, 0.0779)	0.1822	0.9986	-0.1984 (-0.4514, 0.0547)	0.1233	0.9993
MiBP	2-Hydroxybutyrate	-0.0620 (-0.1814, 0.0574)	0.3059	0.9986	-0.0861 (-0.2102, 0.0380)	0.1720	0.9993
MiBP	3-Hydroxybutyrate	0.0266 (-0.2435, 0.2967)	0.8457	0.9986	0.0684 (-0.2086, 0.3454)	0.6258	0.9993
MiBP	4-Aminobutyrate	0.0358 (-0.1153, 0.1869)	0.6397	0.9986	0.0418 (-0.1092, 0.1929)	0.5847	0.9993
MiBP	4-Hydroxybutyrate	0.1979 (-0.1445, 0.5403)	0.2548	0.9986	0.1889 (-0.1710, 0.5489)	0.3008	0.9993
MiBP	Acetate	-0.0505 (-0.2605, 0.1594)	0.6346	0.9986	-0.0333 (-0.2524, 0.1858)	0.7642	0.9993
MiBP	Alanine	0.0247 (-0.0700, 0.1193)	0.6071	0.9986	0.0458 (-0.0505, 0.1420)	0.3485	0.9993
MiBP	Arginine	0.0013 (-0.1252, 0.1278)	0.9837	0.9986	0.0116 (-0.1203, 0.1436)	0.8617	0.9993
MiBP	Asparagine	0.0092 (-0.1013, 0.1198)	0.8689	0.9986	0.0400 (-0.0726, 0.1526)	0.4828	0.9993
MiBP	Aspartate	-0.0050 (-0.1019, 0.0919)	0.9189	0.9986	0.0071 (-0.0936, 0.1077)	0.8897	0.9993
MiBP	Betaine	0.0075 (-0.1025, 0.1175)	0.8930	0.9986	0.0246 (-0.0896, 0.1387)	0.6707	0.9993
MiBP	Carnitine	-0.0252 (-0.1210, 0.0706)	0.6035	0.9986	-0.0220 (-0.1223, 0.0782)	0.6643	0.9993
MiBP	Choline	0.0097 (-0.0930, 0.1125)	0.8514	0.9986	0.0168 (-0.0906, 0.1242)	0.7576	0.9993
MiBP	Creatine	0.0280 (-0.0537, 0.1097)	0.4989	0.9986	0.0322 (-0.0508, 0.1152)	0.4444	0.9993
MiBP	Cystine	0.1532 (-0.0510, 0.3575)	0.1401	0.9986	0.1520 (-0.0623, 0.3664)	0.1629	0.9993
MiBP	Ethanolamine	-0.0352 (-0.1420, 0.0717)	0.5158	0.9986	-0.0268 (-0.1378, 0.0843)	0.6343	0.9993
MiBP	Formate	-0.0901 (-0.2082, 0.0280)	0.1337	0.9986	-0.0726 (-0.1934, 0.0482)	0.2363	0.9993
MiBP	Fumarate	-0.1276 (-0.2772, 0.0221)	0.0940	0.9986	-0.1319 (-0.2885, 0.0247)	0.0980	0.9993
MiBP	Glucitol	-0.1132 (-0.2318, 0.0054)	0.0612	0.9986	-0.0973 (-0.2212, 0.0266)	0.1228	0.9993
MiBP	Glutamate	0.0090 (-0.0807, 0.0988)	0.8425	0.9986	0.0280 (-0.0648, 0.1208)	0.5517	0.9993
MiBP	Glutamine	0.0042 (-0.0575, 0.0660)	0.8921	0.9986	0.0134 (-0.0486, 0.0754)	0.6696	0.9993
MiBP	Glutathione	-0.1920 (-0.4203, 0.0363)	0.0986	0.9986	-0.1849 (-0.4202, 0.0503)	0.1222	0.9993
MiBP	Glycerol	0.0250 (-0.0938, 0.1437)	0.6783	0.9986	0.0337 (-0.0904, 0.1577)	0.5917	0.9993
MiBP	Glycine	0.0404 (-0.0568, 0.1376)	0.4125	0.9986	0.0657 (-0.0345, 0.1658)	0.1967	0.9993
MiBP	Hypoxanthine	-0.0191 (-0.1107, 0.0725)	0.6805	0.9986	-0.0003 (-0.0946, 0.0940)	0.9946	0.9998
MiBP	Inosine	-0.0840 (-0.2978, 0.1297)	0.4380	0.9986	-0.0980 (-0.3160, 0.1200)	0.3753	0.9993
MiBP	Isoleucine	0.0302 (-0.0771, 0.1374)	0.5786	0.9986	0.0583 (-0.0514, 0.1680)	0.2951	0.9993
MiBP	Kynurenine	-0.2190 (-0.3798, -0.0582)	0.0080	0.9986	-0.2014 (-0.3693, -0.0335)	0.0191	0.9993
MiBP	Lactate	0.0684 (-0.0002, 0.1369)	0.0506	0.9986	0.0768 (0.0057, 0.1479)	0.0345	0.9993
MiBP	Leucine	0.0318 (-0.0793, 0.1429)	0.5721	0.9986	0.0627 (-0.0506, 0.1760)	0.2755	0.9993
MiBP	Lysine	-0.0073 (-0.1335, 0.1188)	0.9085	0.9986	0.0237 (-0.1056, 0.1530)	0.7174	0.9993
MiBP	Methionine	0.0502 (-0.0675, 0.1678)	0.4003	0.9986	0.0822 (-0.0371, 0.2014)	0.1750	0.9993
MiBP	myo-Inositol	0.0565 (-0.0494, 0.1624)	0.2929	0.9986	0.0575 (-0.0516, 0.1666)	0.2987	0.9993
MiBP	N-Acetylneuraminate	-0.1272 (-0.2545, 0.0001)	0.0503	0.9986	-0.1162 (-0.2495, 0.0172)	0.0872	0.9993
MiBP	NAD+	-0.1111 (-0.3546, 0.1323)	0.3682	0.9986	-0.1141 (-0.3671, 0.1388)	0.3734	0.9993
MiBP	Niacinamide	0.3759 (-0.0766, 0.8284)	0.1027	0.9986	0.3616 (-0.1073, 0.8304)	0.1294	0.9993
MiBP	O-Acetylcarnitine	-0.0454 (-0.1579, 0.0672)	0.4269	0.9986	-0.0349 (-0.1529, 0.0830)	0.5588	0.9993
MiBP	O-Phosphocholine	-0.1330 (-0.4251, 0.1591)	0.3693	0.9986	-0.1294 (-0.4357, 0.1769)	0.4046	0.9993
MiBP	O-Phosphoethanolamine	-0.0241 (-0.2342, 0.1861)	0.8209	0.9986	0.0116 (-0.2048, 0.2280)	0.9159	0.9993
MiBP	Ornithine	0.0813 (-0.0814, 0.2440)	0.3246	0.9986	0.1007 (-0.0677, 0.2691)	0.2388	0.9993
MiBP	Pantothenate	-0.0187 (-0.1642, 0.1268)	0.7997	0.9986	-0.0531 (-0.2028, 0.0967)	0.4844	0.9993
MiBP	Phenylalanine	0.0261 (-0.0866, 0.1388)	0.6477	0.9986	0.0544 (-0.0610, 0.1697)	0.3525	0.9993
MiBP	Proline	0.0559 (-0.0402, 0.1520)	0.2519	0.9986	0.0758 (-0.0231, 0.1747)	0.1316	0.9993
MiBP	Pyroglutamate	-0.0258 (-0.1592, 0.1076)	0.7027	0.9986	-0.0355 (-0.1754, 0.1043)	0.6161	0.9993
MiBP	Serine	0.0149 (-0.0999, 0.1298)	0.7972	0.9986	0.0452 (-0.0720, 0.1624)	0.4468	0.9993
MiBP	sn-Glycero-3-phosphocholine	0.0402 (-0.1390, 0.2193)	0.6580	0.9986	0.1033 (-0.0752, 0.2818)	0.2542	0.9993
MiBP	Succinate	0.0228 (-0.1797, 0.2253)	0.8238	0.9986	0.0422 (-0.1680, 0.2523)	0.6919	0.9993
MiBP	Taurine	-0.0318 (-0.1181, 0.0546)	0.4678	0.9986	-0.0207 (-0.1106, 0.0691)	0.6485	0.9993
MiBP	Threonine	0.0289 (-0.0753, 0.1331)	0.5845	0.9986	0.0617 (-0.0436, 0.1671)	0.2484	0.9993
MiBP	Tryptophan	0.0660 (-0.1153, 0.2473)	0.4726	0.9986	0.0897 (-0.0967, 0.2762)	0.3427	0.9993
MiBP	Tyrosine	0.0488 (-0.0566, 0.1542)	0.3613	0.9986	0.0795 (-0.0279, 0.1869)	0.1455	0.9993
MiBP	Uracil	0.0495 (-0.1006, 0.1996)	0.5153	0.9986	0.0577 (-0.0984, 0.2138)	0.4656	0.9993
MiBP	Uridine	-0.1434 (-0.3363, 0.0495)	0.1438	0.9986	-0.1179 (-0.3190, 0.0831)	0.2477	0.9993
MiBP	Valine	0.0260 (-0.0731, 0.1252)	0.6043	0.9986	0.0521 (-0.0492, 0.1534)	0.3104	0.9993
MNP	1,3-Dihydroxyacetone	0.0477 (-0.1929, 0.2884)	0.6955	0.9986	0.0374 (-0.2198, 0.2947)	0.7737	0.9993
MNP	2-Hydroxybutyrate	-0.0320 (-0.1504, 0.0865)	0.5942	0.9986	-0.0333 (-0.1590, 0.0925)	0.6014	0.9993
MNP	3-Hydroxybutyrate	-0.0425 (-0.3096, 0.2245)	0.7532	0.9986	0.0056 (-0.2736, 0.2848)	0.9686	0.9993
MNP	4-Aminobutyrate	-0.0266 (-0.1760, 0.1229)	0.7256	0.9986	-0.0581 (-0.2101, 0.0938)	0.4504	0.9993
MNP	4-Hydroxybutyrate	0.0662 (-0.2739, 0.4064)	0.7006	0.9986	0.0970 (-0.2667, 0.4607)	0.5984	0.9993
MNP	Acetate	-0.0354 (-0.2432, 0.1723)	0.7363	0.9986	-0.0213 (-0.2420, 0.1994)	0.8491	0.9993

		Total Effect			Direct Effect		
**Phthalate**	**Metabolite**	**Estimate (95% CI)**	**p**	**FDR p**	**Estimate (95% CI)**	**p**	**FDR p**
MNP	Alanine	-0.0120 (-0.1057, 0.0817)	0.7999	0.9986	-0.0154 (-0.1127, 0.0818)	0.7541	0.9993
MNP	Arginine	-0.0620 (-0.1866, 0.0626)	0.3264	0.9986	-0.0667 (-0.1990, 0.0657)	0.3207	0.9993
MNP	Asparagine	-0.0558 (-0.1647, 0.0531)	0.3123	0.9986	-0.0577 (-0.1709, 0.0554)	0.3146	0.9993
MNP	Aspartate	-0.0710 (-0.1660, 0.0241)	0.1420	0.9986	-0.0685 (-0.1691, 0.0321)	0.1800	0.9993
MNP	Betaine	-0.1135 (-0.2204, -0.0066)	0.0377	0.9986	-0.1204 (-0.2333, -0.0074)	0.0369	0.9993
MNP	Carnitine	-0.0594 (-0.1537, 0.0348)	0.2145	0.9986	-0.0767 (-0.1768, 0.0234)	0.1317	0.9993
MNP	Choline	-0.0284 (-0.1299, 0.0731)	0.5804	0.9986	-0.0246 (-0.1327, 0.0835)	0.6536	0.9993
MNP	Creatine	-0.0014 (-0.0823, 0.0795)	0.9730	0.9986	0.0061 (-0.0777, 0.0899)	0.8855	0.9993
MNP	Cystine	0.0411 (-0.1625, 0.2447)	0.6900	0.9986	0.0476 (-0.1699, 0.2651)	0.6657	0.9993
MNP	Ethanolamine	0.0089 (-0.0970, 0.1147)	0.8688	0.9986	-0.0034 (-0.1153, 0.1086)	0.9525	0.9993
MNP	Formate	-0.0516 (-0.1692, 0.0659)	0.3865	0.9986	-0.0409 (-0.1630, 0.0813)	0.5087	0.9993
MNP	Fumarate	-0.0686 (-0.2177, 0.0805)	0.3644	0.9986	-0.0713 (-0.2303, 0.0877)	0.3764	0.9993
MNP	Glucitol	-0.0568 (-0.1753, 0.0617)	0.3449	0.9986	-0.0459 (-0.1717, 0.0799)	0.4715	0.9993
MNP	Glutamate	-0.0078 (-0.0966, 0.0810)	0.8626	0.9986	0.0012 (-0.0924, 0.0948)	0.9794	0.9993
MNP	Glutamine	-0.0353 (-0.0961, 0.0255)	0.2525	0.9986	-0.0487 (-0.1106, 0.0131)	0.1213	0.9993
MNP	Glutathione	-0.2156 (-0.4407, 0.0095)	0.0603	0.9986	-0.2467 (-0.4817, -0.0116)	0.0399	0.9993
MNP	Glycerol	-0.0269 (-0.1443, 0.0906)	0.6516	0.9986	-0.0239 (-0.1489, 0.1011)	0.7059	0.9993
MNP	Glycine	-0.0217 (-0.1180, 0.0746)	0.6567	0.9986	-0.0122 (-0.1137, 0.0894)	0.8128	0.9993
MNP	Hypoxanthine	-0.0249 (-0.1154, 0.0656)	0.5872	0.9986	-0.0277 (-0.1226, 0.0671)	0.5638	0.9993
MNP	Inosine	0.0215 (-0.1904, 0.2334)	0.8411	0.9986	0.0200 (-0.2002, 0.2402)	0.8576	0.9993
MNP	Isoleucine	-0.0278 (-0.1338, 0.0783)	0.6054	0.9986	-0.0212 (-0.1321, 0.0898)	0.7063	0.9993
MNP	Kynurenine	-0.1437 (-0.3053, 0.0178)	0.0807	0.9986	-0.1617 (-0.3322, 0.0088)	0.0629	0.9993
MNP	Lactate	0.0188 (-0.0500, 0.0876)	0.5893	0.9986	0.0152 (-0.0577, 0.0881)	0.6798	0.9993
MNP	Leucine	-0.0371 (-0.1469, 0.0728)	0.5057	0.9986	-0.0310 (-0.1455, 0.0836)	0.5932	0.9993
MNP	Lysine	-0.0285 (-0.1531, 0.0962)	0.6519	0.9986	-0.0204 (-0.1506, 0.1098)	0.7572	0.9993
MNP	Methionine	-0.0082 (-0.1248, 0.1085)	0.8899	0.9986	0.0044 (-0.1166, 0.1254)	0.9427	0.9993
MNP	myo-Inositol	0.0374 (-0.0675, 0.1424)	0.4816	0.9986	0.0597 (-0.0502, 0.1695)	0.2843	0.9993
MNP	N-Acetylneuraminate	-0.1335 (-0.2591, -0.0078)	0.0375	0.9986	-0.1320 (-0.2659, 0.0018)	0.0532	0.9993
MNP	NAD+	-0.0726 (-0.3138, 0.1687)	0.5527	0.9986	-0.1192 (-0.3739, 0.1354)	0.3557	0.9993
MNP	Niacinamide	0.1726 (-0.2787, 0.6239)	0.4506	0.9986	0.1533 (-0.3226, 0.6293)	0.5247	0.9993
MNP	O-Acetylcarnitine	-0.0507 (-0.1620, 0.0606)	0.3688	0.9986	-0.0671 (-0.1854, 0.0512)	0.2638	0.9993
MNP	O-Phosphocholine	-0.1612 (-0.4496, 0.1272)	0.2708	0.9986	-0.2231 (-0.5299, 0.0836)	0.1524	0.9993
MNP	O-Phosphoethanolamine	-0.1654 (-0.3713, 0.0404)	0.1142	0.9986	-0.1587 (-0.3747, 0.0573)	0.1484	0.9993
MNP	Ornithine	0.0050 (-0.1566, 0.1665)	0.9515	0.9986	0.0280 (-0.1425, 0.1984)	0.7460	0.9993
MNP	Pantothenate	0.0308 (-0.1130, 0.1747)	0.6724	0.9986	-0.0090 (-0.1601, 0.1422)	0.9068	0.9993
MNP	Phenylalanine	-0.0746 (-0.1854, 0.0361)	0.1849	0.9986	-0.0673 (-0.1832, 0.0487)	0.2529	0.9993
MNP	Proline	-0.0142 (-0.1097, 0.0813)	0.7688	0.9986	-0.0105 (-0.1110, 0.0900)	0.8359	0.9993
MNP	Pyroglutamate	-0.0359 (-0.1678, 0.0959)	0.5905	0.9986	-0.0362 (-0.1770, 0.1047)	0.6121	0.9993
MNP	Serine	-0.0491 (-0.1624, 0.0642)	0.3925	0.9986	-0.0436 (-0.1616, 0.0745)	0.4662	0.9993
MNP	sn-Glycero-3-phosphocholine	-0.0297 (-0.2069, 0.1475)	0.7406	0.9986	-0.0374 (-0.2180, 0.1432)	0.6825	0.9993
MNP	Succinate	0.1646 (-0.0336, 0.3628)	0.1028	0.9986	0.1649 (-0.0448, 0.3746)	0.1221	0.9993
MNP	Taurine	0.0108 (-0.0747, 0.0964)	0.8026	0.9986	0.0071 (-0.0834, 0.0976)	0.8770	0.9993
MNP	Threonine	-0.0459 (-0.1487, 0.0570)	0.3794	0.9986	-0.0390 (-0.1455, 0.0675)	0.4698	0.9993
MNP	Tryptophan	0.1021 (-0.0767, 0.2809)	0.2606	0.9986	0.1442 (-0.0424, 0.3309)	0.1287	0.9993
MNP	Tyrosine	-0.0202 (-0.1247, 0.0843)	0.7032	0.9986	-0.0139 (-0.1230, 0.0952)	0.8011	0.9993
MNP	Uracil	-0.0150 (-0.1637, 0.1337)	0.8422	0.9986	-0.0154 (-0.1729, 0.1420)	0.8463	0.9993
MNP	Uridine	0.0548 (-0.1374, 0.2470)	0.5738	0.9986	0.0897 (-0.1132, 0.2927)	0.3830	0.9993
MNP	Valine	-0.0358 (-0.1338, 0.0622)	0.4713	0.9986	-0.0287 (-0.1310, 0.0736)	0.5796	0.9993

Table S7: Associations between placenta metabolites and birth year. In samples with a molar sum of urinary DEHP metabolites below the median (n = 66), we conducted multiple linear regressions to measure the association between each $\log_{10}$-transformed placenta metabolite concentration and birth year after adjustment for gestational age at delivery, maternal metabolic condition, homeownership status, maternal race/ethnicity, and maternal education. We conducted permutation testing under the reduced model using 4999 permutations for each metabolite and corrected for false discovery rate (FDR). Only metabolites with FDR *p*-values ≥; 0.1 are reported here.

Metabolite	Coefficient (95% CI)	R^2	p	FDR p
1,3-Dihydroxyacetone	-0.0220 (-0.1231, 0.0790)	0.0030	0.6668	0.7502
2-Hydroxybutyrate	0.0118 (-0.0360, 0.0595)	0.0038	0.6134	0.7421
3-Hydroxybutyrate	0.0030 (-0.1019, 0.1078)	0.0000	0.9558	0.9738
4-Aminobutyrate	-0.0153 (-0.0670, 0.0364)	0.0054	0.5618	0.7421
4-Hydroxybutyrate	-0.0384 (-0.1330, 0.0561)	0.0102	0.4234	0.6666
Acetate	0.0150 (-0.0437, 0.0738)	0.0041	0.6068	0.7421
Alanine	-0.0269 (-0.0636, 0.0098)	0.0324	0.1460	0.3072
Asparagine	-0.0408 (-0.0792, -0.0025)	0.0661	0.0392	0.1058
Aspartate	0.0097 (-0.0228, 0.0421)	0.0055	0.5724	0.7421
Betaine	0.0273 (-0.0164, 0.0710)	0.0238	0.2098	0.3907
Carnitine	0.0156 (-0.0189, 0.0502)	0.0126	0.3762	0.6348
Choline	-0.0085 (-0.0432, 0.0262)	0.0037	0.6322	0.7421
Ethanolamine	-0.0140 (-0.0505, 0.0224)	0.0092	0.4444	0.6666
Formate	-0.0075 (-0.0394, 0.0244)	0.0035	0.6312	0.7421
Fumarate	0.0636 (-0.0056, 0.1327)	0.0500	0.0622	0.1460
Glucitol	0.0014 (-0.0422, 0.0451)	0.0001	0.9420	0.9738
Glutamate	-0.0078 (-0.0380, 0.0223)	0.0042	0.6138	0.7421
Glutamine	0.0086 (-0.0174, 0.0346)	0.0068	0.5244	0.7421
Glycerol	-0.0282 (-0.0667, 0.0103)	0.0324	0.1536	0.3072
Glycine	-0.0342 (-0.0690, 6e-04)	0.0569	0.0538	0.1321
Hypoxanthine	-0.0263 (-0.0625, 0.0100)	0.0317	0.1492	0.3072
Kynurenine	-1e-04 (-0.0666, 0.0665)	0.0000	0.9974	0.9974
Lactate	-0.0069 (-0.0334, 0.0195)	0.0043	0.6052	0.7421
myo-Inositol	0.0058 (-0.0274, 0.0390)	0.0019	0.7360	0.8111
N-Acetylneuraminate	0.0167 (-0.0281, 0.0616)	0.0086	0.4444	0.6666
Niacinamide	0.0185 (-0.1348, 0.1717)	0.0009	0.8028	0.8500
O-Acetylcarnitine	0.0061 (-0.0350, 0.0472)	0.0014	0.7706	0.8322
Pantothenate	-0.0160 (-0.0732, 0.0413)	0.0048	0.5844	0.7421
Proline	-0.0247 (-0.0601, 0.0107)	0.0295	0.1636	0.3155
sn-Glycero-3-phosphocholine	-0.0572 (-0.1290, 0.0145)	0.0382	0.1232	0.2772
Succinate	-0.0097 (-0.0546, 0.0352)	0.0029	0.6568	0.7502
Taurine	0.0144 (-0.0143, 0.0431)	0.0154	0.3266	0.5689
Threonine	-0.0151 (-0.0537, 0.0235)	0.0095	0.4302	0.6666
Uridine	-0.0261 (-0.0746, 0.0225)	0.0177	0.2884	0.5191
Valine	-0.0333 (-0.0673, 7e-04)	0.0564	0.0532	0.1321

Table S8: Associations between placenta metabolites and neurodevelopmental outcomes in all children and stratified by sex. Single-response PERMANOVA was used to assess the association between each $\log_{10}$-transformed serum metabolite concentration and neurodevelopmental outcome after adjustment for birth year, birth weight, gestational age at delivery, delivery mode, homeownership status, prenatal vitamin use in the first month of pregnancy, maternal age, maternal metabolic condition, and maternal race/ethnicity. Significance was assessed using permutation testing under 4999 permutations and *p*-values were corrected for false discovery rate (FDR).

	All Children						Males				Females			
**Metabolite**	***F***	R^2	***p***	**FDR *p***	**Interaction *p***	**Interaction FDR *p***	***F***	R^2	***p***	**FDR *p***	***F***	R^2	***p***	**FDR *p***
1,3-Dihydroxyacetone	2.32	0.0363	0.0948	0.3470	0.6806	0.9396	2.65	0.0669	0.0824	0.2462	0.70	0.0283	0.4980	0.8424
2-Hydroxybutyrate	1.37	0.0221	0.2670	0.4651	0.7282	0.9396	0.70	0.0197	0.4956	0.6082	1.55	0.0536	0.2160	0.8424
3-Hydroxybutyrate	0.88	0.0134	0.4272	0.5948	0.4964	0.9396	0.09	0.0025	0.9152	0.9325	2.03	0.0644	0.1464	0.8273
4-Aminobutyrate	0.07	0.0011	0.9334	0.9334	0.1640	0.8856	0.48	0.0118	0.6208	0.7176	0.78	0.0269	0.4648	0.8424
4-Hydroxybutyrate	0.63	0.0098	0.5372	0.6593	0.8386	0.9396	0.27	0.0074	0.7702	0.8146	0.05	0.0019	0.9550	0.9902
Acetate	0.29	0.0047	0.7458	0.8055	0.3374	0.9114	1.88	0.0525	0.1588	0.3427	0.84	0.0306	0.4322	0.8424
Alanine	2.24	0.0292	0.1102	0.3470	0.3052	0.9114	3.17	0.0718	0.0466	0.2462	0.16	0.0053	0.8506	0.9569
Arginine	0.82	0.0124	0.4472	0.6037	0.0922	0.6224	2.05	0.0500	0.1318	0.2966	0.58	0.0205	0.5654	0.8424
Asparagine	1.22	0.0168	0.3140	0.5138	0.6654	0.9396	1.18	0.0270	0.3156	0.4606	0.80	0.0290	0.4454	0.8424
Aspartate	1.63	0.0255	0.2102	0.4054	0.7326	0.9396	1.41	0.0391	0.2594	0.4377	0.94	0.0396	0.4106	0.8424
Betaine	0.50	0.0076	0.6118	0.7342	0.7016	0.9396	0.25	0.0067	0.7844	0.8146	0.18	0.0054	0.8360	0.9569
Carnitine	0.83	0.0130	0.4296	0.5948	0.5990	0.9396	0.26	0.0074	0.7626	0.8146	2.98	0.1127	0.0626	0.6761
Choline	1.88	0.0286	0.1542	0.3470	0.9406	0.9406	1.54	0.0424	0.2272	0.3958	4.02	0.1256	0.0292	0.5256
Creatine	2.19	0.0313	0.1158	0.3470	0.8990	0.9396	1.55	0.0414	0.2200	0.3958	1.09	0.0359	0.3564	0.8424
Cystine	2.08	0.0263	0.1310	0.3470	0.0356	0.4806	4.25	0.0868	0.0198	0.2114	1.00	0.0336	0.3734	0.8424
Ethanolamine	2.92	0.0415	0.0584	0.3470	0.6226	0.9396	3.30	0.0783	0.0478	0.2462	2.04	0.0670	0.1532	0.8273
Formate	0.44	0.0065	0.6370	0.7478	0.6976	0.9396	0.49	0.0132	0.6246	0.7176	0.24	0.0084	0.7468	0.9393
Fumarate	3.04	0.0460	0.0534	0.3470	0.2014	0.9114	4.45	0.1157	0.0192	0.2114	0.07	0.0028	0.9268	0.9902
Glucitol	0.77	0.0121	0.4644	0.6116	0.8956	0.9396	0.34	0.0103	0.7004	0.7811	1.20	0.0479	0.3082	0.8424
Glutamate	2.77	0.0403	0.0714	0.3470	0.4682	0.9396	2.96	0.0759	0.0536	0.2462	0.77	0.0273	0.4646	0.8424
Glutamine	1.13	0.0165	0.3304	0.5248	0.7580	0.9396	1.29	0.0319	0.2800	0.4582	0.01	0.0005	0.9902	0.9902
Glutathione	0.68	0.0081	0.5070	0.6367	0.0814	0.6224	3.13	0.0605	0.0598	0.2462	0.91	0.0282	0.4152	0.8424
Glycerol	2.40	0.0338	0.0916	0.3470	0.8680	0.9396	1.82	0.0454	0.1650	0.3427	4.06	0.1232	0.0246	0.5256
Glycine	2.41	0.0308	0.0866	0.3470	0.3744	0.9114	2.84	0.0632	0.0648	0.2462	0.32	0.0107	0.7328	0.9393
Hypoxanthine	1.85	0.0260	0.1620	0.3499	0.2410	0.9114	3.82	0.0926	0.0252	0.2114	0.03	0.0012	0.9612	0.9902
Inosine	0.88	0.0098	0.4162	0.5948	0.2278	0.9114	1.66	0.0342	0.2040	0.3934	0.28	0.0081	0.7716	0.9470
Isoleucine	2.37	0.0296	0.1014	0.3470	0.6022	0.9396	2.70	0.0579	0.0782	0.2462	0.65	0.0204	0.5270	0.8424
Kynurenine	0.82	0.0131	0.4186	0.5948	0.3488	0.9114	1.22	0.0325	0.3048	0.4606	0.12	0.0040	0.8868	0.9773
Lactate	1.35	0.0205	0.2638	0.4651	0.8834	0.9396	1.08	0.0288	0.3498	0.4843	0.52	0.0181	0.5998	0.8424
Leucine	2.04	0.0249	0.1314	0.3470	0.4990	0.9396	2.51	0.0513	0.0912	0.2462	0.50	0.0159	0.6084	0.8424
Lysine	1.98	0.0255	0.1482	0.3470	0.3760	0.9114	2.80	0.0599	0.0644	0.2462	0.54	0.0168	0.5826	0.8424
Methionine	2.75	0.0325	0.0702	0.3470	0.3882	0.9114	3.87	0.0761	0.0274	0.2114	0.44	0.0131	0.6482	0.8751
myo-Inositol	2.60	0.0383	0.0816	0.3470	0.9186	0.9396	1.20	0.0330	0.3076	0.4606	1.15	0.0450	0.3200	0.8424
N-Acetylneuraminate	1.30	0.0203	0.2776	0.4685	0.8600	0.9396	0.35	0.0097	0.7088	0.7811	3.03	0.1013	0.0562	0.6761
NAD+	0.69	0.0073	0.4986	0.6367	0.0020	0.0540	5.75	0.0901	0.0064	0.2114	1.96	0.0537	0.1460	0.8273
Niacinamide	3.13	0.0478	0.0420	0.3470	0.7696	0.9396	2.36	0.0629	0.0980	0.2520	1.01	0.0423	0.3688	0.8424
O-Acetylcarnitine	1.97	0.0305	0.1432	0.3470	0.7492	0.9396	1.05	0.0295	0.3480	0.4843	1.75	0.0644	0.1818	0.8424

	All Children						Males				Females			
**Metabolite**	***F***	***R*²**	***p***	**FDR *p***	**Interaction *p***	**Interaction FDR *p***	***F***	***R*²**	***p***	**FDR *p***	***F***	***R*²**	***p***	**FDR *p***
O-Phosphocholine	0.07	0.0009	0.9268	0.9334	0.0266	0.4788	0.83	0.0168	0.4456	0.5729	1.91	0.0510	0.1442	0.8273
O-Phosphoethanolamine	0.16	0.0019	0.8516	0.8941	0.0764	0.6224	0.84	0.0163	0.4312	0.5679	0.79	0.0240	0.4628	0.8424
Ornithine	1.35	0.0174	0.2650	0.4651	0.2468	0.9114	1.80	0.0389	0.1770	0.3540	0.51	0.0190	0.5972	0.8424
Pantothenate	0.40	0.0060	0.6742	0.7585	0.1178	0.7068	0.74	0.0197	0.4912	0.6082	2.05	0.0697	0.1312	0.8273
Phenylalanine	1.65	0.0207	0.1972	0.4054	0.2778	0.9114	2.54	0.0532	0.0898	0.2462	0.59	0.0190	0.5618	0.8424
Proline	2.05	0.0281	0.1380	0.3470	0.6180	0.9396	2.28	0.0558	0.1094	0.2569	0.18	0.0057	0.8418	0.9569
Pyroglutamate	0.15	0.0018	0.8610	0.8941	0.0010	0.0540	4.19	0.0766	0.0214	0.2114	1.52	0.0442	0.2300	0.8424
Serine	2.05	0.0255	0.1300	0.3470	0.3094	0.9114	2.68	0.0553	0.0824	0.2462	0.30	0.0106	0.7480	0.9393
sn-Glycero-3-phosphocholine	1.64	0.0231	0.2062	0.4054	0.9222	0.9396	0.87	0.0228	0.4236	0.5679	0.67	0.0255	0.5082	0.8424
Succinate	1.06	0.0165	0.3496	0.5394	0.7392	0.9396	0.65	0.0184	0.5232	0.6278	0.93	0.0339	0.4094	0.8424
Taurine	3.26	0.0471	0.0418	0.3470	0.6246	0.9396	1.16	0.0312	0.3112	0.4606	4.78	0.1690	0.0146	0.5256
Threonine	2.58	0.0327	0.0798	0.3470	0.8714	0.9396	2.41	0.0502	0.1028	0.2523	1.47	0.0488	0.2358	0.8424
Tryptophan	0.38	0.0049	0.6968	0.7679	0.7674	0.9396	0.05	0.0013	0.9544	0.9544	1.32	0.0392	0.2888	0.8424
Tyrosine	2.69	0.0341	0.0720	0.3470	0.6006	0.9396	2.80	0.0598	0.0660	0.2462	0.81	0.0279	0.4494	0.8424
Uracil	0.42	0.0057	0.6570	0.7549	0.3158	0.9114	1.56	0.0369	0.2132	0.3958	0.03	0.0007	0.9742	0.9902
Uridine	3.41	0.0504	0.0390	0.3470	0.0904	0.6224	4.01	0.1020	0.0208	0.2114	0.21	0.0076	0.8074	0.9569
Valine	2.27	0.0284	0.1074	0.3470	0.5594	0.9396	2.65	0.0565	0.0864	0.2462	0.53	0.0174	0.5818	0.8424

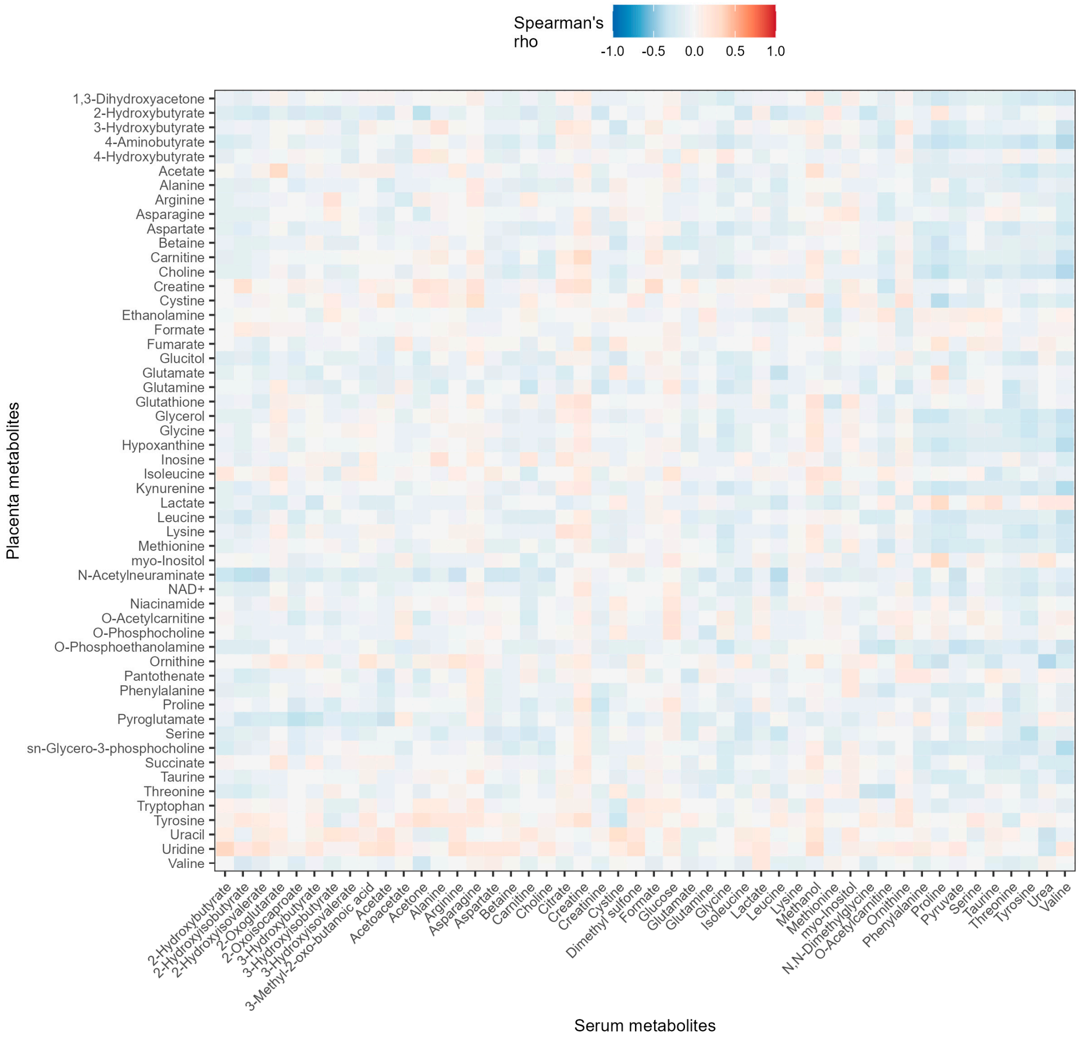

Figure S1. Spearman correlations between placental metabolites and serum metabolites. Positive associations (red) identify metabolites where higher placental metabolite concentrations correlated with higher serum metabolite concentrations, while negative associations (blue) identify metabolites where higher placental metabolite concentrations correlated with lower serum metabolite concentrations. No correlations remained significant after correction for false discovery rate.

A

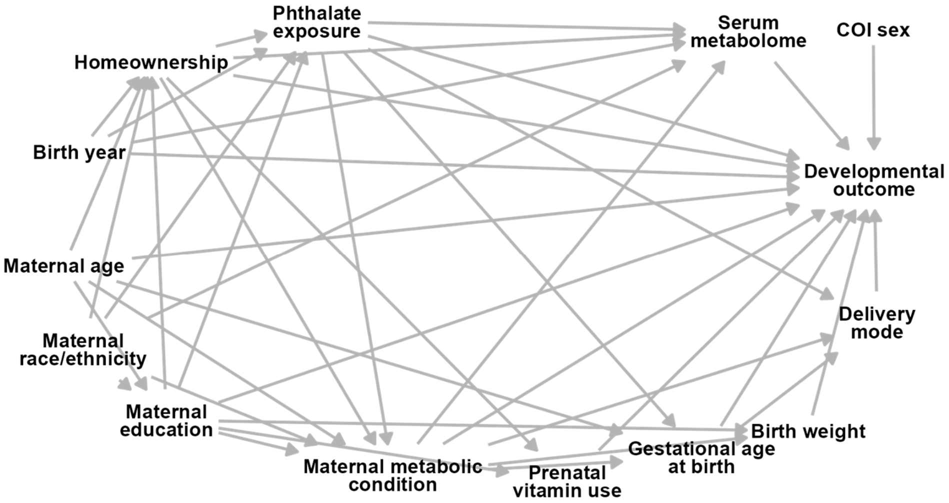

B

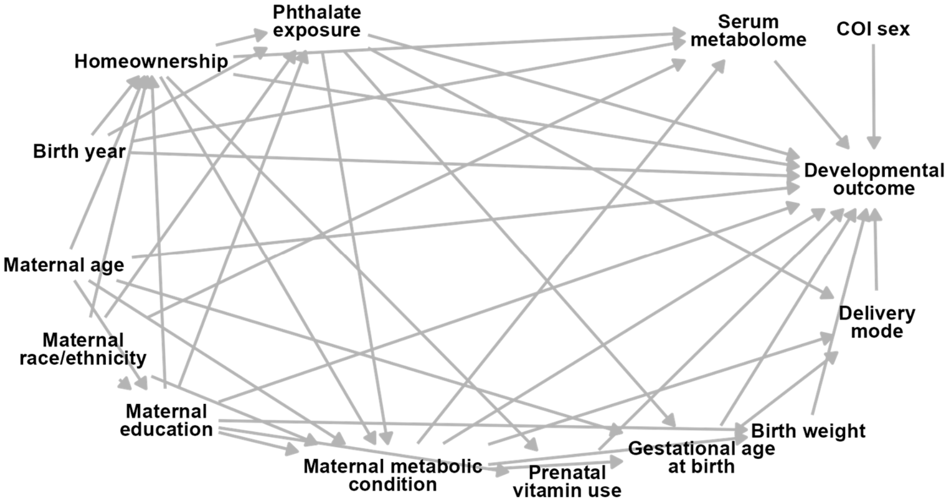

Figure S2. Directed acyclic graphs (DAGs) for the association between phthalate exposure, the serum metabolome, and neurodevelopmental outcomes. The DAG in (A) was used to evaluate the association between phthalate exposure and the serum metabolome and the DAG in (B), where the relationship between phthalate exposure and the serum metabolome was removed, was used to evaluate the association between the serum metabolome and neurodevelopmental outcome. COI = child of interest.

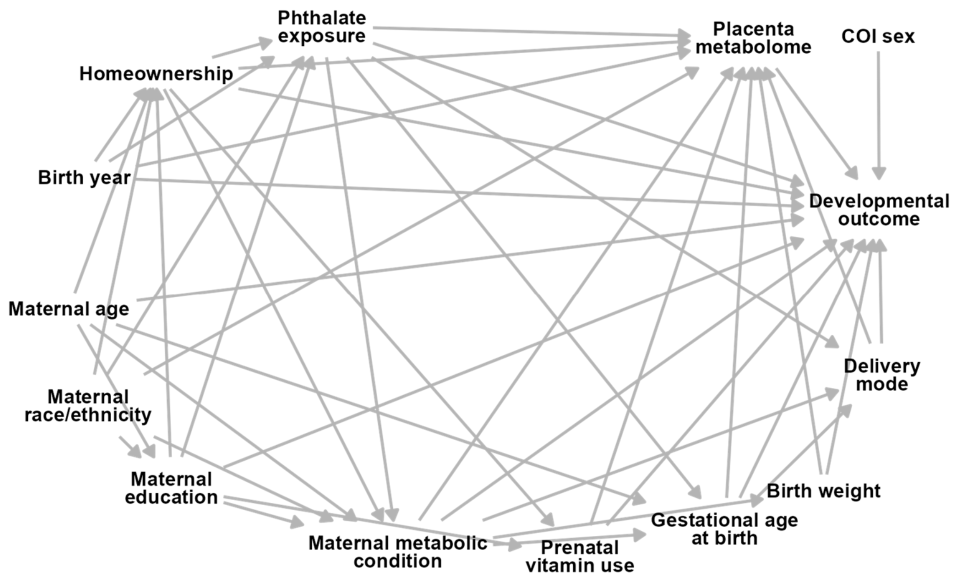

Figure S3. Directed acyclic graph (DAG) for the association between phthalate exposure, the placental metabolome, and neurodevelopmental outcomes. The DAG was used to evaluate the association between phthalate exposure and the placental metabolome and the association between the placental metabolome and neurodevelopmental outcome. COI = child of interest.
